# “In a tree by the brook, there’s a songbird who sings”: Woodlands in an agricultural matrix maintain functionality of a wintering bird community

**DOI:** 10.1371/journal.pone.0201657

**Published:** 2018-08-02

**Authors:** Biang La Nam Syiem, Varun R. Goswami, Divya Vasudev

**Affiliations:** 1 Post-graduate Programme in Wildlife Biology and Conservation, Wildlife Conservation Society India Program and National Centre for Biological Sciences, Bangalore, India; 2 Wildlife Conservation Society, India Program, Bangalore, India; 3 Centre for Wildlife Studies, Bangalore, India; 4 Conservation Initiatives, Guwahati, India; Centre for Cellular and Molecular Biology, INDIA

## Abstract

The agricultural matrix has increasingly been recognized for its potential to supplement Protected Areas (PAs) in biodiversity conservation. This potential is highly contextual, depending on composition and spatial configuration of matrix elements and their mechanistic relationship with biological communities. We investigate the effects of local vegetation structure, and proximity to a PA on the site-use of different guilds in a wintering bird community within the PA, and in wooded land-use types in the surrounding matrix. We used occupancy models to estimate covariate–guild relationships and predict site-use. We also compared species richness (estimated through capture–recapture models) and species naïve site-use between the PA and the matrix to evaluate taxonomic changes. We found that tree cover did not limit the site-use of most guilds of the community, probably due to high canopy cover across all chosen sites. Exceptions to this were guilds comprising generalist species. Shrub cover and bamboo cover had important effects on some woodland-associated guilds, suggesting a change in limiting factors for site-use under adequate tree cover. Site-use across the matrix was high for all analyzed guilds. This was found to be due to three non-exclusive reasons: (i) presence of one or more ubiquitous species (found all across the landscape) within some guilds, (ii) redundancy of species within guilds that buffered against a decrease in site-use, and (iii) turnover in guild composition/abundances to more generalist species from PA to matrix. Estimated species richness was higher in the matrix (107± 11; mean ± SE) than in the PA (90± 7), which may have been in part due to the addition of generalist species in the matrix. Understanding factors that limit biological communities is crucial to better managing the ever-increasing matrix for biodiversity conservation. Our study provides insights into the effects of different components of vegetation structure on the bird community in wooded land-use types in the matrix. We highlight the value of woodlands surrounding PAs in maintaining multiple guilds, and hence, the functionality of a wintering bird community. However, we caution that the matrix may fall short in retaining some specialized species of the community.

## Introduction

Agricultural expansion continues to be a major driver of large-scale conversion of tropical forests into human-use areas [1]. The resultant habitat loss is generally detrimental to biodiversity, leading to the loss of species, as well as community structure and function [2]. Efforts to prevent forest conversion and protect biodiversity in the tropics––including threatened wildlife species in particular––have largely best been met by the establishment of Protected Areas (PAs) [3]. However, under current rates of human population growth, associated land-use change and inefficient policy, the scope for expansion of PAs is greatly limited [4], with continuing conversion of forests into agricultural land. There is also increased recognition that PAs are not insular, and biodiversity within PAs interacts with agricultural land-uses outside [5]. As such, conservationists have brought to attention the need to evaluate and target the ever-increasing agricultural land-use types for their potential to supplement biodiversity conservation in the tropics.

Agricultural land-uses surrounding PAs, collectively termed the matrix (non-optimal habitat areas in conservation landscapes), are characterized by a mosaic of managed land-use types interspersed with natural and semi-natural habitats that vary in their ability to support biodiversity [6,7]. This conservation potential of the matrix is highly contextual, depending on the land-use type, taxon, spatial location and other factors. For example, biodiversity is better supported in agro-forests than intensive agriculture, while birds as a taxon have been found to be highly sensitive to forest conversion into agriculture based on multiple measures of biodiversity [2]. With approximately half of the closed-canopy forests in the tropics converted predominantly into agriculture [8], there is an ever-present need to understand the interaction of biodiversity with the matrix and its characteristics in important conservation landscapes [5,6].

The compositional elements of a matrix (e.g., habitat type, vegetation structure), and the spatial configuration of these elements (e.g., proximity to optimal/protected habitat) play an important role in structuring and determining the composition of biotic communities [6]. Previous research involving different taxonomic groups ranging from plants to mammals, reveal differential responses of various taxa to changes in composition and configuration of matrix elements [7,9]. For birds, the taxon of interest in this study, vegetation structure, in terms of its composition, plays a significant role in influencing community properties [10,11]. Vegetation structure mediates foraging substrates and resources for birds [12], and can also influence risk perception through providing cover [13]. Hence, the presence and abundance of different species is likely to be determined to a large degree by the kind of vegetation structure present. At the landscape scale, vegetation structure will invariably differ across the matrix, which in turn can significantly alter bird communities. In addition, spatial configuration of different matrix elements also impacts the way bird communities use the matrix. An important example is the proximity of matrix elements to optimal habitat: in many bird communities, increasing distance from contiguous forest habitat can greatly alter the structure and composition of the community [10].

A comprehensive approach to understanding the link between biotic communities and the matrix require the use of both taxonomic metrics as well as trait-based metrics to measure community response. Taxonomic metrics such as species richness and composition are highly useful in highlighting community changes relevant to conservation of important taxa. However, they fail to take into account species traits such as behavior, diet or ecosystem function/role that allow generalizations among compositionally different communities [14]. Trait-based metrics such as guild occupancy, on the other hand, address shared characters between species that allow for a mechanistic understanding of how environmental change might affect community structure disregarding compositional differences between communities [15,16]. However, these, in turn, fall short in their ability to highlight taxonomic changes that may be of conservation concern. Thus, the use of both types of metrics is highly important for studies relevant to biodiversity conservation and management to provide a localized as well as a general understanding of community response to land-use change.

In this study, we first use a guild-based framework to investigate how vegetation structure and proximity to a PA influence the wintering bird community that uses wooded land-use types surrounding a contiguous tropical forest in Meghalaya, Northeast India. We use an occupancy modeling approach to assess vegetation and proximity effects on guilds as it allows the separation of ecological and sampling processes [17]. In doing so, we also compare the site-use of individual guilds inside the PA to that in the surrounding matrix, as an evaluation of the ability of the wooded matrix to maintain community functionality. Second, we compare estimated species richness, and naïve site-use of detected species, between the PA and the surrounding wooded matrix to assess changes in taxonomic composition. We define wooded land-use types here as any land-cover type comprising of closed-canopy or open-canopy forests situated in the landscape, including, but not restricted to, natural forests, agroforests and mixed plantations. We conclude with a discussion on the possible mechanisms linking different guilds to vegetation structure and proximity to a PA, the processes that might explain site-use across the landscape by guilds, the relevance of taxonomic changes and the conservation implications of our study.

## Materials and methods

### Ethics statement

The Forest and Environment Department, Government of Meghalaya, India granted us research permits to conduct the study within government PAs. We obtained the consent of various village administrative bodies for fieldwork in village lands.

### Study area

**Fig 1:**
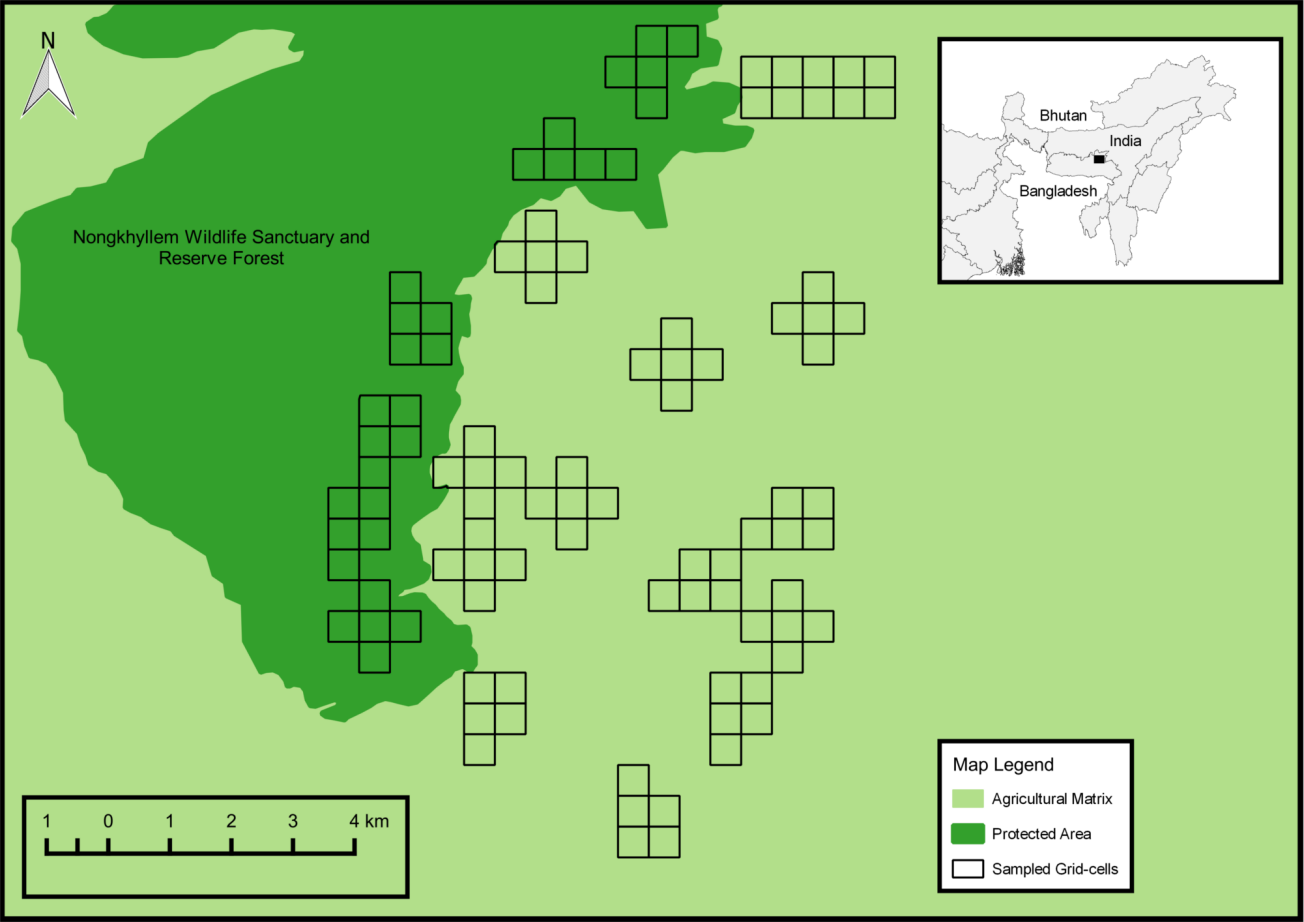
Map of study area in the Nongkhyllem Landscape, Meghalaya, India, showing the sampled sites (grid-cells) within the PA and the matrix. The top right inset shows the location of the study area in India (black rectangle).

We conducted our study in the Nongkhyllem landscape (25°45’–25°52’N, 91°44’–91°52’E) located in Meghalaya, Northeast India (Fig 1). The study area covered approximately 100km^2^ with an elevation range of 300–1000m above mean sea level. The landscape included the Nongkhyllem Wildlife Sanctuary and Reserve Forest, together comprising the Protected Area (hereafter, the PA), and a heterogeneous matrix of agricultural lands, community-managed forests, agro-forests and human habitation. The PA is a contiguous stand of relatively intact forest. However, the forest was subjected to some amount of selective logging approximately 20 years prior to the study.

### Bird surveys

We conducted bird surveys between November 2015 and May 2016, from the onset of winter to early summer to minimize overlap with the breeding and migratory season. We used a grid-based sampling design, which allowed us to attain spatial coverage of the entire study area. Each sampling unit was a grid-cell of 500m×500m, chosen to minimize within-cell heterogeneity in vegetation structure while maintaining independence of sampling points across grid-cells. We used the geographic information system (GIS) software QGIS 2.6.0 [18] to overlay the grid on the study area, as well as for all other GIS-related analyses. We excluded grid-cells that were completely composed of human habitation. In total, we surveyed 100 grid-cells covering the landscape.

We surveyed birds using 10-min, 100-m fixed-radius point counts. We randomly located five sampling locations per grid cell. We completed five point counts per day, one in each of five adjacent grid-cells for logistic convenience. In each grid-cell, we sampled the five chosen point count locations over five consecutive sampling days. Point counts located in completely open land-use types were shifted to the nearest wooded location since our inferences related to only wooded land-use types. Each sampling point was located not less than 100m from the grid-cell boundaries to ensure that birds detected at point counts could be clearly established as those present within that particular grid-cell. Within a grid-cell point counts were separated by an average distance of 149.34 m ± 71.30 m (mean ± sd). We used the point count method, as it is well suited for the detection of both canopy and understory birds, and is a logistically efficient method to sample across a large and heterogeneous landscape [19]. All bird species seen or heard during the counts were recorded. Birds that we could not identify to the species level during the counts, but whose guild identity we could unambiguously establish, were recorded for guild-level analyses.

### Covariate sampling

At each sampling point, canopy cover and bamboo cover were sampled using a GRS Densitometer^TM^ (Geographic Resource Solutions, Arcata CA, USA) at 22 points spaced 5m apart, located at and around the sampling point. Shrub cover was measured as shrub height using a vertically marked pole at 8 points, spaced 5m apart, around the sampling point (Fig A in S1 File). Trees that were >5m in height within a 10m × 10m plot around the sampling point were sampled for girth at breast height (GBH), which was then used to compute stand basal area. The Euclidean distance of each grid-cell centroid from the PA was extracted using QGIS 2.6.0.

### Guild classification

While designing the study, we aimed to classify detected bird species into guilds based on (i) size; (ii) foraging strata; and (iii) diet and (iv) foraging method. Criterion (iv) was exclusive to insectivorous guilds as it involved behavior such as sallying and gleaning, applicable to only insectivorous birds. These criteria were chosen as they represent those ecological characteristics of a species that indicate a mechanistic link to vegetation structure through resource requirements/acquisition (all three criteria; [11,20]), and to proximity to PA through dispersal ability (criterion one; [21]). This classification scheme could be followed completely for insectivorous species; non-insectivorous species could only be classified according to a diet guild, as there was little size variation within these guilds, and there was ambiguity in the foraging strata used by such species. Amongst the insectivorous species, we classified woodpeckers separately due to their specialized ecology. Small woodpeckers (including piculets), hornbills, shrikes, raptors and ground-dwelling birds of the family Phasianidae were detected very rarely. Hence, sample size was too low for guilds of these species to be included in the guild-level site-use analyses (described below). However, all species were included in the species level analyses, except for raptors and Phasianidae species as they required different sampling methods. We used information on different birds from Rasmussen and Anderton [22], Grimmett et al. [23] and Grimmett et al. [24] to classify them into guilds.

For analysis, we obtained a total of 11 guilds (for species classification into guilds see S5 File): four non-insectivorous guilds and seven insectivorous guilds. We included both forest specialists and generalists––even though some species were detected solely in the PA and some solely in the matrix––for inference made on maintenance of community functionality, which is determined by the presence of representative guilds (a starting point to understanding functional groups [25]) irrespective of their taxonomic composition.

### Assessing covariate effects and probability of site-use

We used the single-season occupancy model [17] to assess the influence of covariates on the probability of each guild using the landscape, and quantify probability of use inside the PA and the surrounding matrix. The occupancy modeling approach accounts for the imperfect detection of study animals, i.e., the probability of guilds being present within a grid-cell (hereafter, ‘site’), but not being recorded during surveys. Such non-detection has been shown to substantially impact both estimated probabilities of site-use, as well as species–or guild–habitat relationships [17]. Estimation of detection probability, or the probability of detecting a guild in a site, given its presence, is achieved through repeated sampling of sites; our sampling included 5 spatial replicates for each site.

The occupancy model is a hierarchical model comprising of two key parameters, for occupancy (Ψ) and detection (*p*) probabilities respectively, modeled as a function of ecological covariates. In this study, we estimate the probability of site-use (referred to only as ‘site-use’) rather than occupancy [26] as our site was smaller in area than bird home-ranges in general. Hence, our model estimates two parameters: (i) site-use (Ψ) and (ii) detection probability (*p*).

We selected covariates for site-use Ψ to represent composition and spatial configuration of a site. Composition was defined by vegetation structure––characterized by the covariates canopy cover, bamboo cover, stand basal area and shrub cover. Spatial configuration of each site was measured with respect to the PA, expressed as the distance of the site to the PA. We selected the covariates time from sunrise, canopy cover and shrub cover to model detection probability *p* as affected by both imperfect aural and visual detections. Covariates were standardized (mean of 0, standard deviation of 1) to improve convergence of models (see Table A and Table B in S1 File for untransformed values). Additive effects of these covariates were included after testing for correlations among covariates.

We used an information theoretic approach, the Akaike’s Information Criterion (AIC), for model selection [27]. This approach assesses the fit of a particular model to the observed data while penalizing for model complexity. For each guild, we first identified model structures that best describe variation in *p*. To do this, we ran different models for *p* (Table C in S1 File) while fixing Ψ to a general model (first model of Table D in S1 File). We then selected all covariate structures for *p* that corresponded to models where ΔAIC < 2 to run different models for Ψ (Table D in S1 File). Models that did not converge were removed from the model set. In general, models with ΔAIC < 2 were considered as those with sufficient support to make inference on the effects of covariates on sites-use of guilds. However, we clarify that we did not use this threshold in a very strict sense as we also examined models for uninformative parameters [28]; models with uninformative parameters were not included in the chosen model set.

The estimated covariate coefficients for each guild were not model-averaged across models [29]. We present estimated covariate coefficient values from the models in the chosen model set with the most parameterized covariance structure for site-use. Estimated covariate coefficients for detection probability are reported from the same models that are used to report site-use coefficients. If the spatially invariant model (null model) also figured in the chosen model set, other models in the chosen model set––and by extension, the covariate effects included in these models––were presented as having only weak support.

Guild site-use was quantified by model averaging the predictions across the model set [29]. We compared average estimated guild site-use for grid-cells that fell within the PA, to that in the surrounding matrix by calculating the mean of model-averaged predictions for site-use of PA grid-cells and matrix grid-cells separately. The occupancy analysis was carried out using the ‘unmarked’ package in the statistical software R version 3.3.0 [30,31]. Additional packages used were ‘ggplot2’, ‘gridExtra’ and ‘gtable [32–34].

### Estimating species richness within and outside the PA

To compare species richness within and outside the PA, we estimated species richness separately for sites that fell within, and those that fell outside the PA. Raw counts of detected species can be a misleading indicator of species richness as there are likely to be species that go undetected during surveys. Capture–recapture analyses successfully address this issue by accounting for heterogeneous detection probabilities across species [35]. For the purpose of this analysis, we considered each site as a replicate. We thus obtained a detection history for each species, indicating detections and non-detections in each site for the PA, and the matrix outside the PA. We used the program SPECRICH2 [36] for this analysis.

### Comparing naïve site-use of species within and outside the PA

We calculated naïve site-use, i.e., the proportion of sites within and outside the PA used by different species but uncorrected for detection probability. Calculation of this metric was based on detection/non-detection data of individual species obtained from the five sampling replicates per site described earlier. For each species at each site, detection histories obtained from replication was converted into a binary form for calculation of naïve site-use. Detection in at least one sampling replicate was taken as 1 and non-detection in any of the replicates was considered as 0.

## Results

Covariate effects on detection probability and site-use are presented for 11 guilds: nectarivores, granivores, omnivores, frugivores, large high-canopy gleaning insectivores, large understory gleaning insectivores, large high-canopy sallying insectivores, small mid-canopy gleaning insectivores, small understory gleaning insectivores, small mid-canopy sallying insectivores and large woodpeckers. The model selection table for each guild is given in S2 File.

### Effect of covariates on detection probabilities of guilds

Covariate effects on detection probability varied across guilds. In general, detection probability was higher in the early morning hours for seven guilds. Increase in canopy cover lowered the detection probability of two guilds, whereas it increased the detection probability of three guilds. Increase in shrub cover decreased the detection probability of two guilds, while it increased the detection probability of two guilds. There was weak support for a negative influence of shrub cover on the detection probability of frugivores. However, the spatially invariant model was also supported among the chosen models. Covariate effects on detection probability for the chosen model set of each guild are summarized in Table 1. Detailed coefficients for all models are given in S4 File.

**Table 1:** Covariate coefficients of detection probability for 11 guilds as estimated through single-season occupancy models.

Covariate	Non-insectivorous guilds				Insectivorous guilds						
	Nectarivore	Granivore	Omnivore	Frugivore	Large high-canopy gleaning insectivore	Large understory gleaning insectivore	Large high-canopy sallying insectivore	Small mid-canopy gleaning insectivore	Small understory gleaning insectivore	Small mid-canopy sallying insectivore	Large woodpecker
Time from sunrise	-	–0.43 (0.17)	-	-	–0.16 (0.11)	–0.37 (0.14)	–0.17 (0.10)	–0.37 (0.10)	–0.30 (0.12)	-	-0.30 (0.13)
Canopy cover	-	–0.20 (0.17)	-	-	-	-	0.35 (0.11)	-	–0.36 (0.14)	0.81 (0.14)	1.00 (0.14)
Shrub cover	-	-	–0.55 (0.18)	–0.15 (0.11)*	-	-	-	–0.10 (0.10)	0.31 (0.12)	0.40 (0.14)	-

Standard errors are shown in parentheses. Guilds where covariate effect is unsupported are marked with a dash (‘–‘).Guilds where covariate effect has weak support are marked with an asterisk (‘*’).

### Effect of covariates on site-use of guilds

Increasing canopy cover reduced the site-use of granivores (–0.36 ± 0.39) and omnivores (–0.70 ± 1.17). In addition, there was weak support for a negative impact of canopy cover on large high-canopy gleaning insectivores and small understory gleaning insectivores. However, for both guilds, the spatially invariant model was also among the chosen models. Bamboo cover had a negative effect on granivores (–0.85 ± 0.43) and omnivores (–0.65 ± 0.47) and a positive effect on small mid-canopy sallying insectivores (3.41 ± 3.33).

There was no strong support that stand basal area affected site-use of any guild. However, there was weak support for a negative impact of this covariate on small understory gleaning insectivores, with the spatially invariant model also amongst the chosen models.

Shrub cover was supported in the top model set of three guilds: nectarivores, granivores, large high-canopy sallying insectivores. Shrub cover benefitted nectarivores (3.00 ± 1.90) and granivores (0.42 ± 0.39) but was detrimental to large high-canopy sallying insectivores (–1.09 ± 0.37) and small mid-canopy sallying insectivores (-0.63 ± 0.37). There was also weak support for shrub cover positively affecting large woodpeckers.

Distance from PA had no strong support in the chosen model set of any guild, but had weak support for a positive effect on large high-canopy gleaning insectivores and small mid-canopy gleaning insectivores.

The spatially invariant model for site-use was the best supported model for two guilds: frugivores and large understory gleaning insectivores. The frugivore guild initially had a naïve site-use of 0.99; hence most models with covariates did not converge. The non-convergence was driven in part by the most abundant species, the black-hooded oriole *Oriolus xanthornus*, which comprised 23.45% of detections in this guild (Fig B in S1 File). However, even after the removal of this species naïve site-use only reduced to 0.98 due to the presence of other abundant species. The models converged after the removal of this species, and in accordance with the high naïve site-use, the spatially invariant model was the best supported model for site-use of this guild.

**Fig 2:**
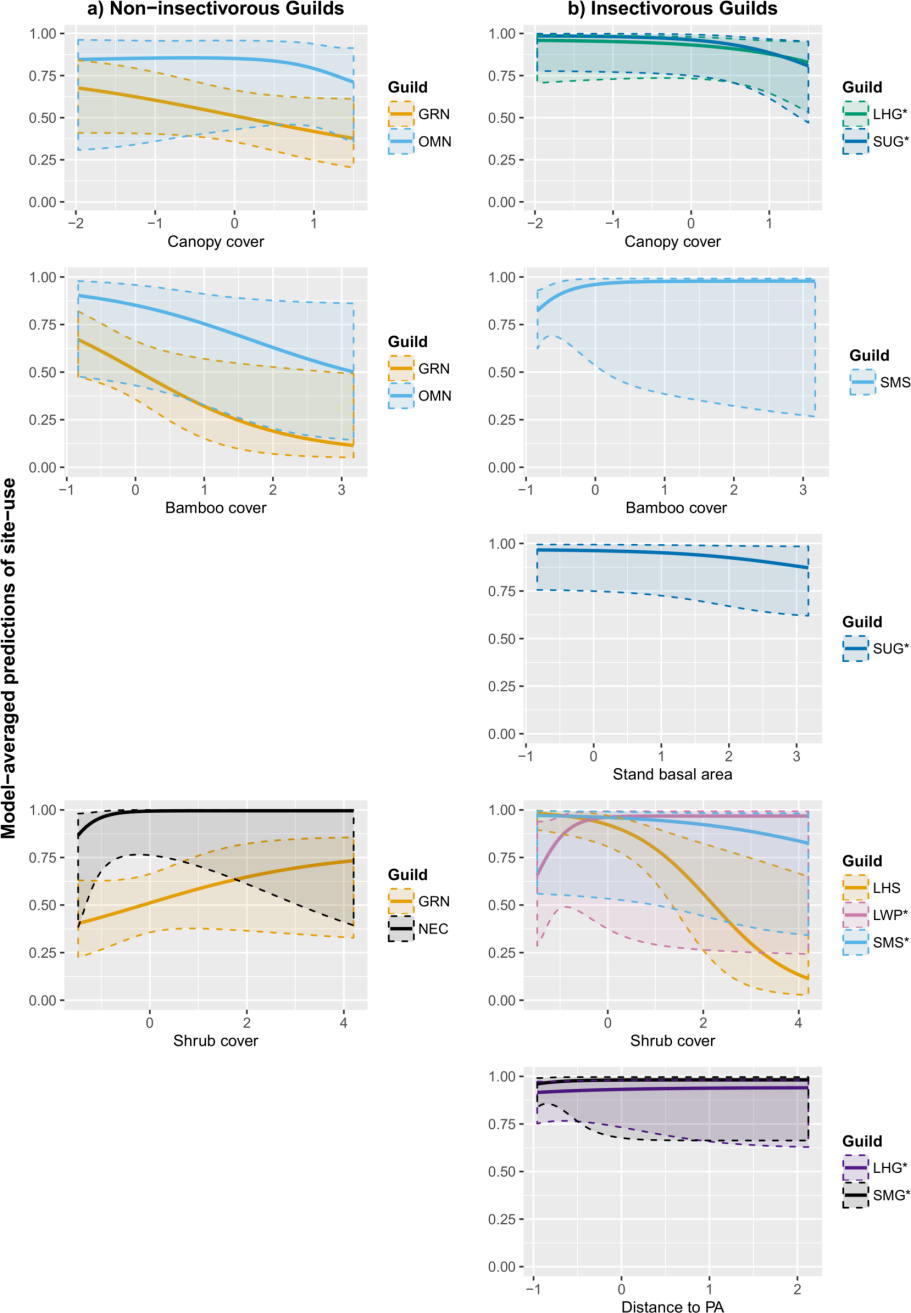
**Predicted site-use of a) non-insectivorous guilds and b) insectivorous guilds as a function of vegetation and proximity effects as estimated through single-season occupancy models.** Predictions were model-averaged across all converged models; shaded regions represent associated standard errors. Covariates were standardized to have a mean of 0 and standard deviation of 1. Guilds where covariate effect has weak support are marked with an asterisk (‘*’). Guilds where covariate effect is unsupported are not included. Guild abbreviations: NEC (Nectarivores), GRN (Granivores), OMN (Omnivores), FRG (Frugivores), LHG (Large high-canopy gleaning insectivores), LUG (Large understory gleaning insectivores), LHS (Large high-canopy sallying insectivores), SMG (Small mid-canopy gleaning insectivores), SUG (Small understory gleaning insectivores), SMS (Small mid-canopy sallying insectivores), LWP (Large woodpeckers).

Covariate effects on site-use for each guild are summarized in Table 2. Fig 2 shows the effects of individual covariates on model-averaged predictions of site-use for each guild. Detailed coefficients for all models are given in S3 File.

**Table 2:** Covariate coefficients of site-use for 11 guilds as estimated through single-season occupancy models.

Covariate	Non-insectivorous guilds				Insectivorous guilds						
	Nectarivore	Granivore	Omnivore	Frugivore	Large high-canopy gleaning insectivore	Large understory gleaning insectivore	Large high-canopy sallying insectivore	Small mid-canopy gleaning insectivore	Small understory gleaning insectivore	Small mid-canopy sallying insectivore	Large woodpecker
Canopy Cover	-	–0.36 (0.39)	–0.70 (1.17)	-	–0.77 (0.53)*	-	-	-	–1.51 (0.71)*	-	-
Bamboo Cover	-	–0.85 (0.43)	–0.65 (0.47)	-	-	-	-	-	-	3.41 (3.33)	-
Stand Basal Area	-	-	-	-	-	-	-	-	–1.09 (0.60)*	-	-
Shrub Cover	3.00 (1.90)	0.42 (0.39)	-	-	-	-	–1.09 (0.37)	-	-	-0.63 (0.37)	3.53 (3.03)*
Distance from PA	-	-	-	-	1.12 (1.46)*	-	-	3.50 (5.59)*	-	-	-

Standard errors are shown in parentheses. Guilds where covariate effect is unsupported are marked with a dash (‘–‘). Guilds where covariate effect has weak support are marked with an asterisk (‘*’).

### Site-use of guilds inside and outside PA

**Fig 3:**
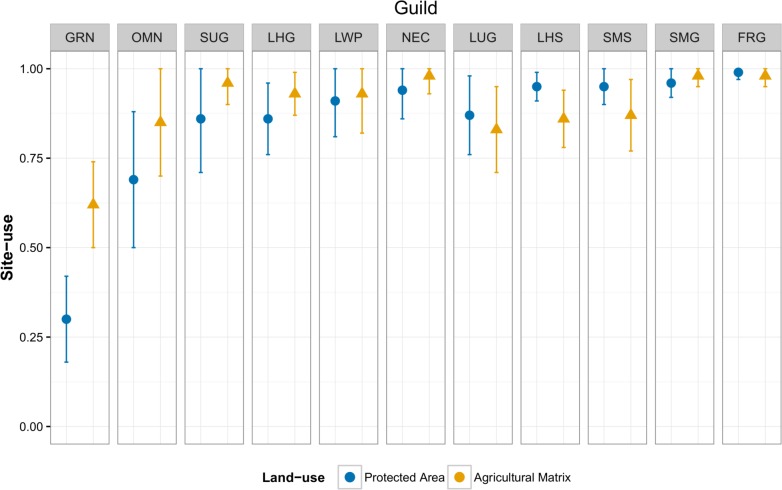
Mean site-use of guilds in the PA and in the matrix. Error bars represent standard errors. Guilds shown towards the left show higher estimated site-use in the matrix, while those towards the right show higher estimated site-use in the PA. Guild abbreviations: GRN (Granivores), OMN (Omnivores), SUG (Small understory gleaning insectivores), LHG (Large high-canopy gleaning insectivores), LWP (Large woodpeckers), NEC (Nectarivores), LUG (Large understory gleaning insectivores), LHS (Large high-canopy sallying insectivores), SMS (Small mid-canopy sallying insectivores), SMG (Small mid-canopy gleaning insectivores), FRG (Frugivores).

Mean site-use was very high (>0.80) for most guilds both within the PA and in the matrix. Exceptions to this were the granivores and omnivores which had lower mean site-use in the PA as compared to the matrix. Mean site-use for granivores in the PA was 0.30 ± 0.12 (mean ± SE) as compared to 0.62 ± 0.12 in the matrix; mean site-use for omnivores in the PA was 0.69 ± 0.19, and in the matrix was 0.85 ± 0.15. Mean site-use for the all guilds is visualized in Fig 3. See Table E in S1 File for detailed values.

### Avian community species richness in the landscape

**Fig 4:**
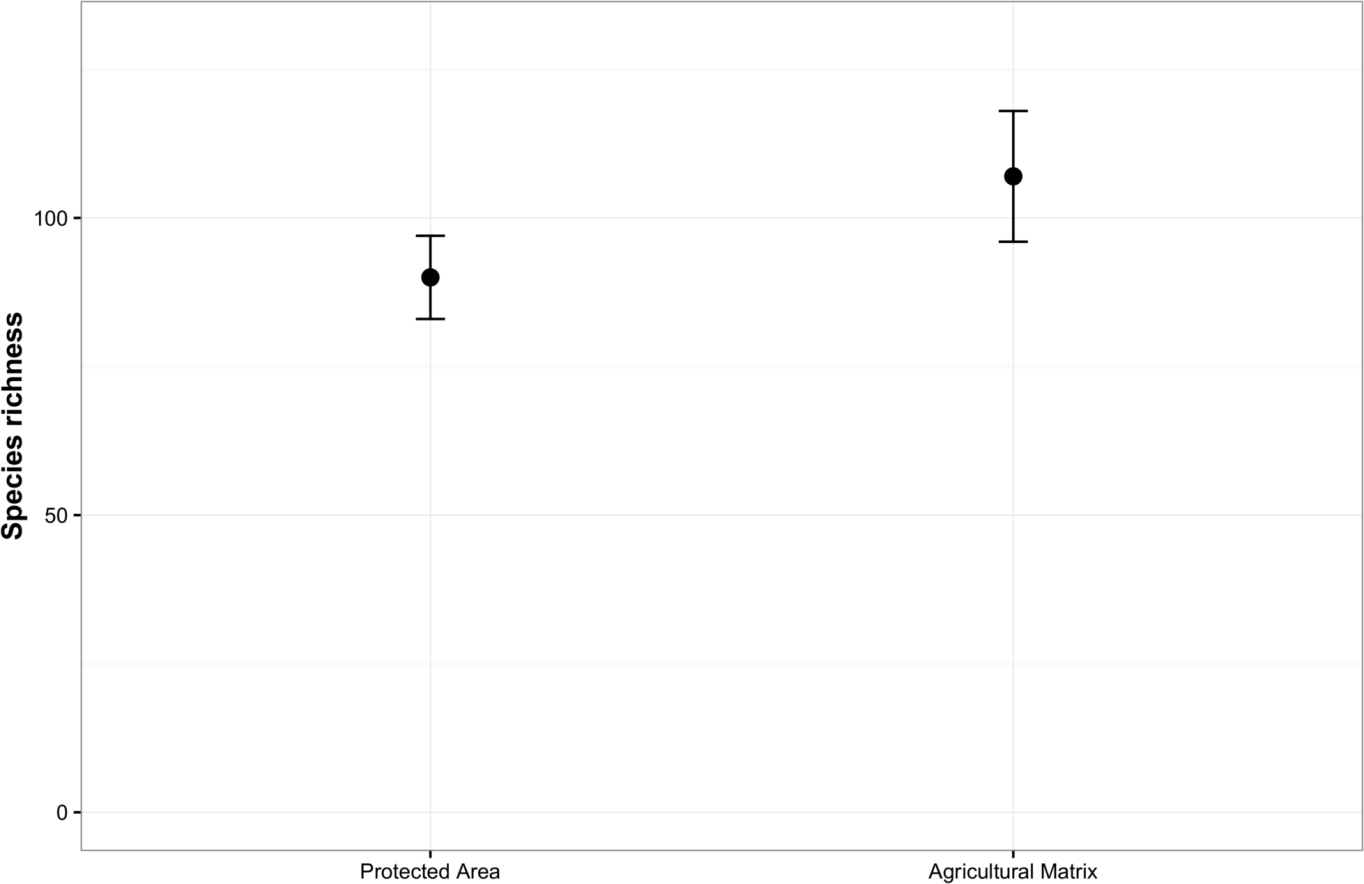
Estimates of species richness for sites located in the PA and in the matrix in the study area at Meghalaya. Estimates were obtained from capture–recapture sampling of species that accounted for heterogeneous detection probabilities across species.

In total, we detected 94 species across 100 sites during the entire sampling period. Of these, 11 species were exclusive to sites within the PA, 20 species were exclusive to sites in the matrix outside the PA, and 63 species were detected both within the PA and the matrix (For list of species see Table G in S1 File). PA sites had 74 detected species. The matrix had 83 detected species. Estimates of species richness was 90 ± 7(mean ± SE) species for the PA and 107± 11(mean ± SE) for the matrix (Fig 4).

### Naïve site-use by species inside and outside the PA

**Fig 5:**
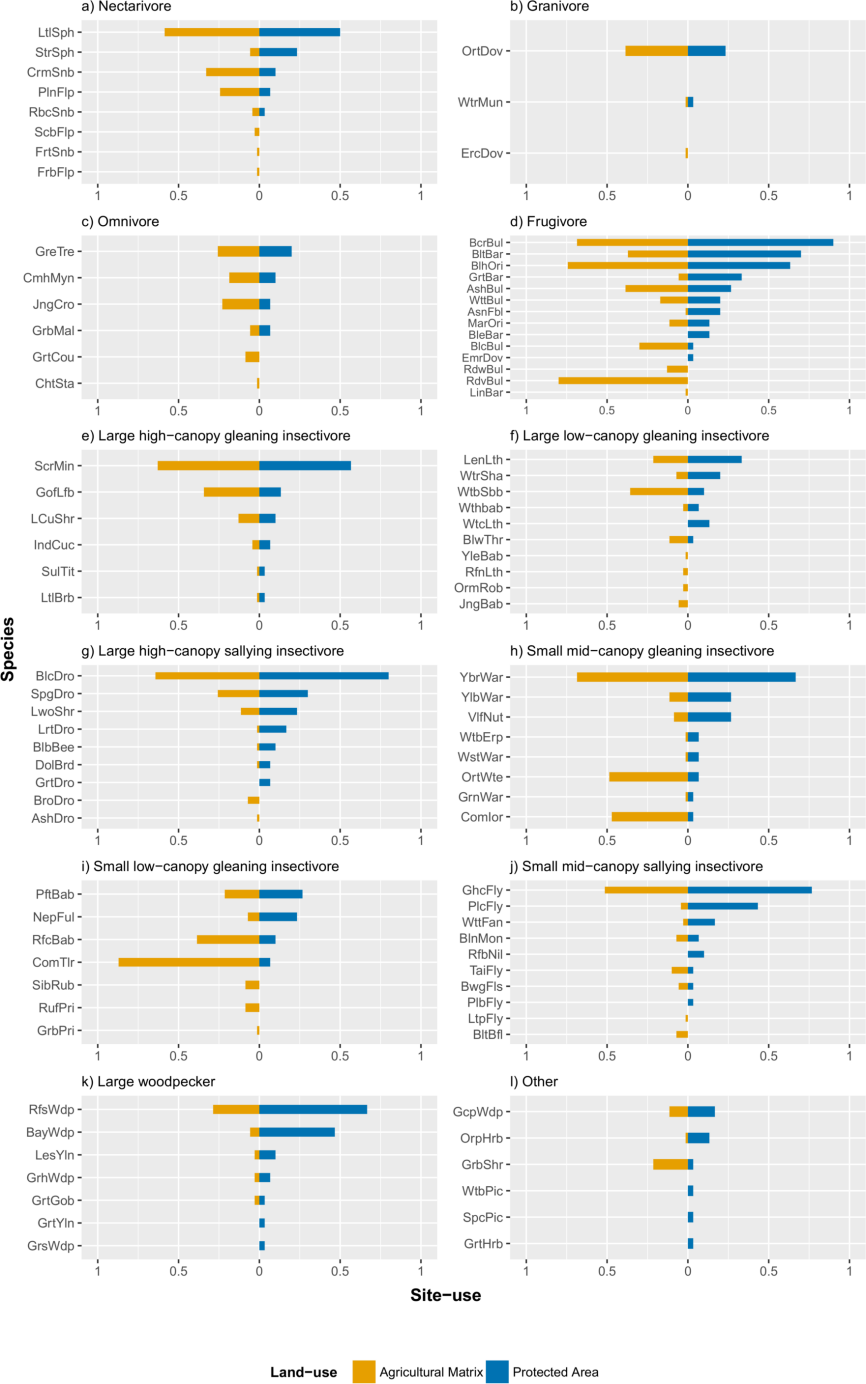
Comparison of naïve site-use by species in the matrix and in the PA. Estimates of site-use were based on the proportion of sites in the matrix and the PA where a species was detected. Expanded species abbreviations are given in Table F in S1 File.

Of the 94 species detected during our study, 49 species had a higher naïve site-use in the PA than in the matrix while 45 species had a higher naïve site-use in the matrix than in the PA. The magnitude of naïve site-use change between PA and the matrix for each species is visualized in Fig 5 (see also Table F in S1 File).

## Discussion

Our study brings to light two interesting findings. First, in our assessment of vegetation and proximity effects on the site-use of various guilds of the wintering bird community in wooded land-use types of the matrix, we found that tree cover variables were not the limiting factors for most guilds. Interestingly though, shrub cover and bamboo cover were found to be important factors for some of the guilds that were not limited by tree cover. Second, our study highlights the ability of wooded land-use types in the matrix to maintain high-levels of site-use by multiple guilds, and hence the functionality, of the wintering community. However, the maintenance of community functionality did not correspond with the maintenance of community composition.

### Canopy cover not the limiting factor for guild site-use in wooded land-use types

Our finding that canopy cover was not a factor limiting site-use for the majority of the guilds is consistent with our study focus on wooded land-use types, where tree cover variables are expected to have higher values. The overall canopy cover in all but three of our sites was over 20%, which may have been adequate to maintain site-use by these guilds (see [37]). Supporting this, we also found that all guilds not limited by canopy cover were woodland-associated guilds, except for small understory gleaning insectivores. Moreover, the guilds where canopy cover had a strong negative effect were guilds containing generalist species (granivores and omnivores).This finding in our study is further reflected on the high estimated site-use by these guilds across the landscape, which we discuss in more detail in the sections below.

Notwithstanding the lack of effect of tree cover variables, the effects of shrub cover and bamboo cover on two woodland-associated guilds suggests that other components of vegetation structure are also important in governing site-use in wooded land-use types. Miller and Cale [38] also found shrub density to be an important factor predicting the number of foraging guilds in woodland patches in Australia. However, Raman et al. [39], found that bamboo affected mainly non-forest bird species in India. Many related studies account for different components of vegetation structure by combining them with other variables into one or two integrated vegetation axes (e.g., [39]). While this analytical method is highly useful and necessary in many cases, we feel that separately testing for different components of vegetation structure, whenever feasible, can also help capture nuanced relationships that may otherwise go unexplained. Moreover, it can also help us understand the processes operating when those components become limiting factors. For example, when tree cover is not limiting, the negative effect of shrub cover on large high-canopy sallying insectivores, and small mid-canopy sallying insectivores, help point to several different hypotheses: spatial restrictions on foraging method imposed by shrub cover [20], or variations in insect prey abundance/diversity [40] or access to prey [41] with changing shrub cover, to suggest a few. Separately testing for different components of vegetation structure becomes especially important when we consider the fact that shrub cover and bamboo cover can undergo dramatic change with human-use even when tree cover is left intact.

While we accounted for the effects of the different components of vegetation structure in our current study, we further suggest that testing the floristic effects of vegetation, and their associated resources would be an important second step [42] to better our understanding of the mechanistic relationship of vegetation with guilds of the bird community.

The weak support for the effects of proximity to PA in our models suggest that site-use by guilds are not limited with increasing distance from the PA. However, differences in species naïve site-use between the PA and the matrix might suggest that proximity to PA is more important a factor when it is considered at the species level.

### Maintenance of guilds across the landscape

Our study found a high use of the landscape by all analyzed guilds both inside the PA, and in wooded land-use types of the matrix outside the PA. This suggests that the matrix surrounding the PA in our study area was capable of supporting site-use by multiple guilds of the wintering bird community. Since our focus was on wooded land-use types only, we add that our inference does not extend to open land-use types such as paddy fields and human habitation.

Three explanations appear to work together to maintain the high site-use of the matrix by the analyzed guilds in our study area. 1) We found that within seven guilds, there were ubiquitous species that had relatively high site-use in both the matrix and the PA. Species such as the little spider hunter *Arachnothera longistra*, black-crested bulbul *Pycnonotus flaviventris*, black-hooded oriole *Oriolus xanthornus*, scarlet minivet *Pericrocotus speciosus*, black drongo *Dicrurus macrocercus*, yellow-browed warbler *Phylloscopus inornatus* and grey-headed canary flycatcher *Culicicapa ceylonensis* had relatively high site-use in both the matrix and the PA. 2) There was some level of redundancy of species within guilds. This buffered against a decrease in guild site-use due to loss of species and/or decrease in site-use as the habitat changes from the PA to the matrix. For example, within the large woodpecker guild, although all species decreased in site-use from the PA to the matrix––with two species, greater yellownape *Picus flavinucha* and great slaty woodpecker *Mulleripicus pulverulentus*, completely disappearing––guild site-use was still comparable between the PA and the matrix. 3) A turnover in guild composition and/or abundances to more generalist species as well as addition of new species in the matrix was found in a few guilds. For example, within the frugivore and small low-canopy gleaning insectivore, there was a noticeable site-use increase in the matrix, as compared to the PA, of the red-vented bulbul *Pycnonotus cafer* and common tailorbird *Orthotomus sutorius* respectively; these species are known generalists. Similarly, within the small mid-canopy gleaning insectivore guild, there was a noticeable site-use decrease in two species–yellow-bellied warbler *Abroscopus superciliaris* and velvet-fronted nuthatch *Sitta frontalis*, with an increase in two other species–oriental white-eye *Zosterops palpebrosus* and common iora *Aegithina tiphia* in the matrix. The comparable site-use by guilds between the matrix and the PA could also suggest that movement of species belonging to those guilds was possible through the matrix, i.e., the sampled wooded land-use types were either structurally or functionally connected, even those furthest from the PA. Such movement has been found to occur in other tropical agricultural matrices; the movement of birds either depended on the intervening land-use type [43] or on traits of the birds–forest-specialist versus generalist [44]. The movement of species through the matrix could also operate in conjunction with the turnover in guild composition and abundances, whereby generalist species are better adapted to move through more open land-use types.

An important point to note in our study is that although we only looked at site-use by guilds and not occupancy, and hence cannot ascertain which areas contain resident bird populations, it makes ecological sense to reason that the high site-use by guilds especially far away from the PA is due to populations that are resident within the matrix itself.

### Taxonomic changes across the landscape

Although we found that the species richness was higher in the matrix than in the PA, many of the additional species found in the matrix were also generalist species, with a few exceptions. For example, red-vented bulbul, oriental magpie robin *Copsychus saularis*, jungle babbler *Turdoides striata*, Siberian rubythroat *Luscinia calliope*, rufescent prinia *Prinia rufescens* and grey-breasted prinia *Prinia hodgsonii*, all of them generalists, were additional detections in the matrix. However, the great hornbill *Buceros bicornis* a large seed-dispersing bird of high conservation value was only encountered within the PA. In addition, two large woodpecker species–the greater yellownape and great slaty woodpecker, and two piculet species–white-browed piculet *Sasia ochracea* and speckled piculet *Picumnus innominatus*, were also detected only within the PA. This suggests that while the matrix can support higher species richness, due to the addition of generalist species, some specialized species such as hornbills, woodpeckers and piculets may lose out.

### Guild-specific detection probability

Our study found that detection probability varied between guilds both spatially and temporally. Except for nectarivores, our site-use estimates for all guilds would have been biased if we had not accounted for factors such as time, canopy cover and shrub cover that could affect our ability to see or hear birds during sampling. These findings point to the importance of accounting for detection probability in ecological studies of birds in order for a more robust inference. For this same reason, we state that our interpretation of species level site-use is a naïve estimate in the sense that it is uncorrected for detection probability, and only provides speculations to possible ecological trends.

### Conservation implications

Forest conversion due to the expansion of the matrix seems inevitable under current trends of land-use change, often with negative impacts on the composition, structure and function of the resident biotic communities. The need to better manage the matrix in order to mitigate these impacts becomes ever increasing as more of the world’s biodiversity gets lost with each passing day. Our study highlights the value of wooded land-use types in the matrix surrounding forests for maintaining the functionality of a wintering bird community. In the context of our study landscape, i.e., the Ri-Bhoi District of Meghalaya, Northeast India, we recommend land managers to promote the prevailing wooded land-use types such as recovering secondary forests, community-managed forests and betel leaf cultivation forests due to their high predicted value for supporting multiple bird guilds. We expect that a decrease in such wooded land-use types across the landscape will disrupt the retention of multiple bird guilds. Although, there is a lack of scientific information on land-use change in the region, there has been an observable increase in more open land-use types––such as broom-grass *Thysanolaena maxima* plantations––over the past decade, which may be a cause of concern. In other parts of India as well, the conservation value of agricultural matrices for birds has been shown to be enhanced by the presence of wooded land-use types such as shade-coffee plantations, rubber plantations and other agro-forests [45]. However, we caution that prevailing wooded land-use types in the matrix may not be enough to retain certain species that appear to be found, or have a high naïve site-use, only in the PA. Species such as hornbills, large woodpeckers and piculets may need a management approach similar to that of the PA to have continued presence in the matrix. Nonetheless, the agricultural matrix has a conservation role to play in human-dominated heterogeneous landscapes; our study provides scientific findings on community functionality and individual species distribution in the matrix that can inform landscape-scale planning to better enhance the conservation value of these lands.

## Supporting information

S1 File Supporting information—Tables and figures. (DOCX)

S2 File Model selection of site-use and detection probability for different guilds using an occupancy-based approach. Model selection was conducted using AIC, while removing all uninformative models. Chosen models for inference are highlighted in bold.
(DOCX)

S3 File Covariate coefficients of site-use for all the models of each guild. Standard errors are shown in parentheses. Model selection was conducted using AIC, while removing all uninformative models. Chosen models for inference are highlighted in bold. Covariate coefficients for site-use are reported from models marked ‘*’.
(DOCX)

S4 File Covariate coefficients of detection probability for all the models of each guild. Standard errors are shown in parentheses. Model selection was conducted using AIC, while removing all uninformative models. Chosen models for inference are highlighted in bold. Covariate coefficients for detection probability are reported from models marked ‘*’.
(DOCX)

S5 File List of bird species recorded during the study period, along with their assigned guilds. Nomenclature follows Grimmett *et al*. (2011).
(DOCX)

S6 File Species detection–non-detection data and covariate data used in our study. Separate worksheets contain: i) Species detection–non-detection data, ii) Site covariate data and iii) Sampling occasion covariate data for time (minutes) from sunrise.
(XLSX)
