# Supplementary material for: “In a tree by the brook, there’s a songbird who sings”: Woodlands in an agricultural matrix maintain functionality of a wintering bird community
Source: PLoS One. 2018 Aug 2;13(8):e0201657. doi: 10.1371/journal.pone.0201657 (PMC6072076; doi:10.1371/journal.pone.0201657)
Supplement: S1 File — (DOCX) [file pone.0201657.s001.docx]

**S1 File. Supporting information - tables and figures.**

**Table A.** Mean and standard deviation of covariates for site-use

| **Ψ** |  |  |
| --- | --- | --- |
| **Covariate** | **Mean** | **SD** |
| Canopy cover (%) | 62.28 | 25.14 |
| Bamboo cover (%) | 20.35 | 24.38 |
| Stand basal area (basal area/sq. m) | 23.48 | 16.79 |
| Shrub cover (m) | 0.71 | 0.48 |
| Distance to PA (m) | 1733.15 | 1809.09 |

**Table B.** Mean and standard deviation of covariates for detection probability

| ***P*** |  |  |
| --- | --- | --- |
| **Covariate** | **Mean** | **SD** |
| Time from sunrise (min from sunrise) | 104.31 | 61.02 |
| Canopy cover (%) | 62.28 | 25.14 |
| Shrub cover (m) | 0.72 | 0.48 |

**Table C.** Model set with different covariate combinations for *p*

| **Covariate combination** |
| --- |
| *p*(time from sunrise + canopy cover + shrub cover) |
| *p*(time from sunrise + canopy cover) |
| *p*(time from sunrise + shrub cover) |
| *p*(canopy cover + shrub cover) |
| *p*(time from sunrise) |
| *p*(canopy cover) |
| *p*(shrub cover) |
| *p*(.) |

**Table D.** Model set with different covariate combinations for Ψ.

| **Covariate combination** |
| --- |
| Ψ(canopy cover + bamboo cover + shrub cover + distance from PA) |
| Ψ(canopy cover + bamboo cover + shrub cover) |
| Ψ(canopy cover + bamboo cover) |
| Ψ(canopy cover + shrub cover) |
| Ψ(bamboo cover + shrub cover) |
| Ψ(stand basal area+ shrub cover) |
| Ψ(canopy cover) |
| Ψ(bamboo cover) |
| Ψ(stand basal area) |
| Ψ(shrub cover) |
| Ψ(distance from PA) |
| Ψ(.) |

**Table E.** Mean site-use estimates of guilds inside the protected area and in the agricultural matrix. Standard errors are shown in parentheses.

| **Guild** | **ψ _Protected Area_** | **ψ _Agricultural Matrix_** |
| --- | --- | --- |
| Nectarivores | 0.94 (0.08) | 0.98 (0.05) |
| Granivores | 0.30 (0.12) | 0.62 (0.12) |
| Omnivores | 0.69 (0.19) | 0.85 (0.15) |
| Frugivores | 0.99 (0.02) | 0.98 (0.03) |
| Large high-canopy gleaning insectivores | 0.86 (0.10) | 0.93 (0.06) |
| Large understory gleaning insectivores | 0.87 (0.11) | 0.83 (0.12) |
| Large high-canopy sallying insectivores | 0.95 (0.04) | 0.86 (0.08) |
| Small mid-canopy gleaning insectivores | 0.96 (0.04) | 0.98 (0.03) |
| Small understory gleanning insectivores | 0.86 (0.15) | 0.96 (0.06) |
| Small mid-canopy sallying insectivores | 0.95 (0.05) | 0.87 (0.10) |
| Large woodpeckers | 0.91 (0.10) | 0.93 (0.11) |

**Table F.** Naïve site-use of different species, i.e., the proportion of sites used but uncorrected for detection probability, in the PA and in the agricultural matrix

| **Guild** | **Species** | **Abbreviation** | **Naïve site-use in PA** | **Naïve site-use in Agricultural matrx** |
| --- | --- | --- | --- | --- |
| Nectarivore | Little Spiderhunter | LtlSph | 0.50 | 0.59 |
| Nectarivore | Streaked Spiderhunter | StrSph | 0.23 | 0.06 |
| Nectarivore | Crimson Sunbird | CrmSnb | 0.10 | 0.33 |
| Nectarivore | Plain Flowerpecker | PlnFlp | 0.07 | 0.24 |
| Nectarivore | Ruby-cheeked Sunbird | RbcSnb | 0.03 | 0.04 |
| Nectarivore | Scarlet-backed Flowerpecker | ScbFlp | 0.00 | 0.03 |
| Nectarivore | Fire-tailed Sunbird | FrtSnb | 0.00 | 0.01 |
| Nectarivore | Fire-breasted Flowerpecker | FrbFlp | 0.00 | 0.01 |
| Granivore | Oriental Turtle Dove | OrtDov | 0.23 | 0.39 |
| Granivore | White-rumped Munia | WtrMun | 0.03 | 0.01 |
| Granivore | Eurasian Collared Dove | ErcDov | 0.00 | 0.01 |
| Omnivore | Grey Treepie | GreTre | 0.20 | 0.26 |
| Omnivore | Common Hill Myna | CmhMyn | 0.10 | 0.19 |
| Omnivore | Green-billed Malkoha | GrbMal | 0.07 | 0.06 |
| Omnivore | Eastern Jungle Crow | JngCro | 0.07 | 0.23 |
| Omnivore | Greater coucal | GrtCou | 0.00 | 0.09 |
| Omnivore | Chestnut-tailed Starling | ChtSta | 0.00 | 0.01 |
| Frugivore | Black-crested Bulbul | BcrBul | 0.90 | 0.69 |
| Frugivore | Blue-throated Barbet | BltBar | 0.70 | 0.37 |
| Frugivore | Black-hooded Oriole | BlhOri | 0.63 | 0.74 |
| Frugivore | Great Barbet | GrtBar | 0.33 | 0.06 |
| Frugivore | Ashy Bulbul | AshBul | 0.27 | 0.39 |
| Frugivore | White-throated Bulbul | WttBul | 0.20 | 0.17 |
| Frugivore | Asian Fairy Bluebird | AsnFbl | 0.20 | 0.01 |
| Frugivore | Maroon Oriole | MarOri | 0.13 | 0.11 |
| Frugivore | Blue-eared Barbet | BleBar | 0.13 | 0.00 |
| Frugivore | Black Bulbul | BlcBul | 0.03 | 0.30 |
| Frugivore | Emerald Dove | EmrDov | 0.03 | 0.00 |
| Frugivore | Red-whiskered Bulbul | RdwBul | 0.00 | 0.13 |
| Frugivore | Red-vented Bulbul | RdvBul | 0.00 | 0.80 |
| Frugivore | Lineated Barbet | LinBar | 0.00 | 0.01 |
| Large high-canopy gleaning insectivore | Scarlet Minivet | ScrMin | 0.57 | 0.63 |
| Large high-canopy gleaning insectivore | Golden-fronted Leafbird | GofLfb | 0.13 | 0.34 |
| Large high-canopy gleaning insectivore | Large Cuckooshrike | LCuShr | 0.10 | 0.13 |
| Large high-canopy gleaning insectivore | Indian Cuckoo | IndCuc | 0.07 | 0.04 |
| Large high-canopy gleaning insectivore | Sultan Tit | SulTit | 0.03 | 0.01 |
| Large high-canopy gleaning insectivore | Long-tailed Broadbill | LtlBrb | 0.03 | 0.01 |
| Large low-canopy gleaning insectivore | Lesser Necklaced Laughingthrush | LenLth | 0.33 | 0.21 |
| Large low-canopy gleaning insectivore | White-rumped Shama | WtrSha | 0.20 | 0.07 |
| Large low-canopy gleaning insectivore | White-browed Scimitar Babbler | WtbSbb | 0.10 | 0.36 |
| Large low-canopy gleaning insectivore | White-hooded Babbler | Wthbab | 0.07 | 0.03 |
| Large low-canopy gleaning insectivore | White-crested Laughingthrush | WtcLth | 0.13 | 0.00 |
| Large low-canopy gleaning insectivore | Blue Whistling Thrush | BlwThr | 0.03 | 0.11 |
| Large low-canopy gleaning insectivore | Yellow-eyed Babbler | YleBab | 0.00 | 0.01 |
| Large low-canopy gleaning insectivore | Rufous-necked Laughingthrush | RfnLth | 0.00 | 0.03 |
| Large low-canopy gleaning insectivore | Oriental Magpie Robin | OrmRob | 0.00 | 0.03 |
| Large low-canopy gleaning insectivore | Jungle Babbler | JngBab | 0.00 | 0.06 |
| Large high-canopy sallying insectivore | Black Drongo | BlcDro | 0.80 | 0.64 |
| Large high-canopy sallying insectivore | Spangled Drongo | SpgDro | 0.30 | 0.26 |
| Large high-canopy sallying insectivore | Large Woodshrike | LwoShr | 0.23 | 0.11 |
| Large high-canopy sallying insectivore | Lesser Racket-tailed Drongo | LrtDro | 0.17 | 0.01 |
| Large high-canopy sallying insectivore | Blue-bearded Bee-eater | BlbBee | 0.10 | 0.01 |
| Large high-canopy sallying insectivore | Dollarbird | DolBrd | 0.07 | 0.01 |
| Large high-canopy sallying insectivore | Greater Racket-tailed Drongo | GrtDro | 0.07 | 0.00 |
| Large high-canopy sallying insectivore | Bronzed Drongo | BroDro | 0.00 | 0.07 |
| Large high-canopy sallying insectivore | Ashy Drongo | AshDro | 0.00 | 0.01 |
| Small mid-canopy gleaning insectivore | Yellow-browed Warbler | YbrWar | 0.67 | 0.69 |
| Small mid-canopy gleaning insectivore | Yellow-bellied Warbler | YlbWar | 0.27 | 0.11 |
| Small mid-canopy gleaning insectivore | Velvet-fronted Nuthatch | VlfNut | 0.27 | 0.09 |
| Small mid-canopy gleaning insectivore | White-bellied Erpornis | WtbErp | 0.07 | 0.01 |
| Small mid-canopy gleaning insectivore | Whistler's Warbler | WstWar | 0.07 | 0.01 |
| Small mid-canopy gleaning insectivore | Oriental White-eye | OrtWte | 0.07 | 0.49 |
| Small mid-canopy gleaning insectivore | Greenish Warbler | GrnWar | 0.03 | 0.01 |
| Small mid-canopy gleaning insectivore | Common Iora | ComIor | 0.03 | 0.47 |
| Small low-canopy gleaning insectivore | Puff-throated Babbler | PftBab | 0.27 | 0.21 |
| Small low-canopy gleaning insectivore | Nepal Fulvetta | NepFul | 0.23 | 0.07 |
| Small low-canopy gleaning insectivore | Rufous-capped Babbler | RfcBab | 0.10 | 0.39 |
| Small low-canopy gleaning insectivore | Common Tailorbird | ComTlr | 0.07 | 0.87 |
| Small low-canopy gleaning insectivore | Siberian Rubythroat | SibRub | 0.00 | 0.09 |
| Small low-canopy gleaning insectivore | Rufescent Prinia | RufPri | 0.00 | 0.09 |
| Small low-canopy gleaning insectivore | Grey-breasted Prinia | GrbPri | 0.00 | 0.01 |
| Small mid-canopy sallying insectivore | Grey-headed Canary Flycatcher | GhcFly | 0.77 | 0.51 |
| Small mid-canopy sallying insectivore | Pale-chinned Flycatcher | PlcFly | 0.43 | 0.04 |
| Small mid-canopy sallying insectivore | White-throated Fantail | WttFan | 0.17 | 0.03 |
| Small mid-canopy sallying insectivore | Black-naped Monarch | BlnMon | 0.07 | 0.07 |
| Small mid-canopy sallying insectivore | Rufous-bellied Niltava | RfbNil | 0.10 | 0.00 |
| Small mid-canopy sallying insectivore | Taiga Flycatcher | TaiFly | 0.03 | 0.10 |
| Small mid-canopy sallying insectivore | Bar-winged Flycatcher-Shrike | BwgFls | 0.03 | 0.06 |
| Small mid-canopy sallying insectivore | Pale-blue Flycatcher | PlbFly | 0.03 | 0.00 |
| Small mid-canopy sallying insectivore | Little Pied Flycatcher | LtpFly | 0.00 | 0.01 |
| Small mid-canopy sallying insectivore | Blue-throated Blue Flycatcher | BltBfl | 0.00 | 0.07 |
| Large woodpecker | Rufous Woodpecker | RfsWdp | 0.67 | 0.29 |
| Large woodpecker | Bay Woodpecker | BayWdp | 0.47 | 0.06 |
| Large woodpecker | Lesser Yellownape | LesYln | 0.10 | 0.03 |
| Large woodpecker | Grey-headed Woodpecker | GrhWdp | 0.07 | 0.03 |
| Large woodpecker | Greater Goldenback | GrtGob | 0.03 | 0.03 |
| Large woodpecker | Greater Yellownape | GrtYln | 0.03 | 0.00 |
| Large woodpecker | Great Slaty Woodpecker | GrsWdp | 0.03 | 0.00 |
| Other | Grey-capped Pygmy Woodpecker | GcpWdp | 0.17 | 0.11 |
| Other | Oriental Pied Hornbill | OrpHrb | 0.13 | 0.01 |
| Other | Grey-backed Shrike | GrbShr | 0.03 | 0.21 |
| Other | White-browed Piculet | WtbPic | 0.03 | 0.00 |
| Other | Speckled Piculet | SpcPic | 0.03 | 0.00 |
| Other | Great Hornbill | GrtHrb | 0.03 | 0.00 |

**Table G.** Bird Species found inside the PA and in the agricultural matrix outside the PA

| **PA** | **Agricultural matrix** |
| --- | --- |
| Ashy bulbul | Ashy bulbul |
| Asian fairy bluebird | Ashy drongo |
| Bar-winged flycatcher-shrike | Asian fairy bluebird |
| Bay woodpecker | Bar-winged flycatcher-shrike |
| Black-crested bulbul | Bay woodpecker |
| Black-hooded oriole | Black-crested bulbul |
| Black-naped monarch | Black-hooded oriole |
| Black bulbul | Black-naped monarch |
| Black drongo | Black bulbul |
| Blue-bearded bee-eater | Black drongo |
| Blue-eared barbet | Blue-bearded bee-eater |
| Blue-throated barbet | Blue-throated barbet |
| Blue whistling thrush | Blue-throated blue flycatcher |
| Common hill myna | Blue whistling thrush |
| Common iora | Bronzed drongo |
| Common tailorbird | Chestnut-tailed starling |
| Crimson sunbird | Common hill myna |
| Dollarbird | Common iora |
| Emerald dove | Common tailorbird |
| Golden-fronted leafbird | Crimson sunbird |
| Great barbet | Dollarbird |
| Great hornbill | Eurasian collared dove |
| Great slaty woodpecker | Fire-breasted flowerpecker |
| Greater goldenback | Fire-tailed sunbird |
| Greater racket-tailed drongo | Golden-fronted leafbird |
| Greater yellownape | Great barbet |
| Green-billed malkoha | Greater coucal |
| Greenish warbler | Greater goldenback |
| Grey-backed shrike | Green-billed malkoha |
| Grey-capped pygmy woodpecker | Greenish warbler |
| Grey-headed canary flycatcher | Grey-backed shrike |
| Grey-headed woodpecker | Grey-breasted prinia |
| Grey treepie | Grey-capped pygmy woodpecker |
| Indian cuckoo | Grey-headed canary flycatcher |
| Jungle crow | Grey-headed woodpecker |
| Large cuckooshrike | Grey treepie |
| Large woodshrike | Hawk cuckoo |
| Lesser necklaced laughingthrush | Indian cuckoo |
| Lesser racket-tailed drongo | Jungle babbler |
| Lesser yellownape | Jungle crow |
| Little spiderhunter | Large cuckooshrike |
| Long-tailed broadbill | Large woodshrike |
| Maroon oriole | Lesser necklaced laughingthrush |
| Nepal fulvetta | Lesser racket-tailed drongo |
| Oriental pied hornbill | Lesser yellownape |
| Oriental turtle dove | Lineated barbet |
| Oriental white eye | Little pied flycatcher |
| Pale-blue flycatcher | Little spiderhunter |
| Pale-chinned flycatcher | Long-tailed broadbill |
| Plain flowerpecker | Maroon oriole |
| Puff-throated babbler | Nepal fulvetta |
| Ruby-cheeked sunbird | Oriental magpie robin |
| Rufous-bellied niltava | Oriental pied hornbill |
| Rufous-capped babbler | Oriental turtle dove |
| Rufous woodpecker | Oriental white eye |
| Scarlet minivet | Pale-chinned flycatcher |
| Spangled drongo | Plain flowerpecker |
| Speckled piculet | Puff-throated babbler |
| Streaked spiderhunter | Red-vented bulbul |
| Sultan tit | Red-whiskered bulbul |
| Taiga flycatcher | Ruby-cheeked sunbird |
| Velvet-fronted nuthatch | Rufescent prinia |
| Whistler's warbler | Rufous-capped babbler |
| White-bellied erpornis | Rufous-necked laughingthrush |
| White-browed piculet | Rufous woodpecker |
| White-browed scimitar babbler | Scarlet-backed flowerpecker |
| White-crested laughingthrush | Scarlet minivet |
| White-hooded babbler | Siberian rubythroat |
| White-rumped munia | Spangled drongo |
| White-rumped shama | Streaked spiderhunter |
| White-throated bulbul | Sultan tit |
| White-throated fantail flycatcher | Taiga flycatcher |
| Yellow-bellied warbler | Velvet-fronted nuthatch |
| Yellow-browed warbler | Whistler's warbler |
|  | White-bellied erpornis |
|  | White-browed scimitar babbler |
|  | White-hooded babbler |
|  | White-rumped munia |
|  | White-rumped shama |
|  | White-throated bulbul |
|  | White-throated fantail flycatcher |
|  | Yellow-bellied warbler |
|  | Yellow-browed warbler |
|  | Yellow-eyed babbler |


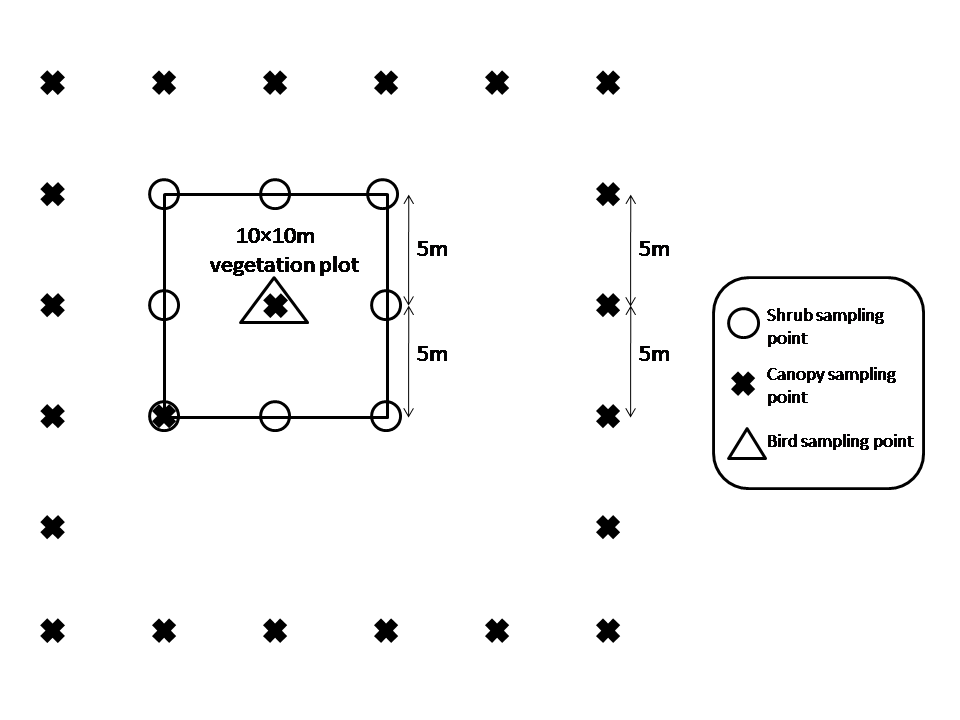


**Fig A.** Schematic representation showing sampling of vegetation structure at each sampling point (shown as a triangle). Basal area was calculated within the 10m × 10m plot marked with a solid black line. Eight measurements of shrub height were taken (depicted as open circles) at the perimeter of this plot. 22 measurements of canopy and bamboo cover were taken at the points marked as a cross within and surrounding this plot.

**
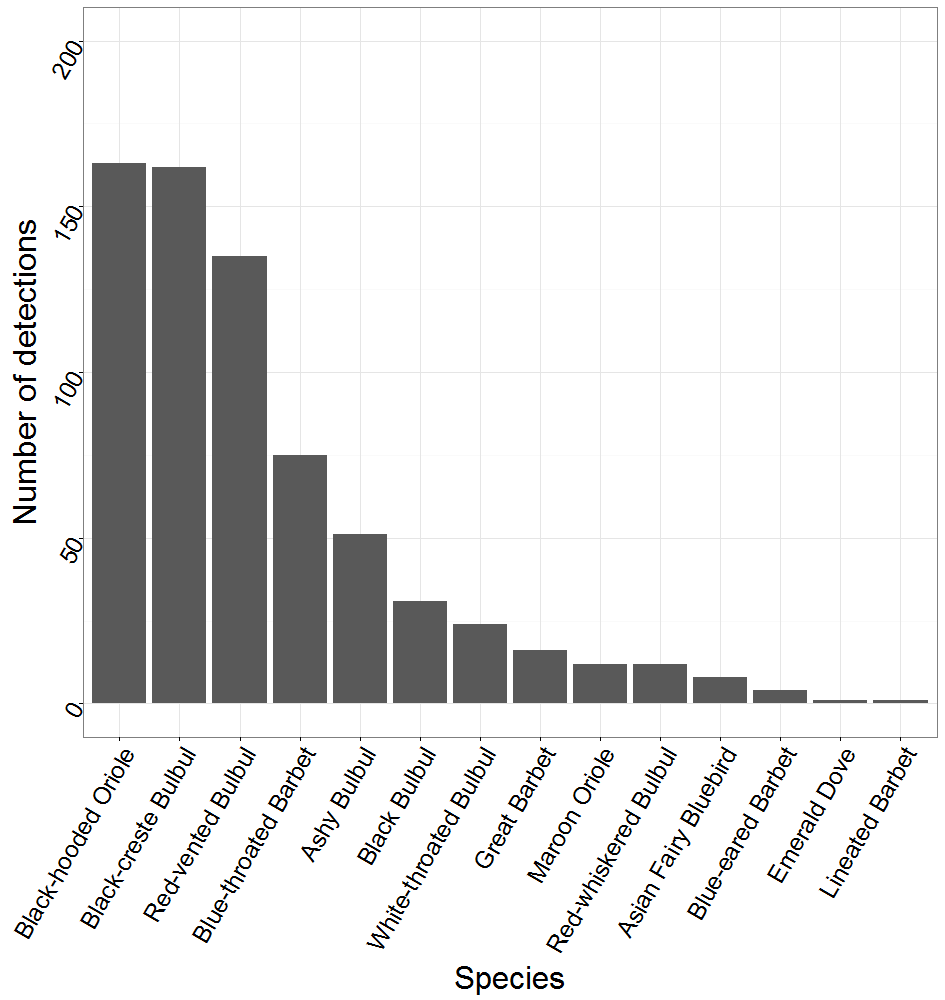
**

**Fig B.** Number of detections for each species of the frugivorous guild during the study. The high naïve occupancy (0.99) of the guild was driven by three abundant species – Black-hooded oriole, Black-crested bulbul and Red-vented bulbul.
