# Supplementary material for: “In a tree by the brook, there’s a songbird who sings”: Woodlands in an agricultural matrix maintain functionality of a wintering bird community
Source: PLoS One. 2018 Aug 2;13(8):e0201657. doi: 10.1371/journal.pone.0201657 (PMC6072076; doi:10.1371/journal.pone.0201657)
Supplement: S2 File — Model selection was conducted using AIC, while removing all uninformative models. Chosen models for inference are highlighted in bold. (DOCX) [file pone.0201657.s002.docx]

**S2 File. Model selection of site-use and detection probability for different guilds using an occupancy-based approach.** Model selection was conducted using AIC, while removing all uninformative models. Chosen models for inference are highlighted in bold.

Nectarivores

| **Model** | **nPar** | **AIC** | **ΔAIC** | **AIC Weight** | **2negLL** |
| --- | --- | --- | --- | --- | --- |
| **psi(shrub cover), p(.)** | **3** | **680.56** | **0.00** | **0.10** | **674.56** |
| psi(canopy cover + shrub cover), p(.) | 4 | 681.03 | 0.47 | 0.08 | 673.03 |
| psi(stand basal area + shrub cover), p(.) | 4 | 681.44 | 0.88 | 0.07 | 673.44 |
| psi(shrub cover), p(canopy cover) | 4 | 682.34 | 1.78 | 0.04 | 674.34 |
| psi(bamboo cover + shrub cover), p(.) | 4 | 682.41 | 1.85 | 0.04 | 674.41 |
| psi(shrub cover), p(shrub cover) | 4 | 682.42 | 1.86 | 0.04 | 674.42 |
| psi(shrub cover), p(time from sunrise) | 4 | 682.52 | 1.96 | 0.04 | 674.52 |
| psi(canopy cover), p(.) | 3 | 682.66 | 2.11 | 0.04 | 676.66 |
| psi(canopy cover + shrub cover),p (canopy cover) | 5 | 682.71 | 2.15 | 0.04 | 672.71 |
| psi(canopy cover + shrub cover), p(shrub cover) | 5 | 682.85 | 2.29 | 0.03 | 672.85 |
| psi(canopy cover + bamboo cover + shrub cover), p(.) | 5 | 682.92 | 2.37 | 0.03 | 672.92 |
| psi(canopy cover + shrub cover), p(time from sunrise) | 5 | 683.02 | 2.46 | 0.03 | 673.02 |
| psi(stand basal area + shrub cover), p(canopy cover) | 5 | 683.21 | 2.65 | 0.03 | 673.21 |
| psi(stand basal area + shrub cover), p(shrub cover) | 5 | 683.31 | 2.75 | 0.03 | 673.31 |
| psi(.), p(.) | 2 | 683.34 | 2.78 | 0.03 | 679.34 |
| psi(stand basal area + shrub cover), p(time from sunrise) | 5 | 683.41 | 2.85 | 0.02 | 673.41 |
| psi(distance to PA), p(.) | 3 | 683.96 | 3.40 | 0.02 | 677.96 |
| psi(bamboo cover + shrub cover), p(canopy cover) | 5 | 684.17 | 3.61 | 0.02 | 674.17 |
| psi(bamboo cover), p(.) | 3 | 684.18 | 3.62 | 0.02 | 678.18 |
| psi(bamboo cover + shrub cover), p(shrub cover) | 5 | 684.27 | 3.71 | 0.02 | 674.27 |
| psi(bamboo cover + shrub cover), p(time from sunrise) | 5 | 684.38 | 3.82 | 0.02 | 674.38 |
| psi(canopy cover), p(canopy cover) | 4 | 684.48 | 3.92 | 0.01 | 676.48 |
| psi(stand basal area), p(.) | 3 | 684.53 | 3.97 | 0.01 | 678.53 |
| psi(canopy cover), p(shrub cover) | 4 | 684.59 | 4.04 | 0.01 | 676.59 |
| psi(canopy cover + bamboo cover), p(.) | 4 | 684.61 | 4.05 | 0.01 | 676.61 |
| psi(canopy cover + bamboo cover + shrub cover), p(canopy cover) | 6 | 684.61 | 4.05 | 0.01 | 672.61 |
| psi(canopy cover), p(time from sunrise) | 4 | 684.64 | 4.08 | 0.01 | 676.64 |
| psi(canopy cover + bamboo cover + shrub cover + distance to PA), p(.) | 6 | 684.73 | 4.18 | 0.01 | 672.73 |
| psi(canopy cover + bamboo cover + shrub cover), p(shrub cover) | 6 | 684.74 | 4.18 | 0.01 | 672.74 |
| psi(canopy cover + bamboo cover + shrub cover), p(time from sunrise) | 6 | 684.91 | 4.35 | 0.01 | 672.91 |
| psi(.), p(time from sunrise) | 3 | 685.15 | 4.59 | 0.01 | 679.15 |
| psi(.), p(canopy cover) | 3 | 685.34 | 4.78 | 0.01 | 679.34 |
| psi(.), p(shrub cover) | 3 | 685.34 | 4.78 | 0.01 | 679.34 |
| psi(distance to PA), p(time from sunrise) | 4 | 685.81 | 5.25 | 0.01 | 677.81 |
| psi(distance to PA), p(canopy cover) | 4 | 685.94 | 5.38 | 0.01 | 677.94 |
| psi(distance to PA), p(shrub cover) | 4 | 685.95 | 5.39 | 0.01 | 677.95 |
| psi(bamboo cover), p(time from sunrise) | 4 | 686.02 | 5.46 | 0.01 | 678.02 |
| psi(bamboo cover), p(canopy cover) | 4 | 686.16 | 5.61 | 0.01 | 678.16 |
| psi(bamboo cover), p(shrub cover) | 4 | 686.18 | 5.62 | 0.01 | 678.18 |
| psi(canopy cover + bamboo cover + shrub cover + distance to PA), p(canopy cover) | 7 | 686.43 | 5.87 | 0.01 | 672.43 |
| psi(canopy cover + bamboo cover), p(canopy cover) | 5 | 686.43 | 5.87 | 0.01 | 676.43 |
| psi(stand basal area), p(time from sunrise) | 4 | 686.44 | 5.88 | 0.01 | 678.44 |
| psi(stand basal area), p(canopy cover) | 4 | 686.51 | 5.95 | 0.01 | 678.51 |
| psi(stand basal area), p(shrub cover) | 4 | 686.53 | 5.97 | 0.01 | 678.53 |
| psi(canopy cover + bamboo cover), p(shrub cover) | 5 | 686.54 | 5.98 | 0.01 | 676.54 |
| psi(canopy cover + bamboo cover + shrub cover + distance to PA), p(shrub cover) | 7 | 686.55 | 6.00 | 0.01 | 672.55 |
| psi(canopy cover + bamboo cover), p(time from sunrise) | 5 | 686.59 | 6.03 | 0.01 | 676.59 |
| psi(canopy cover + bamboo cover + shrub cover + distance to PA), p(time from sunrise) | 7 | 686.72 | 6.16 | 0.00 | 672.72 |

Granivores

| Model | **nPar** | **AIC** | **ΔAIC** | **AIC Weight** | **2negLL** |
| --- | --- | --- | --- | --- | --- |
| **psi(canopy cover + bamboo cover), p(time from sunrise)** | **5** | **388.07** | **0.00** | **0.12** | **378.07** |
| **psi(bamboo cover + shrub cover), p(time from sunrise + canopy cover)** | **6** | **388.22** | **0.14** | **0.12** | **376.22** |
| **psi(bamboo cover + shrub cover), p(time from sunrise)** | **5** | **388.48** | **0.40** | **0.10** | **378.48** |
| **psi(canopy cover + bamboo cover), p(time from sunrise + canopy cover)** | **6** | **388.71** | **0.64** | **0.09** | **376.71** |
| **psi(canopy cover + bamboo cover + shrub cover), p(time from sunrise)** | **6** | **388.82** | **0.75** | **0.09** | **376.82** |
| **psi(canopy cover + bamboo cover + shrub cover), p(time from sunrise + canopy cover)** | **7** | **389.37** | **1.30** | **0.07** | **375.37** |
| **psi(canopy cover + bamboo cover), p(time from sunrise + shrub cover)** | **6** | **389.65** | **1.58** | **0.06** | **377.65** |
| **psi(bamboo cover), p(time from sunrise + canopy cover)** | **5** | **390.01** | **1.93** | **0.05** | **380.01** |
| psi(bamboo cover + shrub cover), p(time from sunrise + shrub cover) | 6 | 390.31 | 2.24 | 0.04 | 378.31 |
| psi(canopy cover + bamboo cover + shrub cover), p(time from sunrise + shrub cover) | 7 | 390.64 | 2.57 | 0.03 | 376.64 |
| psi(canopy cover + bamboo cover + shrub cover + distance to PA), p(time from sunrise) | 7 | 390.7 | 2.63 | 0.03 | 376.7 |
| psi(canopy cover), p(time from sunrise) | 4 | 391.17 | 3.09 | 0.03 | 383.17 |
| psi(canopy cover + bamboo cover + shrub cover + distance to PA), p(time from sunrise + canopy cover) | 8 | 391.21 | 3.13 | 0.03 | 375.21 |
| psi(bamboo cover), p(time from sunrise) | 4 | 391.43 | 3.35 | 0.02 | 383.43 |
| psi(canopy cover), p(time from sunrise + canopy cover) | 5 | 391.53 | 3.45 | 0.02 | 381.53 |
| psi(canopy cover + shrub cover), p(time from sunrise) | 5 | 391.98 | 3.91 | 0.02 | 381.98 |
| psi(canopy cover + shrub cover), p(time from sunrise + canopy cover) | 6 | 392.24 | 4.16 | 0.02 | 380.24 |
| psi(bamboo cover), p(time from sunrise + shrub cover) | 5 | 392.47 | 4.40 | 0.01 | 382.47 |
| psi(canopy cover + bamboo cover + shrub cover + distance to PA), p(time from sunrise + shrub cover) | 8 | 392.49 | 4.42 | 0.01 | 376.49 |
| psi(canopy cover), p(time from sunrise + shrub cover) | 5 | 392.71 | 4.64 | 0.01 | 382.71 |
| psi(shrub cover), p(time from sunrise + canopy cover) | 5 | 393.28 | 5.21 | 0.01 | 383.28 |
| psi(canopy cover + shrub cover), p(time from sunrise + shrub cover) | 6 | 393.76 | 5.69 | 0.01 | 381.76 |
| psi(stand basal area + shrub cover), p(time from sunrise + canopy cover) | 6 | 393.91 | 5.84 | 0.01 | 381.91 |
| psi(shrub cover), p(time from sunrise) | 4 | 395.77 | 7.70 | 0.00 | 387.77 |
| psi(.), p(time from sunrise + canopy cover) | 4 | 397.25 | 9.18 | 0.00 | 389.25 |
| psi(shrub cover), p(time from sunrise + shrub cover) | 5 | 397.48 | 9.41 | 0.00 | 387.48 |
| psi(stand basal area + shrub cover), p(time from sunrise) | 5 | 397.52 | 9.44 | 0.00 | 387.52 |
| psi(distance to PA), p(time from sunrise + canopy cover) | 5 | 399.14 | 11.07 | 0.00 | 389.14 |
| psi(stand basal area + shrub cover), p(time from sunrise + shrub cover) | 6 | 399.23 | 11.16 | 0.00 | 387.23 |
| psi(stand basal area), p(time from sunrise + canopy cover) | 5 | 399.24 | 11.17 | 0.00 | 389.24 |
| psi(.), p(time from sunrise) | 3 | 403.44 | 15.37 | 0.00 | 397.44 |
| psi(.), p(time from sunrise + shrub cover) | 4 | 403.66 | 15.59 | 0.00 | 395.66 |
| psi(distance to PA), p(time from sunrise) | 4 | 404.51 | 16.44 | 0.00 | 396.51 |
| psi(stand basal area), p(time from sunrise) | 4 | 404.58 | 16.50 | 0.00 | 396.58 |
| psi(stand basal area), p(time from sunrise + shrub cover) | 5 | 405 | 16.93 | 0.00 | 395 |
| psi(distance to PA), p(time from sunrise + shrub cover) | 5 | 405.09 | 17.02 | 0.00 | 395.09 |

Omnivores

| **Model** | **nPar** | **AIC** | **ΔAIC** | **AIC Weight** | **2negLL** |
| --- | --- | --- | --- | --- | --- |
| **psi(bamboo cover), p(shrub cover)** | **4** | **436.52** | **0.00** | **0.11** | **428.52** |
| **psi(bamboo cover), p(canopy cover + shrub cover)** | **5** | **437.23** | **0.71** | **0.08** | **427.23** |
| **psi(bamboo cover), p(time from sunrise + shrub cover)** | **5** | **437.38** | **0.86** | **0.07** | **427.38** |
| **psi(canopy cover), p(shrub cover)** | **4** | **437.4** | **0.87** | **0.07** | **429.4** |
| **psi(canopy cover), p(time from sunrise + shrub cover)** | **5** | **437.78** | **1.25** | **0.06** | **427.78** |
| **psi(canopy cover + bamboo cover), p(shrub cover)** | **5** | **438.02** | **1.49** | **0.05** | **428.02** |
| psi(bamboo cover + shrub cover), p(shrub cover) | 5 | 438.52 | 1.99 | 0.04 | 428.52 |
| psi(canopy cover + bamboo cover), p(canopy cover + shrub cover) | 6 | 438.74 | 2.21 | 0.04 | 426.74 |
| psi(canopy cover + bamboo cover), p(time from sunrise + shrub cover) | 6 | 438.89 | 2.37 | 0.03 | 426.89 |
| psi(canopy cover + shrub cover), p(shrub cover) | 5 | 439.06 | 2.54 | 0.03 | 429.06 |
| psi(canopy cover), p(canopy cover + shrub cover) | 5 | 439.18 | 2.65 | 0.03 | 429.18 |
| psi(bamboo cover + shrub cover), p(canopy cover + shrub cover) | 6 | 439.23 | 2.71 | 0.03 | 427.23 |
| psi(.), p(canopy cover + shrub cover) | 4 | 439.34 | 2.82 | 0.03 | 431.34 |
| psi(canopy cover + shrub cover), p(time from sunrise + shrub cover) | 6 | 439.35 | 2.82 | 0.03 | 427.35 |
| psi(bamboo cover + shrub cover), p(time from sunrise + shrub cover) | 6 | 439.35 | 2.83 | 0.03 | 427.35 |
| psi(bamboo cover + shrub cover), p(.) | 4 | 439.75 | 3.23 | 0.02 | 431.75 |
| psi(canopy cover + bamboo cover + shrub cover), p(shrub cover) | 6 | 440 | 3.48 | 0.02 | 428 |
| psi(.), p(shrub cover) | 3 | 440.51 | 3.99 | 0.02 | 434.51 |
| psi(.), p(time from sunrise + shrub cover) | 4 | 440.53 | 4.01 | 0.02 | 432.53 |
| psi(canopy cover + bamboo cover + shrub cover), p(canopy cover + shrub cover) | 7 | 440.65 | 4.13 | 0.01 | 426.65 |
| psi(bamboo cover + shrub cover), p(time from sunrise) | 5 | 440.69 | 4.17 | 0.01 | 430.69 |
| psi(canopy cover + bamboo cover + shrub cover), p(time from sunrise + shrub cover) | 7 | 440.85 | 4.32 | 0.01 | 426.85 |
| psi(stand basal area), p(canopy cover + shrub cover) | 5 | 440.93 | 4.41 | 0.01 | 430.93 |
| psi(canopy cover + bamboo cover + shrub cover + distance to PA), p(shrub cover) | 7 | 440.95 | 4.43 | 0.01 | 426.95 |
| psi(shrub cover), p(canopy cover + shrub cover) | 5 | 441.26 | 4.74 | 0.01 | 431.26 |
| psi(canopy cover + bamboo cover + shrub cover + distance to PA), p(.) | 6 | 441.58 | 5.05 | 0.01 | 429.58 |
| psi(canopy cover + bamboo cover + shrub cover), p(.) | 5 | 441.72 | 5.20 | 0.01 | 431.72 |
| psi(canopy cover + bamboo cover + shrub cover + distance to PA), p(time from sunrise + shrub cover) | 8 | 441.81 | 5.29 | 0.01 | 425.81 |
| psi(canopy cover + bamboo cover + shrub cover + distance to PA), p(canopy cover + shrub cover) | 8 | 441.94 | 5.42 | 0.01 | 425.94 |
| psi(shrub cover), p(shrub cover) | 4 | 442.13 | 5.61 | 0.01 | 434.13 |
| psi(shrub cover), p(time from sunrise + shrub cover) | 5 | 442.15 | 5.62 | 0.01 | 432.15 |
| psi(shrub cover), p(.) | 3 | 442.39 | 5.86 | 0.01 | 436.39 |
| psi(stand basal area), p(shrub cover) | 4 | 442.43 | 5.90 | 0.01 | 434.43 |
| psi(stand basal area), p(time from sunrise + shrub cover) | 5 | 442.45 | 5.93 | 0.01 | 432.45 |
| psi(canopy cover + bamboo cover + shrub cover + distance to PA), p(time from sunrise) | 7 | 442.5 | 5.98 | 0.01 | 428.5 |
| psi(stand basal area + shrub cover), p(canopy cover + shrub cover) | 6 | 442.63 | 6.10 | 0.01 | 430.63 |
| psi(shrub cover), p(time from sunrise) | 4 | 442.63 | 6.11 | 0.01 | 434.63 |
| psi(canopy cover + bamboo cover + shrub cover), p(time from sunrise) | 6 | 442.65 | 6.13 | 0.01 | 430.65 |
| psi(canopy cover + shrub cover), p(.) | 4 | 443.53 | 7.00 | 0.00 | 435.53 |
| psi(stand basal area + shrub cover), p(.) | 4 | 444.01 | 7.49 | 0.00 | 436.01 |
| psi(canopy cover + shrub cover), p(time from sunrise) | 5 | 444.03 | 7.50 | 0.00 | 434.03 |
| psi(stand basal area + shrub cover), p(shrub cover) | 5 | 444.12 | 7.59 | 0.00 | 434.12 |
| psi(stand basal area + shrub cover), p(time from sunrise + shrub cover) | 6 | 444.13 | 7.60 | 0.00 | 432.13 |
| psi(stand basala area + shrub cover), p(time from sunrise) | 5 | 444.32 | 7.80 | 0.00 | 434.32 |
| psi(bamboo cover), p(.) | 3 | 444.58 | 8.06 | 0.00 | 438.58 |
| psi(bamboo cover), p(time from sunrise) | 4 | 444.92 | 8.40 | 0.00 | 436.92 |
| psi(.), p(time from sunrise) | 3 | 445.15 | 8.63 | 0.00 | 439.15 |
| psi(.), p(.) | 2 | 445.36 | 8.84 | 0.00 | 441.36 |
| psi(canopy cover + bamboo cover), p(.) | 4 | 445.45 | 8.92 | 0.00 | 437.45 |
| psi(canopy cover + bamboo cover), p(time from sunrise) | 5 | 445.8 | 9.27 | 0.00 | 435.8 |
| psi(stand basal area), p(time from sunrise) | 4 | 446.96 | 10.43 | 0.00 | 438.96 |
| psi(distance to PA), p(time from sunrise) | 4 | 447 | 10.48 | 0.00 | 439 |
| psi(canopy cover), p(time from sunrise) | 4 | 447.1 | 10.57 | 0.00 | 439.1 |
| psi(stand basal area), p(.) | 3 | 447.14 | 10.62 | 0.00 | 441.14 |
| psi(distance to PA), p(.) | 3 | 447.23 | 10.71 | 0.00 | 441.23 |
| psi(canopy cover), p(.) | 3 | 447.35 | 10.82 | 0.00 | 441.35 |

Frugivores

| **Model** | **nPar** | **AIC** | **ΔAIC** | **AIC Weight** | **2negLL** |
| --- | --- | --- | --- | --- | --- |
| **psi(.), p(shrub cover)** | **3** | **568.76** | **0.00** | **0.06** | **562.76** |
| **psi(.), p(.)** | **2** | **568.76** | **0.00** | **0.06** | **564.76** |
| psi(.), p(time from sunrise + shrub cover) | 4 | 569.43 | 0.67 | 0.05 | 561.43 |
| psi(.), p(time from sunrise) | 3 | 569.74 | 0.97 | 0.04 | 563.74 |
| psi(shrub cover), p(.) | 3 | 569.93 | 1.17 | 0.04 | 563.93 |
| psi(bamboo cover), p(.) | 3 | 570.05 | 1.29 | 0.03 | 564.05 |
| psi(shrub cover), p(shrub cover) | 4 | 570.1 | 1.34 | 0.03 | 562.1 |
| psi(bamboo cover), p(shrub cover) | 4 | 570.15 | 1.38 | 0.03 | 562.15 |
| psi(distance to PA), p(shrub cover) | 4 | 570.37 | 1.61 | 0.03 | 562.37 |
| psi(.), p(canopy cover) | 3 | 570.47 | 1.71 | 0.03 | 564.47 |
| psi(distance to PA), p(.) | 3 | 570.72 | 1.96 | 0.02 | 564.72 |
| psi(canopy cover), p(.) | 3 | 570.75 | 1.98 | 0.02 | 564.75 |
| psi(stand basal area), p(.) | 3 | 570.76 | 1.99 | 0.02 | 564.76 |
| psi(canopy cover), p(shrub cover) | 4 | 570.76 | 2.00 | 0.02 | 562.76 |
| psi(stand basal area), p(shrub cover) | 4 | 570.76 | 2.00 | 0.02 | 562.76 |
| psi(shrub cover), p(time from sunrise + shrub cover) | 5 | 570.8 | 2.04 | 0.02 | 560.8 |
| psi(bamboo cover), p(time from sunrise + shrub cover) | 5 | 570.85 | 2.09 | 0.02 | 560.85 |
| psi(shrub cover), p(time from sunrise) | 4 | 570.85 | 2.09 | 0.02 | 562.85 |
| psi(bamboo cover), p(time from sunrise) | 4 | 571.03 | 2.26 | 0.02 | 563.03 |
| psi(distance to PA), p(time from sunrise + shrub cover) | 5 | 571.06 | 2.30 | 0.02 | 561.06 |
| psi(distance to PA), p(time from sunrise) | 4 | 571.12 | 2.36 | 0.02 | 563.12 |
| psi(canopy cover), p(time from sunrise + shrub cover) | 5 | 571.42 | 2.66 | 0.02 | 561.42 |
| psi(stand basal area), p(time from sunrise + shrub cover) | 5 | 571.43 | 2.66 | 0.02 | 561.43 |
| psi(bamboo cover + shrub cover), p(.) | 4 | 571.49 | 2.72 | 0.02 | 563.49 |
| psi(bamboo cover + shrub cover), p(shrub cover) | 5 | 571.68 | 2.91 | 0.01 | 561.68 |
| psi(shrub cover), p(canopy cover) | 4 | 571.68 | 2.92 | 0.01 | 563.68 |
| psi(stand basal area), p(time from sunrise) | 4 | 571.73 | 2.96 | 0.01 | 563.73 |
| psi(canopy cover + shrub cover), p(.) | 4 | 571.77 | 3.01 | 0.01 | 563.77 |
| psi(bamboo cover), p(canopy cover) | 4 | 571.78 | 3.01 | 0.01 | 563.78 |
| psi(stand basal area + shrub cover), p(.) | 4 | 571.87 | 3.11 | 0.01 | 563.87 |
| psi(canopy cover + shrub cover), p(shrub cover) | 5 | 571.89 | 3.13 | 0.01 | 561.89 |
| psi(distance to PA), p(canopy cover) | 4 | 571.96 | 3.20 | 0.01 | 563.96 |
| psi(canopy cover + bamboo cover), p(.) | 4 | 571.98 | 3.22 | 0.01 | 563.98 |
| psi(canopy cover + bamboo cover), p(shrub cover) | 5 | 572 | 3.24 | 0.01 | 562 |
| psi(bamboo cover + shrub cover), p(time from sunrise + shrub cover) | 6 | 572.38 | 3.62 | 0.01 | 560.38 |
| psi(bamboo cover + shrub cover), p(time from sunrise) | 5 | 572.4 | 3.64 | 0.01 | 562.4 |
| psi(canopy cover), p(canopy cover) | 4 | 572.46 | 3.70 | 0.01 | 564.46 |
| psi(stand basal area), p(canopy cover) | 4 | 572.47 | 3.70 | 0.01 | 564.47 |
| psi(canopy cover + shrub cover), p(time from sunrise + shrub cover) | 6 | 572.61 | 3.85 | 0.01 | 560.61 |
| psi(canopy cover + bamboo cover), p(time from sunrise + shrub cover) | 6 | 572.69 | 3.92 | 0.01 | 560.69 |
| psi(stand basal area + shrub cover), p(time from sunrise + shrub cover) | 6 | 572.73 | 3.97 | 0.01 | 560.73 |
| psi(canopy cover), p(time from sunrise) | 4 | 572.74 | 3.98 | 0.01 | 564.74 |
| psi(stand basal area + shrub cover), p(time from sunrise) | 5 | 572.81 | 4.04 | 0.01 | 562.81 |
| psi(canopy cover + bamboo cover + shrub cover), p(.) | 5 | 572.92 | 4.15 | 0.01 | 562.92 |
| psi(canopy cover + bamboo cover), p(time from sunrise) | 5 | 572.97 | 4.21 | 0.01 | 562.97 |
| psi(canopy cover + bamboo cover + shrub cover), p(shrub cover) | 6 | 573.03 | 4.27 | 0.01 | 561.03 |
| psi(bamboo cover + shrub cover), p(canopy cover) | 5 | 573.24 | 4.48 | 0.01 | 563.24 |
| psi(canopy cover + shrub cover), p(canopy cover) | 5 | 573.5 | 4.74 | 0.01 | 563.5 |
| psi(stand basal area + shrub cover), p(canopy cover) | 5 | 573.61 | 4.85 | 0.01 | 563.61 |
| psi(canopy cover + shrub cover), p(time from sunrise) | 5 | 573.64 | 4.87 | 0.01 | 563.64 |
| psi(canopy cover + bamboo cover), p(canopy cover) | 5 | 573.69 | 4.93 | 0.01 | 563.69 |
| psi(canopy cover + bamboo cover + shrub cover), p(time from sunrise + shrub cover) | 7 | 573.78 | 5.01 | 0.01 | 559.78 |
| psi(canopy cover + bamboo cover + shrub cover), p(time from sunrise) | 6 | 573.91 | 5.15 | 0.00 | 561.91 |
| psi(stand basal area + shrub cover), p(shrub cover) | 5 | 574.04 | 5.27 | 0.00 | 564.04 |
| psi(canopy cover + bamboo cover + shrub cover + distance to PA), p(.) | 6 | 574.57 | 5.81 | 0.00 | 562.57 |
| psi(canopy cover + bamboo cover + shrub cover), p(canopy cover) | 6 | 574.64 | 5.88 | 0.00 | 562.64 |
| psi(canopy cover + bamboo cover + shrub cover + distance to PA), p(shrub cover) | 7 | 574.74 | 5.97 | 0.00 | 560.74 |
| psi(canopy cover + bamboo cover + shrub cover + distance to PA), p(time from sunrise + shrub cover) | 8 | 575.48 | 6.72 | 0.00 | 559.48 |
| psi(canopy cover + bamboo cover + shrub cover + distance to PA), p(time from sunrise) | 7 | 575.54 | 6.77 | 0.00 | 561.54 |
| psi(canopy cover + bamboo cover + shrub cover + distance to PA), p(canopy cover) | 7 | 576.31 | 7.54 | 0.00 | 562.31 |

Large high-canopy gleaning insectivores

| **Model** | **nPar** | **AIC** | **ΔAIC** | **AIC Weight** | **2negLL** |
| --- | --- | --- | --- | --- | --- |
| **psi(canopy cover), p(time from sunrise)** | **4** | **655.82** | **0.00** | **0.09** | **647.82** |
| psi(canopy cover + bamboo cover), p(time from sunrise) | 5 | 655.89 | 0.07 | 0.09 | 645.89 |
| **psi(distance to PA), p(time from sunrise)** | **4** | **656.4** | **0.59** | **0.07** | **648.4** |
| **psi(.), p(time from sunrise)** | **3** | **656.61** | **0.79** | **0.06** | **650.61** |
| psi(canopy cover), p(time from sunrise + shrub cover) | 5 | 657.13 | 1.32 | 0.05 | 647.13 |
| psi(canopy cover + bamboo cover), p(time from sunrise + shrub cover) | 6 | 657.16 | 1.34 | 0.05 | 645.16 |
| psi(canopy cover + bamboo cover + shrub cover + distance to PA), p(time from sunrise) | 7 | 657.46 | 1.64 | 0.04 | 643.46 |
| psi(canopy cover), p(time from sunrise + canopy cover) | 5 | 657.74 | 1.92 | 0.03 | 647.74 |
| psi(canopy cover + shrub cover), p(time from sunrise) | 5 | 657.79 | 1.98 | 0.03 | 647.79 |
| psi(canopy cover + bamboo cover), p(time from sunrise + canopy cover) | 6 | 657.85 | 2.03 | 0.03 | 645.85 |
| psi(canopy cover + bamboo cover + shrub cover), p(time from sunrise) | 6 | 657.85 | 2.03 | 0.03 | 645.85 |
| psi(distance to PA), p(time from sunrise + shrub cover) | 5 | 657.87 | 2.05 | 0.03 | 647.87 |
| psi(shrub cover), p(time from sunrise) | 4 | 657.89 | 2.08 | 0.03 | 649.89 |
| psi(stand basal area), p(time from sunrise) | 4 | 658.1 | 2.28 | 0.03 | 650.1 |
| psi(.), p(time from sunrise + shrub cover) | 4 | 658.14 | 2.32 | 0.03 | 650.14 |
| psi(.), p(time from sunrise + canopy cover) | 4 | 658.14 | 2.33 | 0.03 | 650.14 |
| psi(distance to PA), p(time from sunrise + canopy cover) | 5 | 658.23 | 2.41 | 0.03 | 648.23 |
| psi(bamboo cover), p(time from sunrise) | 4 | 658.6 | 2.78 | 0.02 | 650.6 |
| psi(canopy cover + bamboo cover + shrub cover + distance to PA), p(time from sunrise + shrub cover) | 8 | 658.69 | 2.87 | 0.02 | 642.69 |
| psi(canopy cover + shrub cover), p(time from sunrise + shrub cover) | 6 | 659.12 | 3.30 | 0.02 | 647.12 |
| psi(canopy cover + bamboo cover + shrub cover), p(time from sunrise + shrub cover) | 7 | 659.12 | 3.30 | 0.02 | 645.12 |
| psi(shrub cover), p(time from sunrise + shrub cover) | 5 | 659.33 | 3.51 | 0.02 | 649.33 |
| psi(canopy cover + bamboo cover + shrub cover + distance to PA), p(time from sunrise + canopy cover) | 8 | 659.44 | 3.62 | 0.01 | 643.44 |
| psi(shrub cover), p(time from sunrise + canopy cover) | 5 | 659.54 | 3.73 | 0.01 | 649.54 |
| psi(stand basal area), p(time from sunrise + shrub cover) | 5 | 659.56 | 3.74 | 0.01 | 649.56 |
| psi(stand basal + shrub cover), p(time from sunrise) | 5 | 659.64 | 3.83 | 0.01 | 649.64 |
| psi(bamboo cover + shrub cover), p(time from sunrise) | 5 | 659.65 | 3.83 | 0.01 | 649.65 |
| psi(canopy cover + shrub cover), p(time from sunrise + canopy cover) | 6 | 659.72 | 3.90 | 0.01 | 647.72 |
| psi(stand basal area), p(time from sunrise + canopy cover) | 5 | 659.8 | 3.98 | 0.01 | 649.8 |
| psi(canopy cover + bamboo cover + shrub cover), p(time from sunrise + canopy cover) | 7 | 659.81 | 3.99 | 0.01 | 645.81 |
| psi(bamboo cover), p(time from sunrise + canopy cover) | 5 | 660.11 | 4.30 | 0.01 | 650.11 |
| psi(bamboo cover), p(time from sunrise + shrub cover) | 5 | 660.12 | 4.31 | 0.01 | 650.12 |
| psi(stand basal area + shrub cover), p(time from sunrise + shrub cover) | 6 | 661.04 | 5.23 | 0.01 | 649.04 |
| psi(bamboo cover + shrub cover), p(time from sunrise + shrub cover) | 6 | 661.08 | 5.26 | 0.01 | 649.08 |
| psi(bamboo cover + shrub cover), p(time from sunrise + canopy cover) | 6 | 661.27 | 5.45 | 0.01 | 649.27 |
| psi(stand basal area + shrub cover), p(time from sunrise + canopy cover) | 6 | 661.38 | 5.56 | 0.01 | 649.38 |

Large understory gleaning insectivores

| Model | **nPar** | **AIC** | **ΔAIC** | **AIC Weight** | **2negLL** |
| --- | --- | --- | --- | --- | --- |
| **psi(.), p(time from sunrise)** | **3** | **492.85** | **0.00** | **0.12** | **486.85** |
| psi(canopy cover), p(time from sunrise) | 4 | 493.96 | 1.12 | 0.07 | 485.96 |
| psi(bamboo cover), p(time from sunrise) | 4 | 494.11 | 1.26 | 0.06 | 486.11 |
| psi(canopy cover + shrub cover), p(time from sunrise) | 5 | 494.37 | 1.53 | 0.06 | 484.37 |
| psi(stand basal area), p(time from sunrise) | 4 | 494.53 | 1.68 | 0.05 | 486.53 |
| psi(.), p(time from sunrise + canopy cover) | 4 | 494.69 | 1.84 | 0.05 | 486.69 |
| psi(distance to PA), p(time from sunrise) | 4 | 494.74 | 1.89 | 0.05 | 486.74 |
| psi(.), p(time from sunrise + shrub cover) | 4 | 494.84 | 2.00 | 0.04 | 486.84 |
| psi(shrub cover), p(time from sunrise) | 4 | 494.85 | 2.00 | 0.04 | 486.85 |
| psi(canopy cover + bamboo cover), p(time from sunrise) | 5 | 495.65 | 2.80 | 0.03 | 485.65 |
| psi(canopy cover), p(time from sunrise + canopy cover) | 5 | 495.83 | 2.98 | 0.03 | 485.83 |
| psi(canopy cover), p(time from sunrise + shrub cover) | 5 | 495.84 | 2.99 | 0.03 | 485.84 |
| psi(bamboo cover), p(time from sunrise shrub cover) | 5 | 496.03 | 3.19 | 0.02 | 486.03 |
| psi(canopy cover + shrub cover), p(time frm sunrise + canopy cover) | 6 | 496.08 | 3.23 | 0.02 | 484.08 |
| psi(bamboo cover + shrub cover), p(time from sunrise) | 5 | 496.1 | 3.25 | 0.02 | 486.1 |
| psi(bamboo cover), p(time from sunrise + canopy cover) | 5 | 496.1 | 3.25 | 0.02 | 486.1 |
| psi(stand basal area), p(time from sunrise + canopy cover) | 5 | 496.15 | 3.30 | 0.02 | 486.15 |
| psi(canopy cover + bamboo cover + shrub cover), p(time from sunrise) | 6 | 496.16 | 3.32 | 0.02 | 484.16 |
| psi(canopy cover + shrub cover), p(time from sunrise + shrub cover) | 6 | 496.28 | 3.43 | 0.02 | 484.28 |
| psi(stand basal area + shrub cover), p(time from sunrise) | 5 | 496.42 | 3.57 | 0.02 | 486.42 |
| psi(stand basal area), p(time from sunrise + shrub cover) | 5 | 496.53 | 3.68 | 0.02 | 486.53 |
| psi(distance to PA), p(time from sunrise + canopy cover) | 5 | 496.54 | 3.69 | 0.02 | 486.54 |
| psi(shrub cover), p(time from sunrise + canopy cover) | 5 | 496.68 | 3.83 | 0.02 | 486.68 |
| psi(distance to PA), p(time from sunrise + shrub cover) | 5 | 496.74 | 3.89 | 0.02 | 486.74 |
| psi(shrub cover), p(time from sunrise + shrub cover) | 5 | 496.83 | 3.98 | 0.02 | 486.83 |
| psi(canopy cover + bamboo cover + shrub cover + distance to PA), p(time from sunrise) | 7 | 497.32 | 4.47 | 0.01 | 483.32 |
| psi(canopy cover + bamboo cover), p(time from sunrise + canopy cover) | 6 | 497.42 | 4.57 | 0.01 | 485.42 |
| psi(canopy cover + bamboo cover), p(time from sunrise + shrub cover) | 6 | 497.43 | 4.59 | 0.01 | 485.43 |
| psi(canopy cover + bamboo cover + shrub cover), p(time from sunrise + canopy cover) | 7 | 497.78 | 4.93 | 0.01 | 483.78 |
| psi(bamboo cover + shrub cover), p(time from sunrise + shrub cover) | 6 | 498.02 | 5.18 | 0.01 | 486.02 |
| psi(bamboo cover + shrub cover), p(time from sunrise + canopy cover) | 6 | 498.09 | 5.24 | 0.01 | 486.09 |
| psi(stand basal area + shrub cover), p(time from sunrise + canopy cover) | 6 | 498.1 | 5.25 | 0.01 | 486.1 |
| psi(canopy cover + bamboo cover + shrub cover), p(time from sunrise + shrub cover) | 7 | 498.11 | 5.26 | 0.01 | 484.11 |
| psi(stand basal area + shrub cover), p(time from sunrise + shrub cover) | 6 | 498.36 | 5.51 | 0.01 | 486.36 |
| psi(canopy cover + bamboo cover + shrub cover + distance to PA), p(time from sunrise + canopy cover) | 8 | 499.07 | 6.22 | 0.01 | 483.07 |
| psi(canopy cover + bamboo cover + shrub cover + distance to PA), p(time from sunrise + shrub cover) | 8 | 499.28 | 6.43 | 0.00 | 483.28 |

Large high-canopy sallying insectivores

| **Model** | **nPar** | **AIC** | **ΔAIC** | **AIC Weight** | **2negLL** |
| --- | --- | --- | --- | --- | --- |
| **psi(shrub cover), p(time from sunrise + canopy cover)** | **5** | **642.09** | **0.00** | **0.19** | **632.09** |
| **psi(shrub cover), p(canopy cover)** | **4** | **643.06** | **0.97** | **0.12** | **635.06** |
| psi(stand basal area + shrub cover), p(time from sunrise + canopy cover) | 6 | 643.5 | 1.42 | 0.09 | 631.5 |
| **psi(shrub cover), p(time from sunrise + canopy cover + shrub cover)** | **6** | **643.54** | **1.45** | **0.09** | **631.54** |
| psi(bamboo cover + shrub cover), p(time from sunrise + canopy cover) | 6 | 644.04 | 1.95 | 0.07 | 632.04 |
| psi(canopy cover + shrub cover), p(time from sunrise + canopy cover) | 6 | 644.08 | 1.99 | 0.07 | 632.08 |
| psi(stand basal area + shrub cover), p(canopy cover) | 5 | 644.51 | 2.42 | 0.06 | 634.51 |
| psi(bamboo cover + shrub cover), p(canopy cover) | 5 | 644.74 | 2.65 | 0.05 | 634.74 |
| psi(stand basal area + shrub cover), p(time from sunrise + canopy cover + shrub cover) | 7 | 644.92 | 2.83 | 0.05 | 630.92 |
| psi(canopy cover + shrub cover), p(canopy cover) | 5 | 645.01 | 2.93 | 0.04 | 635.01 |
| psi(bamboo cover + shrub cover), p(time from sunrise + canopy cover + shrub cover) | 7 | 645.51 | 3.42 | 0.03 | 631.51 |
| psi(canopy cover + shrub cover), p(time from sunrise + canopy cover + shrub cover) | 7 | 645.53 | 3.44 | 0.03 | 631.53 |
| psi(canopy cover + bamboo cover + shrub cover), p(time from sunrise + canopy cover) | 7 | 646.04 | 3.95 | 0.03 | 632.04 |
| psi(canopy cover + bamboo cover + shrub cover), p(canopy cover) | 6 | 646.74 | 4.65 | 0.02 | 634.74 |
| psi(canopy cover + bamboo cover + shrub cover), p(time from sunrise + canopy cover + shrub cover) | 8 | 647.51 | 5.42 | 0.01 | 631.51 |
| psi(canopy cover + bamboo cover + shrub cover + distance to PA), p(time from sunrise + canopy cover) | 8 | 647.86 | 5.77 | 0.01 | 631.86 |
| psi(canopy cover + bamboo cover + shrub cover + distance to PA), p(canopy cover) | 7 | 648.54 | 6.46 | 0.01 | 634.54 |
| psi(canopy cover + bamboo cover + shrub cover + distance to PA), p(time from sunrise + canopy cover + shrub cover) | 9 | 649.27 | 7.18 | 0.01 | 631.27 |
| psi(.), p(time from sunrise + canopy cover) | 4 | 650.09 | 8.00 | 0.00 | 642.09 |
| psi(canopy cover), p(time from sunrise + canopy cover) | 5 | 650.15 | 8.06 | 0.00 | 640.15 |
| psi(distance to PA), p(time from sunrise + canopy cover) | 5 | 650.76 | 8.67 | 0.00 | 640.76 |
| psi(.), p(canopy cover) | 3 | 651.08 | 8.99 | 0.00 | 645.08 |
| psi(canopy cover), p(canopy cover) | 4 | 651.37 | 9.28 | 0.00 | 643.37 |
| psi(.), p(time from sunrise + canopy cover + shrub cover) | 5 | 651.64 | 9.56 | 0.00 | 641.64 |
| psi(distance to PA), p(canopy cover) | 4 | 651.73 | 9.64 | 0.00 | 643.73 |
| psi(bamboo cover), p(time from sunrise + canopy cover) | 5 | 651.94 | 9.85 | 0.00 | 641.94 |
| psi(stand basal area), p(time from sunrise + canopy cover) | 5 | 652.02 | 9.93 | 0.00 | 642.02 |
| psi(canopy cover + bamboo cover), p(time from sunrise + canopy cover) | 6 | 652.12 | 10.03 | 0.00 | 640.12 |
| psi(canopy cover), p(time from sunrise + canopy cover + shrub cover) | 6 | 652.12 | 10.04 | 0.00 | 640.12 |
| psi(distance to PA), p(time from sunrise + canopy cover + shrub cover) | 6 | 652.57 | 10.48 | 0.00 | 640.57 |
| psi(stand basal area), p(canopy cover) | 4 | 652.98 | 10.89 | 0.00 | 644.98 |
| psi(bamboo cover), p(canopy cover) | 4 | 653.06 | 10.98 | 0.00 | 645.06 |
| psi(canopy cover + bamboo cover), p(canopy cover) | 5 | 653.15 | 11.07 | 0.00 | 643.15 |
| psi(bamboo cover), p(time from sunrise + canopy cover + shrub cover) | 6 | 653.61 | 11.52 | 0.00 | 641.61 |
| psi(stand basal area), p(time from sunrise + canopy cover + shrub cover) | 6 | 653.64 | 11.55 | 0.00 | 641.64 |
| psi(canopy cover + bamboo cover), p(time from sunrise + canopy cover + shrub cover) | 7 | 654.08 | 12.00 | 0.00 | 640.08 |

Small mid-canopy gleaning insectivores

| **Model** | **nPar** | **AIC** | **ΔAIC** | **AIC Weight** | **2negLL** |
| --- | --- | --- | --- | --- | --- |
| **psi(distance to PA), p(time from sunrise + shrub cover)** | **5** | **650.55** | **0.00** | **0.19** | **640.55** |
| **psi(.), p(time from sunrise)** | **3** | **651.48** | **0.93** | **0.12** | **645.48** |
| **psi(.), p(time from sunrise + shrub cover)** | **4** | **652.46** | **1.91** | **0.07** | **644.46** |
| psi(shrub cover), p(time from sunrise) | 4 | 652.56 | 2.02 | 0.07 | 644.56 |
| psi(canopy cover), p(time from sunrise) | 4 | 653.34 | 2.80 | 0.05 | 645.34 |
| psi(stand basal area), p(time from sunrise) | 4 | 653.35 | 2.81 | 0.05 | 645.35 |
| psi(bamboo cover), p(time from sunrise) | 4 | 653.36 | 2.81 | 0.05 | 645.36 |
| psi(shrub cover), p(time from sunrise + shrub cover) | 5 | 653.41 | 2.87 | 0.05 | 643.41 |
| psi(bamboo cover + shrub cover), p(time from sunrise) | 5 | 654.03 | 3.48 | 0.03 | 644.03 |
| psi(canopy cover + bamboo cover + shrub cover + distance to PA), p(time from sunrise) | 7 | 654.07 | 3.53 | 0.03 | 640.07 |
| psi(canopy cover + shrub cover), p(time from sunrise) | 5 | 654.27 | 3.72 | 0.03 | 644.27 |
| psi(bamboo cover), p(time from sunrise + shrub cover) | 5 | 654.3 | 3.75 | 0.03 | 644.3 |
| psi(stand basal area), p(time from sunrise + shrub cover) | 5 | 654.32 | 3.77 | 0.03 | 644.32 |
| psi(canopy cover), p(time from sunrise + shrub cover) | 5 | 654.35 | 3.80 | 0.03 | 644.35 |
| psi(stand basal area + shrub cover), p(time from sunrise) | 5 | 654.56 | 4.01 | 0.03 | 644.56 |
| psi(canopy cover + bamboo cover + shrub cover + distance to PA), p(time from sunrise + shrub cover) | 8 | 654.73 | 4.18 | 0.02 | 638.73 |
| psi(bamboo cover + shrub cover), p(time from sunrise + shrub cover) | 6 | 654.81 | 4.26 | 0.02 | 642.81 |
| psi(canopy cover + bamboo cover), p(time from sunrise) | 5 | 654.95 | 4.40 | 0.02 | 644.95 |
| psi(canopy cover + shrub cover), p(time from sunrise + shrub cover) | 6 | 655.07 | 4.52 | 0.02 | 643.07 |
| psi(stand basal area + shrub cover), p(time from sunrise + shrub cover) | 6 | 655.41 | 4.86 | 0.02 | 643.41 |
| psi(canopy cover + bamboo cover), p(time from sunrise + shrub cover) | 6 | 655.92 | 5.38 | 0.01 | 643.92 |
| psi(canopy cover + bamboo cover + shrub cover), p(time from sunrise) | 6 | 655.93 | 5.39 | 0.01 | 643.93 |
| psi(canopy cover + bamboo cover + shrub cover), p(time from sunrise + shrub cover) | 7 | 656.7 | 6.15 | 0.01 | 642.7 |

Small understory gleaning insectivores

| **Model** | **nPar** | **AIC** | **ΔAIC** | **AIC Weight** | **2negLL** |
| --- | --- | --- | --- | --- | --- |
| **psi(canopy cover), p(time from sunrise + canopy cover + shrub cover)** | **6** | **615.34** | **0.00** | **0.26** | **603.34** |
| **psi(stand basal area), p(time from sunrise + canopy cover + shrub cover)** | **6** | **616.67** | **1.32** | **0.13** | **604.67** |
| **psi(.), p(time from sunrise + canopy cover + shrub cover)** | **5** | **616.96** | **1.62** | **0.12** | **606.96** |
| psi(canopy cover + bamboo cover), p(time from sunrise + canopy cover + shrub cover) | 7 | 617.05 | 1.71 | 0.11 | 603.05 |
| psi(canopy cover + shrub cover), p(time from sunrise + canopy cover + shrub cover) | 7 | 617.34 | 2.00 | 0.10 | 603.34 |
| psi(stand basal area + shrub cover), p(time from sunrise + canopy cover + shrub cover) | 7 | 618.24 | 2.89 | 0.06 | 604.24 |
| psi(shrub cover), p(time from sunrise + canopy cover + shrub cover) | 6 | 618.33 | 2.99 | 0.06 | 606.33 |
| psi(bamboo cover), psi(time from sunrise + canopy cover + shrub cover) | 6 | 618.88 | 3.54 | 0.04 | 606.88 |
| psi(distance to PA), p(time from sunrise + canopy cover + shrub cover) | 6 | 618.96 | 3.62 | 0.04 | 606.96 |
| psi(canopy cover + bamboo cover + shrub cover), p(time from sunrise + canopy cover + shrub cover) | 8 | 619.05 | 3.71 | 0.04 | 603.05 |
| psi(bamboo cover + shrub cover), p(time from sunrise + canopy cover + shrub cover) | 7 | 620.3 | 4.95 | 0.02 | 606.3 |
| psi(canopy cover + bamboo cover + shrub cover + distance to PA), p(time from sunrise + canopy cover + shrub cover) | 9 | 620.85 | 5.51 | 0.02 | 602.85 |

Small mid-canopy sallying insectivores

| **Model** | **nPar** | **AIC** | **ΔAIC** | **AIC Weight** | **2negLL** |
| --- | --- | --- | --- | --- | --- |
| **psi(bamboo cover + shrub cover), p(canopy cover + shrub cover)** | **6** | **609.25** | **0.00** | **0.42** | **597.25** |
| **psi(bamboo cover), p(canopy cover + shrub cover)** | **5** | **610.71** | **1.46** | **0.20** | **600.71** |
| psi(canopy cover + bamboo cover), p(canopy cover + shrub cover) | 6 | 611.84 | 2.59 | 0.12 | 599.84 |
| psi(shrub cover), p(canopy cover + shrub cover) | 5 | 612.58 | 3.33 | 0.08 | 602.58 |
| psi(canopy cover), p(canopy cover + shrub cover) | 5 | 613.94 | 4.70 | 0.04 | 603.94 |
| psi(stand basal area + shrub cover), p(canopy cover + shrub cover) | 6 | 614.35 | 5.10 | 0.03 | 602.35 |
| psi(canopy cover + shrub cover), p(canopy cover + shrub cover) | 6 | 614.38 | 5.13 | 0.03 | 602.38 |
| psi(.), p(canopy cover + shrub cover) | 4 | 614.43 | 5.18 | 0.03 | 606.43 |
| psi(stand basal area), p(canopy cover + shrub cover) | 5 | 615.14 | 5.89 | 0.02 | 605.14 |
| psi(distance to PA), p(canopy cover + shrub cover) | 5 | 615.96 | 6.72 | 0.01 | 605.96 |

Large woodpeckers

| **Model** | **nPar** | **AIC** | **ΔAIC** | **AIC Weight** | **2negLL** |
| --- | --- | --- | --- | --- | --- |
| **psi(shrub cover), p(time from sunrise + canopy cover)** | **5** | **457.2** | **0.00** | **0.12** | **447.2** |
| **psi(.), p(time from sunrise + canopy cover)** | **4** | **457.99** | **0.80** | **0.08** | **449.99** |
| psi(stand basal area + shrub cover), p(time from sunrise + canopy cover) | 6 | 458.3 | 1.10 | 0.07 | 446.3 |
| psi(shrub cover), p(time from sunrise + canopy cover + shrub cover) | 6 | 458.31 | 1.11 | 0.07 | 446.31 |
| psi(distance to PA), p(time from sunrise + canopy cover) | 5 | 458.93 | 1.73 | 0.05 | 448.93 |
| psi(canopy cover + shrub cover), p(time from sunrise + canopy cover) | 6 | 458.94 | 1.74 | 0.05 | 446.94 |
| psi(bamboo cover + shrub cover), p(time from sunrise + canopy cover) | 6 | 459.04 | 1.84 | 0.05 | 447.04 |
| psi(canopy cover + shrub cover), p(time from sunrise + canopy cover + shrub cover) | 7 | 459.17 | 1.97 | 0.05 | 445.17 |
| psi(stand basal area), p(time from sunrise + canopy cover) | 5 | 459.74 | 2.54 | 0.03 | 449.74 |
| psi(.), p(time from sunrise + canopy cover + shrub cover) | 5 | 459.77 | 2.58 | 0.03 | 449.77 |
| psi(stand basal area + shrub cover), p(time from sunrise + canopy cover + shrub cover) | 7 | 459.8 | 2.61 | 0.03 | 445.8 |
| psi(canopy cover), p(time from sunrise + canopy cover) | 5 | 459.88 | 2.68 | 0.03 | 449.88 |
| psi(bamboo cover), p(time from sunrise + canopy cover) | 5 | 459.98 | 2.79 | 0.03 | 449.98 |
| psi(canopy cover + bamboo cover + shrub cover), p(time from sunrise + canopy cover + shrub cover) | 8 | 459.99 | 2.80 | 0.03 | 443.99 |
| psi(canopy cover + bamboo cover + shrub cover), p(time from sunrise + canopy cover) | 7 | 460.02 | 2.82 | 0.03 | 446.02 |
| psi(bamboo cover + shrub cover), p(time from sunrise + canopy cover + shrub cover) | 7 | 460.18 | 2.98 | 0.03 | 446.18 |
| psi(shrub cover), p(canopy cover + shrub cover) | 5 | 460.81 | 3.61 | 0.02 | 450.81 |
| psi(distance to PA), p(time from sunrise + canopy cover + shrub cover) | 6 | 460.88 | 3.68 | 0.02 | 448.88 |
| psi(canopy cover + shrub cover), p(canopy cover + shrub cover) | 6 | 460.97 | 3.78 | 0.02 | 448.97 |
| psi(canopy cover + bamboo cover + shrub cover), p(canopy cover + shrub cover) | 7 | 461.45 | 4.26 | 0.01 | 447.45 |
| psi(stand basal area), p(time from sunrise + canopy cover + shrub cover) | 6 | 461.62 | 4.42 | 0.01 | 449.62 |
| psi(canopy cover), p(time from sunrise + canopy cover + shrub cover) | 6 | 461.69 | 4.50 | 0.01 | 449.69 |
| psi(bamboo cover), p(time from sunrise + canopy cover + shrub cover) | 6 | 461.75 | 4.56 | 0.01 | 449.75 |
| psi(canopy cover + bamboo cover), p(time from sunrise + canopy cover) | 6 | 461.87 | 4.68 | 0.01 | 449.87 |
| psi(canopy cover + bamboo cover + shrub cover + distance to PA), p(time from sunrise + canopy cover) | 8 | 461.9 | 4.71 | 0.01 | 445.9 |
| psi(canopy cover + bamboo cover + shrub cover + distance to PA), p(time from sunrise + canopy cover + shrub cover) | 9 | 461.91 | 4.72 | 0.01 | 443.91 |
| psi(.), p(canopy cover + shrub cover) | 4 | 462.56 | 5.37 | 0.01 | 454.56 |
| psi(bamboo cover + shrub cover), p(canopy cover, shrub cover) | 6 | 462.63 | 5.44 | 0.01 | 450.63 |
| psi(stand basal area + shrub cover), p(canopy cover + shrub cover) | 6 | 462.77 | 5.58 | 0.01 | 450.77 |
| psi(canopy cover + bamboo cover + shrub cover + distance to PA), p(canopy cover + shrub cover) | 8 | 463.44 | 6.24 | 0.01 | 447.44 |
| psi(distance to PA), p(canopy cover + shrub cover) | 5 | 463.49 | 6.29 | 0.01 | 453.49 |
| psi(canopy cover + bamboo cover), p(time from sunrise + canopy cover + shrub cover) | 7 | 463.69 | 6.50 | 0.00 | 449.69 |
| psi(bamboo cover), p(canopy cover + shrub cover) | 5 | 464.55 | 7.36 | 0.00 | 454.55 |
| psi(canopy cover), p(canopy cover + shrub cover) | 5 | 464.56 | 7.37 | 0.00 | 454.56 |
| psi(stand basal area), p(canopy cover + shrub cover) | 5 | 464.56 | 7.37 | 0.00 | 454.56 |
| psi(canopy cover + bamboo cover), p(canopy cover + shrub cover) | 6 | 466.55 | 9.36 | 0.00 | 454.55 |
