# Supplementary material for: “In a tree by the brook, there’s a songbird who sings”: Woodlands in an agricultural matrix maintain functionality of a wintering bird community
Source: PLoS One. 2018 Aug 2;13(8):e0201657. doi: 10.1371/journal.pone.0201657 (PMC6072076; doi:10.1371/journal.pone.0201657)
Supplement: S3 File — Standard errors are shown in parentheses. Model selection was conducted using AIC, while removing all uninformative models. Chosen models for inference are highlighted in bold. Covariate coefficients for site-use are reported from models marked ‘*’. (DOCX) [file pone.0201657.s003.docx]

**S3 File.** **Covariate coefficients of site-use for all the models of each guild.** Standard errors are shown in parentheses. Model selection was conducted using AIC, while removing all uninformative models. Chosen models for inference are highlighted in bold. Covariate coefficients for site-use are reported from models marked ‘*’.

Nectarivores

| **Model** | **Intercept** | **Canopy cover** | **Bamboo cover** | **Stand basal area** | **Shrub cover** | **Distance to PA** |
| --- | --- | --- | --- | --- | --- | --- |
| **psi(shrub cover), p(.)*** | **5.46 (2.18)** | **-** | **-** | **-** | **3 (1.9)** | **-** |
| psi(canopy cover + shrub cover), p(.) | 6.75 (2.7) | -1.71 (1.66) | - | - | 2.77 (1.77) | - |
| psi(stand basal area + shrub cover), p(.) | 6.72 (2.95) | - | - | -0.88 (0.84) | 3.66 (2.18) | - |
| psi(shrub cover), p(canopy cover) | 5.44 (2.1) | - | - | - | 3.02 (1.84) | - |
| psi(bamboo cover + shrub cover), p(.) | 5.36 (2.14) | - | -0.21 (0.52) | - | 2.83 (1.91) | - |
| psi(shrub cover), p(shrub cover) | 5.44 (2.14) | - | - | - | 3.01 (1.86) | - |
| psi(shrub cover), p(time from sunrise) | 5.48 (2.21) | - | - | - | 3.01 (1.91) | - |
| psi(canopy cover), p(.) | 4.87 (2.29) | -2.03 (1.89) | - | - | - | - |
| psi(canopy cover + shrub cover),p (canopy cover) | 6.74 (2.67) | -1.74 (1.63) | - | - | 2.78 (1.75) | - |
| psi(canopy cover + shrub cover), p(shrub cover) | 6.74 (2.67) | -1.72 (1.63) | - | - | 2.78 (1.75) | - |
| psi(canopy cover + bamboo cover + shrub cover), p(.) | 7.02 (2.92) | -2.04 (2.02) | 0.19 (0.61) | - | 2.83 (1.8) | - |
| psi(canopy cover + shrub cover), p(time from sunrise) | 6.74 (2.7) | -1.71 (1.66) | - | - | 2.77 (1.77) | - |
| psi(stand basal area + shrub cover), p(canopy cover) | 6.61 (2.83) | - | - | -0.84 (0.81) | 3.62 (2.12) | - |
| psi(stand basal area + shrub cover), p(shrub cover) | 6.64 (2.87) | - | - | -0.85 (0.82) | 3.63 (2.14) | - |
| psi(.), p(.) | 3.64 (0.92) | - | - | - | - | - |
| psi(stand basal area + shrub cover), p(time from sunrise) | 6.73 (2.96) | - | - | -0.88 (0.84) | 3.66 (2.19) | - |
| psi(distance to PA), p(.) | 3.87 (1.15) | - | - | - | - | 1.11 (1.25) |
| psi(bamboo cover + shrub cover), p(canopy cover) | 5.35 (2.06) | - | -0.22 (0.5) | - | 2.86 (1.84) | - |
| psi(bamboo cover), p(.) | 3.79 (1.07) | - | -0.68 (0.57) | - | - | - |
| psi(bamboo cover + shrub cover), p(shrub cover) | 5.35 (2.1) | - | -0.21 (0.5) | - | 2.84 (1.87) | - |
| psi(bamboo cover + shrub cover), p(time from sunrise) | 5.38 (2.16) | - | -0.21 (0.52) | - | 2.84 (1.92) | - |
| psi(canopy cover), p(canopy cover) | 4.86 (2.18) | -2.05 (1.79) | - | - | - | - |
| psi(stand basal area), p(.) | 3.85 (1.14) | - | - | -0.59 (0.6) | - | - |
| psi(canopy cover), p(shrub cover) | 4.82 (2.21) | -2.02 (1.82) | - | - | - | - |
| psi(canopy cover + bamboo cover), p(.) | 5.13 (2.66) | -2.37 (2.46) | 0.18 (0.81) | - | - | - |
| psi(canopy cover + bamboo cover + shrub cover), p(canopy cover) | 7 (2.88) | -2.04 (1.98) | 0.18 (0.58) | - | 2.83 (1.78) | - |
| psi(canopy cover), p(time from sunrise) | 4.87 (2.29) | -2.02 (1.89) | - | - | - | - |
| psi(canopy cover + bamboo cover + shrub cover + distance to PA), p(.) | 7.57 (3.46) | -2.13 (2.07) | 0.31 (0.67) | - | 3.1 (2.05) | 0.42 (1.01) |
| psi(canopy cover + bamboo cover + shrub cover), p(shrub cover) | 7 (2.89) | -2.03 (1.98) | 0.18 (0.59) | - | 2.83 (1.79) | - |
| psi(canopy cover + bamboo cover + shrub cover), p(time from sunrise) | 7.02 (2.92) | -2.03 (2.02) | 0.19 (0.61) | - | 2.83 (1.81) | - |
| psi(.), p(time from sunrise) | 3.72 (1.02) | - | - | - | - | - |
| psi(.), p(canopy cover) | 3.63 (0.94) | - | - | - | - | - |
| psi(.), p(shrub cover) | 3.64 (0.95) | - | - | - | - | - |
| psi(distance to PA), p(time from sunrise) | 3.96 (1.26) | - | - | - | - | 1.16 (1.36) |
| psi(distance to PA), p(canopy cover) | 3.85 (1.14) | - | - | - | - | 1.1 (1.24) |
| psi(distance to PA), p(shrub cover) | 3.85 (1.15) | - | - | - | - | 1.1 (1.24) |
| psi(bamboo cover), p(time from sunrise) | 3.87 (1.18) | - | -0.71 (0.61) | - | - | - |
| psi(bamboo cover), p(canopy cover) | 3.77 (1.06) | - | -0.68 (0.57) | - | - | - |
| psi(bamboo cover), p(shrub cover) | 3.78 (1.09) | - | -0.68 (0.57) | - | - | - |
| psi(canopy cover + bamboo cover + shrub cover + distance to PA), p(canopy cover) | 7.53 (3.39) | -2.15 (2.05) | 0.29 (0.64) | - | 3.07 (1.99) | 0.4 (0.98) |
| psi(canopy cover + bamboo cover), p(canopy cover) | 5.08 (2.5) | -2.36 (2.31) | 0.16 (0.75) | - | - | - |
| psi(stand basal area), p(time from sunrise) | 3.88 (1.18) | - | - | -0.58 (0.61) | - | - |
| psi(stand basal area), p(canopy cover) | 3.83 (1.13) | - | - | -0.59 (0.59) | - | - |
| psi(stand basal area), p(shrub cover) | 3.84 (1.16) | - | - | -0.59 (0.59) | - | - |
| psi(canopy cover + bamboo cover), p(shrub cover) | 5.07 (2.55) | -2.34 (2.36) | 0.17 (0.78) | - | - | - |
| psi(canopy cover + bamboo cover + shrub cover + distance to PA), p(shrub cover) | 7.54 (3.41) | -2.12 (2.04) | 0.3 (0.65) | - | 3.1 (2.02) | 0.41 (1) |
| psi(canopy cover + bamboo cover), p(time from sunrise) | 5.12 (2.65) | -2.35 (2.46) | 0.18 (0.83) | - | - | - |
| psi(canopy cover + bamboo cover + shrub cover + distance to PA), p(time from sunrise) | 7.56 (3.46) | -2.13 (2.07) | 0.31 (0.68) | - | 3.09 (2.05) | 0.42 (1.01) |

Granivores

| **Model** | **Intercept** | **Canopy cover** | **Bamboo cover** | **Stand basal area** | **Shrub cover** | **Distance to PA** |
| --- | --- | --- | --- | --- | --- | --- |
| **psi(canopy cover + bamboo cover), p(time from sunrise)** | **-0.03 (0.31)** | **-0.71 (0.32)** | **-0.85 (0.42)** | **-** | **-** | **-** |
| **psi(bamboo cover + shrub cover), p(time from sunrise + canopy cover)** | **0.11 (0.34)** | **-** | **-0.98 (0.41)** | **-** | **0.62 (0.35)** | **-** |
| **psi(bamboo cover + shrub cover), p(time from sunrise)** | **0 (0.32)** | **-** | **-1.04 (0.4)** | **-** | **0.68 (0.35)** | **-** |
| **psi(canopy cover + bamboo cover), p(time from sunrise + canopy cover)** | **0.04 (0.33)** | **-0.61 (0.33)** | **-0.84 (0.42)** | **-** | **-** | **-** |
| **psi(canopy cover + bamboo cover + shrub cover), p(time from sunrise)** | **-0.03 (0.31)** | **-0.47 (0.37)** | **-0.86 (0.42)** | **-** | **0.4 (0.38)** | **-** |
| **psi(canopy cover + bamboo cover + shrub cover), p(time from sunrise + canopy cover)*** | **0.04 (0.33)** | **-0.36 (0.39)** | **-0.85 (0.43)** | **-** | **0.42 (0.39)** | **-** |
| **psi(canopy cover + bamboo cover), p(time from sunrise + shrub cover)** | **0.01 (0.32)** | **-0.69 (0.32)** | **-0.86 (0.42)** | **-** | **-** | **-** |
| **psi(bamboo cover), p(time from sunrise + canopy cover)** | **0.2 (0.37)** | **-** | **-1.14 (0.41)** | **-** | **-** | **-** |
| psi(bamboo cover + shrub cover), p(time from sunrise + shrub cover) | 0.02 (0.32) | - | -1.04 (0.4) | - | 0.64 (0.35) | - |
| psi(canopy cover + bamboo cover + shrub cover), p(time from sunrise + shrub cover) | -0.02 (0.32) | -0.48 (0.37) | -0.86 (0.42) | - | 0.37 (0.38) | - |
| psi(canopy cover + bamboo cover + shrub cover + distance to PA), p(time from sunrise) | -0.02 (0.32) | -0.5 (0.38) | -0.83 (0.42) | - | 0.43 (0.39) | -0.1 (0.3) |
| psi(canopy cover), p(time from sunrise) | 0.08 (0.29) | -0.99 (0.29) | - | - | - | - |
| psi(canopy cover + bamboo cover + shrub cover + distance to PA), p(time from sunrise + canopy cover) | 0.06 (0.34) | -0.38 (0.4) | -0.82 (0.43) | - | 0.46 (0.4) | -0.12 (0.31) |
| psi(bamboo cover), p(time from sunrise) | 0.01 (0.31) | - | -1.2 (0.39) | - | - | - |
| psi(canopy cover), p(time from sunrise + canopy cover) | 0.16 (0.31) | -0.87 (0.31) | - | - | - | - |
| psi(canopy cover + shrub cover), p(time from sunrise) | 0.07 (0.29) | -0.77 (0.34) | - | - | 0.37 (0.35) | - |
| psi(canopy cover + shrub cover), p(time from sunrise + canopy cover) | 0.15 (0.32) | -0.64 (0.36) | - | - | 0.39 (0.36) | - |
| psi(bamboo cover), p(time from sunrise + shrub cover) | 0.08 (0.33) | - | -1.22 (0.41) | - | - | - |
| psi(canopy cover + bamboo cover + shrub cover + distance to PA), p(time from sunrise + shrub cover) | 0.01 (0.33) | -0.51 (0.39) | -0.83 (0.42) | - | 0.4 (0.39) | -0.12 (0.31) |
| psi(canopy cover), p(time from sunrise + shrub cover) | 0.11 (0.3) | -0.97 (0.29) | - | - | - | - |
| psi(shrub cover), p(time from sunrise + canopy cover) | 0.37 (0.35) | - | - | - | 0.75 (0.34) | - |
| psi(canopy cover + shrub cover), p(time from sunrise + shrub cover) | 0.09 (0.3) | -0.78 (0.34) | - | - | 0.34 (0.36) | - |
| psi(stand basal area + shrub cover), p(time from sunrise + canopy cover) | 0.59 (0.58) | - | - | 0.56 (0.75) | 1 (0.5) | - |
| psi(shrub cover), p(time from sunrise) | 0.16 (0.29) | - | - | - | 0.85 (0.33) | - |
| psi(.), p(time from sunrise + canopy cover) | 0.63 (0.41) | - | - | - | - | - |
| psi(shrub cover), p(time from sunrise + shrub cover) | 0.18 (0.3) | - | - | - | 0.81 (0.33) | - |
| psi(stand basal area + shrub cover), p(time from sunrise) | 0.18 (0.3) | - | - | 0.15 (0.3) | 0.93 (0.38) | - |
| psi(distance to PA), p(time from sunrise + canopy cover) | 0.59 (0.41) | - | - | - | - | 0.1 (0.32) |
| psi(stand basal area + shrub cover), p(time from sunrise + shrub cover) | 0.2 (0.31) | - | - | 0.15 (0.31) | 0.89 (0.38) | - |
| psi(stand basal area), p(time from sunrise + canopy cover) | 0.65 (0.47) | - | - | 0.04 (0.38) | - | - |
| psi(.), p(time from sunrise) | 0.18 (0.28) | - | - | - | - | - |
| psi(.), p(time from sunrise + shrub cover) | 0.31 (0.32) | - | - | - | - | - |
| psi(distance to PA), p(time from sunrise) | 0.16 (0.28) | - | - | - | - | 0.25 (0.26) |
| psi(stand basal area), p(time from sunrise) | 0.17 (0.28) | - | - | -0.23 (0.25) | - | - |
| psi(stand basal area), p(time from sunrise + shrub cover) | 0.28 (0.32) | - | - | -0.21 (0.26) | - | - |
| psi(distance to PA), p(time from sunrise + shrub cover) | 0.27 (0.31) | - | - | - | - | 0.21 (0.27) |

Omnivores

| **Model** | **Intercept** | **Canopy cover** | **Bamboo cover** | **Stand basal area** | **Shrub cover** | **Distance to PA** |
| --- | --- | --- | --- | --- | --- | --- |
| **psi(bamboo cover), p(shrub cover)** | **1.46 (0.64)** | **-** | **-0.86 (0.4)** | **-** | **-** | **-** |
| **psi(bamboo cover), p(canopy cover + shrub cover)** | **1.51 (0.68)** | **-** | **-0.8 (0.41)** | **-** | **-** | **-** |
| **psi(bamboo cover), p(time from sunrise + shrub cover)** | **1.39 (0.6)** | **-** | **-0.8 (0.38)** | **-** | **-** | **-** |
| **psi(canopy cover), p(shrub cover)** | **3.73 (2.52)** | **-2.78 (2.04)** | **-** | **-** | **-** | **-** |
| **psi(canopy cover), p(time from sunrise + shrub cover)** | **4.12 (2.78)** | **-3.07 (2.23)** | **-** | **-** | **-** | **-** |
| **psi(canopy cover + bamboo cover), p(shrub cover)*** | **1.81 (1.05)** | **-0.7 (1.17)** | **-0.65 (0.47)** | **-** | **-** | **-** |
| psi(bamboo cover + shrub cover), p(shrub cover) | 1.5 (0.79) | - | -0.86 (0.4) | - | 0.08 (0.91) | - |
| psi(canopy cover + bamboo cover), p(canopy cover + shrub cover) | 1.84 (1.24) | 1.07 (1.43) | -1.3 (0.98) | - | - | - |
| psi(canopy cover + bamboo cover), p(time from sunrise + shrub cover) | 1.77 (1.15) | -0.69 (1.26) | -0.61 (0.47) | - | - | - |
| psi(canopy cover + shrub cover), p(shrub cover) | 4.1 (2.41) | -2.73 (1.8) | - | - | 0.59 (1.12) | - |
| psi(canopy cover), p(canopy cover + shrub cover) | 3.9 (2.82) | -2.84 (2.23) | - | - | - | - |
| psi(bamboo cover + shrub cover), p(canopy cover + shrub cover) | 1.53 (0.89) | - | -0.81 (0.42) | - | 0.02 (1) | - |
| psi(.), p(canopy cover + shrub cover) | 1.43 (0.63) | - | - | - | - | - |
| psi(canopy cover + shrub cover), p(time from sunrise + shrub cover) | 4.53 (2.68) | -2.96 (1.97) | - | - | 0.71 (1.23) | - |
| psi(bamboo cover + shrub cover), p(time from sunrise + shrub cover) | 1.47 (0.78) | - | -0.79 (0.39) | - | 0.15 (0.86) | - |
| psi(bamboo cover + shrub cover), p(.) | 1.12 (0.54) | - | -0.88 (0.42) | - | -1.04 (0.48) | - |
| psi(canopy cover + bamboo cover + shrub cover), p(shrub cover) | 1.88 (1.29) | -0.72 (1.22) | -0.63 (0.49) | - | 0.1 (0.84) | - |
| psi(.), p(shrub cover) | 1.26 (0.54) | - | - | - | - | - |
| psi(.), p(time from sunrise + shrub cover) | 1.23 (0.51) | - | - | - | - | - |
| psi(canopy cover + bamboo cover + shrub cover), p(canopy cover + shrub cover) | 1.75 (1.2) | 1.04 (1.3) | -1.31 (0.91) | - | -0.29 (0.88) | - |
| psi(bamboo cover + shrub cover), p(time from sunrise) | 1.07 (0.52) | - | -0.82 (0.41) | - | -0.98 (0.47) | - |
| psi(canopy cover + bamboo cover + shrub cover), p(time from sunrise + shrub cover) | 1.94 (1.57) | -0.76 (1.44) | -0.57 (0.5) | - | 0.18 (0.86) | - |
| psi(stand basal area), p(canopy cover + shrub cover) | 1.54 (0.75) | - | - | 0.45 (0.8) | - | - |
| psi(canopy cover + bamboo cover + shrub cover + distance to PA), p(shrub cover) | 1.37 (1.18) | -0.34 (1) | -0.67 (0.56) | - | -0.6 (1.19) | 0.6 (0.64) |
| psi(shrub cover), p(canopy cover + shrub cover) | 1.37 (0.67) | - | - | - | -0.52 (0.98) | - |
| psi(canopy cover + bamboo cover + shrub cover + distance to PA), p(.) | 1.1 (0.52) | 0.07 (0.56) | -0.88 (0.5) | - | -1.3 (0.6) | 0.6 (0.48) |
| psi(canopy cover + bamboo cover + shrub cover), p(.) | 1.14 (0.55) | 0.1 (0.59) | -0.93 (0.53) | - | -1.01 (0.5) | - |
| psi(canopy cover + bamboo cover + shrub cover + distance to PA), p(time from sunrise + shrub cover) | 1.44 (1.61) | -0.37 (1.2) | -0.61 (0.6) | - | -0.47 (1.22) | 0.62 (0.79) |
| psi(canopy cover + bamboo cover + shrub cover + distance to PA), p(canopy cover + shrub cover) | 1.26 (0.68) | 0.5 (1.01) | -1.01 (0.68) | - | -0.68 (0.75) | 0.43 (0.5) |
| psi(shrub cover), p(shrub cover) | 1.69 (1.06) | - | - | - | 0.66 (1.05) | - |
| psi(shrub cover), p(time from sunrise + shrub cover) | 1.65 (1.03) | - | - | - | 0.64 (1.03) | - |
| psi(shrub cover), p(.) | 1.3 (0.71) | - | - | - | -0.86 (0.49) | - |
| psi(stand basal area), p(shrub cover) | 1.29 (0.56) | - | - | -0.13 (0.42) | - | - |
| psi(stand basal area), p(time from sunrise + shrub cover) | 1.25 (0.53) | - | - | -0.12 (0.42) | - | - |
| psi(canopy cover + bamboo cover + shrub cover + distance to PA), p(time from sunrise) | 1.06 (0.51) | 0.08 (0.55) | -0.83 (0.5) | - | -1.25 (0.59) | 0.59 (0.47) |
| psi(stand basal area + shrub cover), p(canopy cover + shrub cover) | 1.99 (1.22) | - | - | 0.67 (0.9) | 0.79 (1.18) | - |
| psi(shrub cover), p(time from sunrise) | 1.24 (0.66) | - | - | - | -0.82 (0.47) | - |
| psi(canopy cover + bamboo cover + shrub cover), p(time from sunrise) | 1.09 (0.54) | 0.11 (0.58) | -0.88 (0.53) | - | -0.96 (0.48) | - |
| psi(canopy cover + shrub cover), p(.) | 1.15 (0.58) | -0.52 (0.56) | - | - | -1.02 (0.52) | - |
| psi(stand basal area + shrub cover), p(.) | 1.35 (0.75) | - | - | -0.28 (0.43) | -0.99 (0.56) | - |
| psi(canopy cover + shrub cover), p(time from sunrise) | 1.1 (0.54) | -0.42 (0.53) | - | - | -0.94 (0.49) | - |
| psi(stand basal area + shrub cover), p(shrub cover) | 1.79 (1.49) | - | - | 0.11 (0.95) | 0.8 (1.6) | - |
| psi(stand basal area + shrub cover), p(time from sunrise + shrub cover) | 1.73 (1.28) | - | - | 0.1 (0.79) | 0.76 (1.4) | - |
| psi(stand basala area + shrub cover), p(time from sunrise) | 1.26 (0.68) | - | - | -0.25 (0.42) | -0.92 (0.52) | - |
| psi(bamboo cover), p(.) | 1.06 (0.51) | - | -0.56 (0.34) | - | - | - |
| psi(bamboo cover), p(time from sunrise) | 1.03 (0.48) | - | -0.51 (0.34) | - | - | - |
| psi(.), p(time from sunrise) | 1.04 (0.47) | - | - | - | - | - |
| psi(.), p(.) | 1.05 (0.48) | - | - | - | - | - |
| psi(canopy cover + bamboo cover), p(.) | 1.08 (0.53) | 0.54 (0.55) | -0.83 (0.48) | - | - | - |
| psi(canopy cover + bamboo cover), p(time from sunrise) | 1.06 (0.52) | 0.52 (0.54) | -0.78 (0.47) | - | - | - |
| psi(stand basal area), p(time from sunrise) | 1.05 (0.49) | - | - | 0.2 (0.48) | - | - |
| psi(distance to PA), p(time from sunrise) | 1.04 (0.47) | - | - | - | - | 0.15 (0.39) |
| psi(canopy cover), p(time from sunrise) | 1.05 (0.48) | 0.1 (0.43) | - | - | - | - |
| psi(stand basal area), p(.) | 1.08 (0.52) | - | - | 0.22 (0.52) | - | - |
| psi(distance to PA), p(.) | 1.05 (0.49) | - | - | - | - | 0.14 (0.4) |
| psi(canopy cover), p(.) | 1.06 (0.49) | 0.05 (0.44) | - | - | - | - |

Frugivores

| **Model** | **Intercept** | **Canopy cover** | **Bamboo cover** | **Stand basal area** | **Shrub cover** | **Distance to PA** |
| --- | --- | --- | --- | --- | --- | --- |
| **psi(.), p(shrub cover)*** | **3.99 (0.79)** | **-** | **-** | **-** | **-** | **-** |
| **psi(.), p(.)** | **3.95 (0.75)** | **-** | **-** | **-** | **-** | **-** |
| psi(.), p(time from sunrise + shrub cover) | 4.05 (0.84) | - | - | - | - | - |
| psi(.), p(time from sunrise) | 3.97 (0.78) | - | - | - | - | - |
| psi(shrub cover), p(.) | 4.13 (0.88) | - | - | - | -0.55 (0.55) | - |
| psi(bamboo cover), p(.) | 4.24 (1.06) | - | 0.97 (1.49) | - | - | - |
| psi(shrub cover), p(shrub cover) | 4.11 (0.85) | - | - | - | -0.52 (0.56) | - |
| psi(bamboo cover), p(shrub cover) | 4.23 (1.03) | - | 0.91 (1.46) | - | - | - |
| psi(distance to PA), p(shrub cover) | 4.08 (0.85) | - | - | - | - | -0.48 (0.74) |
| psi(.), p(canopy cover) | 3.95 (0.76) | - | - | - | - | - |
| psi(distance to PA), p(.) | 16.16 (13.42) | - | - | - | - | -7.14 (6.72) |
| psi(canopy cover), p(.) | 3.95 (0.76) | 0.1 (0.78) | - | - | - | - |
| psi(stand basal area), p(.) | 3.95 (0.76) | - | - | 0.07 (0.79) | - | - |
| psi(canopy cover), p(shrub cover) | 4 (0.8) | -0.02 (0.88) | - | - | - | - |
| psi(stand basal area), p(shrub cover) | 4 (0.8) | - | - | -0.01 (0.8) | - | - |
| psi(shrub cover), p(time from sunrise + shrub cover) | 4.16 (0.9) | - | - | - | -0.53 (0.58) | - |
| psi(bamboo cover), p(time from sunrise + shrub cover) | 4.27 (1.06) | - | 0.9 (1.49) | - | - | - |
| psi(shrub cover), p(time from sunrise) | 4.2 (0.95) | - | - | - | -0.58 (0.57) | - |
| psi(bamboo cover), p(time from sunrise) | 4.28 (1.11) | - | 1 (1.55) | - | - | - |
| psi(distance to PA), p(time from sunrise + shrub cover) | 4.13 (0.91) | - | - | - | - | -0.5 (0.79) |
| psi(distance to PA), p(time from sunrise) | 4.2 (1.03) | - | - | - | - | -0.61 (0.82) |
| psi(canopy cover), p(time from sunrise + shrub cover) | 4.07 (0.9) | -0.08 (1.05) | - | - | - | - |
| psi(stand basal area), p(time from sunrise + shrub cover) | 4.06 (0.85) | - | - | -0.03 (0.83) | - | - |
| psi(bamboo cover + shrub cover), p(.) | 4.4 (1.22) | - | 0.89 (1.63) | - | -0.46 (0.57) | - |
| psi(bamboo cover + shrub cover), p(shrub cover) | 4.35 (1.14) | - | 0.84 (1.56) | - | -0.43 (0.58) | - |
| psi(shrub cover), p(canopy cover) | 4.11 (0.86) | - | - | - | -0.54 (0.55) | - |
| psi(stand basal area), p(time from sunrise) | 3.98 (0.78) | - | - | 0.08 (0.82) | - | - |
| psi(canopy cover + shrub cover), p(.) | 4.14 (0.86) | -0.39 (0.97) | - | - | -0.71 (0.67) | - |
| psi(bamboo cover), p(canopy cover) | 4.23 (1.05) | - | 0.96 (1.47) | - | - | - |
| psi(stand basal area + shrub cover), p(.) | 4.14 (0.88) | - | - | -0.19 (0.74) | -0.61 (0.6) | - |
| psi(canopy cover + shrub cover), p(shrub cover) | 4.15 (0.86) | -0.44 (0.98) | - | - | -0.7 (0.69) | - |
| psi(distance to PA), p(canopy cover) | 4.08 (0.87) | - | - | - | - | -0.52 (0.73) |
| psi(canopy cover + bamboo cover), p(.) | 4.24 (1.05) | -0.23 (0.87) | 1.08 (1.51) | - | - | - |
| psi(canopy cover + bamboo cover), p(shrub cover) | 4.27 (1.05) | -0.35 (0.99) | 1.04 (1.47) | - | - | - |
| psi(bamboo cover + shrub cover), p(time from sunrise + shrub cover) | 4.41 (1.23) | - | 0.87 (1.64) | - | -0.45 (0.6) | - |
| psi(bamboo cover + shrub cover), p(time from sunrise) | 4.52 (1.42) | - | 0.96 (1.8) | - | -0.5 (0.6) | - |
| psi(canopy cover), p(canopy cover) | 3.95 (0.76) | 0.08 (0.78) | - | - | - | - |
| psi(stand basal area), p(canopy cover) | 3.95 (0.76) | - | - | 0.05 (0.78) | - | - |
| psi(canopy cover + shrub cover), p(time from sunrise + shrub cover) | 4.18 (0.89) | -0.44 (1.02) | - | - | -0.7 (0.7) | - |
| psi(canopy cover + bamboo cover), p(time from sunrise + shrub cover) | 4.33 (1.11) | -0.43 (1.21) | 1.03 (1.47) | - | - | - |
| psi(stand basal area + shrub cover), p(time from sunrise + shrub cover) | 4.17 (0.9) | - | - | -0.21 (0.75) | -0.59 (0.63) | - |
| psi(canopy cover), p(time from sunrise) | 7.39 (4.45) | 2.82 (2.84) | - | - | - | - |
| psi(stand basal area + shrub cover), p(time from sunrise) | 4.2 (0.94) | - | - | -0.18 (0.77) | -0.63 (0.62) | - |
| psi(canopy cover + bamboo cover + shrub cover), p(.) | 4.5 (1.21) | -0.8 (1.07) | 1.23 (1.67) | - | -0.74 (0.67) | - |
| psi(canopy cover + bamboo cover), p(time from sunrise) | 4.27 (1.08) | -0.21 (0.9) | 1.08 (1.55) | - | - | - |
| psi(canopy cover + bamboo cover + shrub cover), p(shrub cover) | 4.5 (1.19) | -0.86 (1.1) | 1.21 (1.63) | - | -0.73 (0.68) | - |
| psi(bamboo cover + shrub cover), p(canopy cover) | 4.37 (1.17) | - | 0.86 (1.59) | - | -0.45 (0.57) | - |
| psi(canopy cover + shrub cover), p(canopy cover) | 4.13 (0.85) | -0.4 (0.96) | - | - | -0.7 (0.67) | - |
| psi(stand basal area + shrub cover), p(canopy cover) | 4.12 (0.86) | - | - | -0.2 (0.73) | -0.6 (0.6) | - |
| psi(canopy cover + shrub cover), p(time from sunrise) | 7.7 (4.64) | 2.28 (2.93) | - | - | -0.79 (0.75) | - |
| psi(canopy cover + bamboo cover), p(canopy cover) | 4.24 (1.04) | -0.26 (0.87) | 1.07 (1.49) | - | - | - |
| psi(canopy cover + bamboo cover + shrub cover), p(time from sunrise + shrub cover) | 4.52 (1.21) | -0.86 (1.14) | 1.21 (1.65) | - | -0.73 (0.69) | - |
| psi(canopy cover + bamboo cover + shrub cover), p(time from sunrise) | 4.53 (1.25) | -0.78 (1.1) | 1.25 (1.72) | - | -0.74 (0.67) | - |
| psi(stand basal area + shrub cover), p(shrub cover) | 5.5 (3.28) | - | - | -0.55 (0.96) | 1.37 (3.23) | - |
| psi(canopy cover + bamboo cover + shrub cover + distance to PA), p(.) | 4.66 (1.36) | -0.88 (1.1) | 1.41 (1.83) | - | -0.61 (0.73) | -0.46 (0.79) |
| psi(canopy cover + bamboo cover + shrub cover), p(canopy cover) | 4.49 (1.19) | -0.82 (1.07) | 1.22 (1.65) | - | -0.74 (0.67) | - |
| psi(canopy cover + bamboo cover + shrub cover + distance to PA), p(shrub cover) | 4.62 (1.3) | -0.92 (1.1) | 1.37 (1.77) | - | -0.62 (0.74) | -0.43 (0.78) |
| psi(canopy cover + bamboo cover + shrub cover + distance to PA), p(time from sunrise + shrub cover) | 4.66 (1.36) | -0.91 (1.13) | 1.38 (1.83) | - | -0.62 (0.74) | -0.44 (0.81) |
| psi(canopy cover + bamboo cover + shrub cover + distance to PA), p(time from sunrise) | 4.74 (1.52) | -0.84 (1.14) | 1.47 (1.96) | - | -0.61 (0.75) | -0.5 (0.84) |
| psi(canopy cover + bamboo cover + shrub cover + distance to PA), p(canopy cover) | 4.64 (1.33) | -0.9 (1.09) | 1.4 (1.8) | - | -0.62 (0.73) | -0.45 (0.78) |

Large high-canopy gleaning insectivores

| **Model** | **Intercept** | **Canopy cover** | **Bamboo cover** | **Stand basal area** | **Shrub cover** | **Distance to PA** |
| --- | --- | --- | --- | --- | --- | --- |
| **psi(canopy cover), p(time from sunrise)*** | **2.5 (0.6)** | **-0.77 (0.53)** | **-** | **-** | **-** | **-** |
| psi(canopy cover + bamboo cover), p(time from sunrise) | 2.84 (0.86) | -1.34 (0.82) | 0.73 (0.65) | - | - | - |
| **psi(distance to PA), p(time from sunrise)*** | **2.77 (1.24)** | **-** | **-** | **-** | **-** | **1.12 (1.46)** |
| **psi(.), p(time from sunrise)** | **2.34 (0.5)** | **-** | **-** | **-** | **-** | **-** |
| psi(canopy cover), p(time from sunrise + shrub cover) | 2.48 (0.59) | -0.79 (0.52) | - | - | - | - |
| psi(canopy cover + bamboo cover), p(time from sunrise + shrub cover) | 2.83 (0.86) | -1.35 (0.82) | 0.72 (0.64) | - | - | - |
| psi(canopy cover + bamboo cover + shrub cover + distance to PA), p(time from sunrise) | 3.82 (1.96) | -1.57 (1.08) | 1.01 (0.73) | - | -0.29 (0.63) | 1.39 (1.76) |
| psi(canopy cover), p(time from sunrise + canopy cover) | 2.5 (0.6) | -0.75 (0.53) | - | - | - | - |
| psi(canopy cover + shrub cover), p(time from sunrise) | 2.5 (0.6) | -0.82 (0.63) | - | - | -0.08 (0.52) | - |
| psi(canopy cover + bamboo cover), p(time from sunrise + canopy cover) | 2.85 (0.87) | -1.32 (0.83) | 0.73 (0.67) | - | - | - |
| psi(canopy cover + bamboo cover + shrub cover), p(time from sunrise) | 2.85 (0.87) | -1.42 (0.93) | 0.73 (0.65) | - | -0.12 (0.56) | - |
| psi(distance to PA), p(time from sunrise + shrub cover) | 2.81 (1.48) | - | - | - | - | 1.18 (1.74) |
| psi(shrub cover), p(time from sunrise) | 2.37 (0.51) | - | - | - | 0.36 (0.46) | - |
| psi(stand basal area), p(time from sunrise) | 2.36 (0.51) | - | - | -0.28 (0.37) | - | - |
| psi(.), p(time from sunrise + shrub cover) | 2.31 (0.48) | - | - | - | - | - |
| psi(.), p(time from sunrise + canopy cover) | 2.41 (0.55) | - | - | - | - | - |
| psi(distance to PA), p(time from sunrise + canopy cover) | 2.78 (1.21) | - | - | - | - | 1.07 (1.44) |
| psi(bamboo cover), p(time from sunrise) | 2.35 (0.51) | - | 0.06 (0.47) | - | - | - |
| psi(canopy cover + bamboo cover + shrub cover + distance to PA), p(time from sunrise + shrub cover) | 3.87 (1.96) | -1.6 (1.07) | 1.02 (0.71) | - | -0.29 (0.63) | 1.42 (1.79) |
| psi(canopy cover + shrub cover), p(time from sunrise + shrub cover) | 2.49 (0.59) | -0.83 (0.62) | - | - | -0.07 (0.52) | - |
| psi(canopy cover + bamboo cover + shrub cover), p(time from sunrise + shrub cover) | 2.84 (0.86) | -1.44 (0.93) | 0.73 (0.64) | - | -0.11 (0.56) | - |
| psi(shrub cover), p(time from sunrise + shrub cover) | 2.35 (0.5) | - | - | - | 0.38 (0.46) | - |
| psi(canopy cover + bamboo cover + shrub cover + distance to PA), p(time from sunrise + canopy cover) | 3.85 (2.08) | -1.56 (1.09) | 1.02 (0.76) | - | -0.28 (0.64) | 1.41 (1.87) |
| psi(shrub cover), p(time from sunrise + canopy cover) | 2.42 (0.54) | - | - | - | 0.34 (0.47) | - |
| psi(stand basal area), p(time from sunrise + shrub cover) | 2.33 (0.5) | - | - | -0.29 (0.36) | - | - |
| psi(stand basal + shrub cover), p(time from sunrise) | 2.39 (0.53) | - | - | -0.21 (0.4) | 0.31 (0.48) | - |
| psi(bamboo cover + shrub cover), p(time from sunrise) | 2.43 (0.57) | - | 0.25 (0.54) | - | 0.45 (0.51) | - |
| psi(canopy cover + shrub cover), p(time from sunrise + canopy cover) | 2.5 (0.6) | -0.8 (0.64) | - | - | -0.08 (0.53) | - |
| psi(stand basal area), p(time from sunrise + canopy cover) | 2.4 (0.54) | - | - | -0.25 (0.39) | - | - |
| psi(canopy cover + bamboo cover + shrub cover), p(time from sunrise + canopy cover) | 2.86 (0.88) | -1.4 (0.95) | 0.74 (0.67) | - | -0.11 (0.56) | - |
| psi(bamboo cover), p(time from sunrise + canopy cover) | 2.42 (0.56) | - | 0.09 (0.51) | - | - | - |
| psi(bamboo cover), p(time from sunrise + shrub cover) | 2.32 (0.5) | - | 0.06 (0.47) | - | - | - |
| psi(stand basal area + shrub cover), p(time from sunrise + shrub cover) | 2.37 (0.52) | - | - | -0.22 (0.39) | 0.32 (0.47) | - |
| psi(bamboo cover + shrub cover), p(time from sunrise + shrub cover) | 2.41 (0.56) | - | 0.24 (0.53) | - | 0.47 (0.51) | - |
| psi(bamboo cover + shrub cover), p(time from sunrise + canopy cover) | 2.49 (0.61) | - | 0.28 (0.59) | - | 0.45 (0.53) | - |
| psi(stand basal area + shrub cover), p(time from sunrise + canopy cover) | 2.42 (0.55) | - | - | -0.18 (0.43) | 0.3 (0.48) | - |

Large understory gleaning insectivores

| **Model** | **Intercept** | **Canopy cover** | **Bamboo cover** | **Stand basal area** | **Shrub cover** | **Distance to PA** |
| --- | --- | --- | --- | --- | --- | --- |
| **psi(.), p(time from sunrise)*** | **1.63 (0.58)** | **-** | **-** | **-** | **-** | **-** |
| psi(canopy cover), p(time from sunrise) | 1.72 (0.7) | 0.46 (0.55) | - | - | - | - |
| psi(bamboo cover), p(time from sunrise) | 1.71 (0.69) | - | 0.49 (0.79) | - | - | - |
| psi(canopy cover + shrub cover), p(time from sunrise) | 2.39 (1.42) | 1.32 (1.11) | - | - | 1.14 (1.07) | - |
| psi(stand basal area), p(time from sunrise) | 1.63 (0.58) | - | - | -0.21 (0.35) | - | - |
| psi(.), p(time from sunrise + canopy cover) | 1.65 (0.59) | - | - | - | - | - |
| psi(distance to PA), p(time from sunrise) | 1.63 (0.58) | - | - | - | - | 0.13 (0.41) |
| psi(.), p(time from sunrise + shrub cover) | 1.63 (0.58) | - | - | - | - | - |
| psi(shrub cover), p(time from sunrise) | 1.62 (0.58) | - | - | - | -0.02 (0.45) | - |
| psi(canopy cover + bamboo cover), p(time from sunrise) | 1.82 (0.88) | 0.36 (0.57) | 0.42 (0.95) | - | - | - |
| psi(canopy cover), p(time from sunrise + canopy cover) | 1.82 (0.86) | 0.64 (0.79) | - | - | - | - |
| psi(canopy cover), p(time from sunrise + shrub cover) | 1.76 (0.75) | 0.52 (0.61) | - | - | - | - |
| psi(bamboo cover), p(time from sunrise shrub cover) | 1.73 (0.74) | - | 0.56 (0.9) | - | - | - |
| psi(canopy cover + shrub cover), p(time frm sunrise + canopy cover) | 2.55 (1.55) | 1.54 (1.21) | - | - | 1.09 (1.08) | - |
| psi(bamboo cover + shrub cover), p(time from sunrise) | 1.7 (0.68) | - | 0.49 (0.77) | - | 0.04 (0.45) | - |
| psi(bamboo cover), p(time from sunrise + canopy cover) | 1.7 (0.68) | - | 0.47 (0.83) | - | - | - |
| psi(stand basal area), p(time from sunrise + canopy cover) | 1.68 (0.6) | - | - | -0.28 (0.37) | - | - |
| psi(canopy cover + bamboo cover + shrub cover), p(time from sunrise) | 2.58 (1.75) | 1.29 (1.12) | 0.49 (1.38) | - | 1.16 (1.11) | - |
| psi(canopy cover + shrub cover), p(time from sunrise + shrub cover) | 2.29 (1.19) | 1.24 (0.96) | - | - | 1.21 (1) | - |
| psi(stand basal area + shrub cover), p(time from sunrise) | 1.63 (0.58) | - | - | -0.26 (0.38) | -0.17 (0.47) | - |
| psi(stand basal area), p(time from sunrise + shrub cover) | 1.63 (0.58) | - | - | -0.21 (0.36) | - | - |
| psi(distance to PA), p(time from sunrise + canopy cover) | 1.66 (0.59) | - | - | - | - | 0.16 (0.43) |
| psi(shrub cover), p(time from sunrise + canopy cover) | 1.65 (0.6) | - | - | - | 0.04 (0.53) | - |
| psi(distance to PA), p(time from sunrise + shrub cover) | 1.62 (0.57) | - | - | - | - | 0.13 (0.42) |
| psi(shrub cover), p(time from sunrise + shrub cover) | 1.62 (0.58) | - | - | - | -0.07 (0.55) | - |
| psi(canopy cover + bamboo cover + shrub cover + distance to PA), p(time from sunrise) | 5 (3.55) | 1.9 (1.4) | 2.91 (2.92) | - | 2.18 (1.75) | -1.17 (1.37) |
| psi(canopy cover + bamboo cover), p(time from sunrise + canopy cover) | 1.99 (1.07) | 0.55 (0.71) | 0.57 (1.16) | - | - | - |
| psi(canopy cover + bamboo cover), p(time from sunrise + shrub cover) | 1.92 (1.03) | 0.43 (0.61) | 0.54 (1.14) | - | - | - |
| psi(canopy cover + bamboo cover + shrub cover), p(time from sunrise + canopy cover) | 2.67 (1.55) | 1.42 (1.04) | 0.6 (1.33) | - | 1.08 (1.09) | - |
| psi(bamboo cover + shrub cover), p(time from sunrise + shrub cover) | 1.76 (0.82) | - | 0.58 (0.99) | - | -0.05 (0.51) | - |
| psi(bamboo cover + shrub cover), p(time from sunrise + canopy cover) | 1.7 (0.66) | - | 0.46 (0.79) | - | 0.06 (0.49) | - |
| psi(stand basal area + shrub cover), p(time from sunrise + canopy cover) | 1.67 (0.59) | - | - | -0.31 (0.39) | -0.12 (0.51) | - |
| psi(canopy cover + bamboo cover + shrub cover), p(time from sunrise + shrub cover) | 2.47 (1.67) | 1.23 (1.08) | 0.4 (1.3) | - | 1.21 (1.06) | - |
| psi(stand basal area + shrub cover), p(time from sunrise + shrub cover) | 1.64 (0.59) | - | - | -0.28 (0.39) | -0.24 (0.54) | - |
| psi(canopy cover + bamboo cover + shrub cover + distance to PA), p(time from sunrise + canopy cover) | 4.5 (2.97) | 1.82 (1.21) | 2.58 (2.62) | - | 1.93 (1.5) | -1 (1.18) |
| psi(canopy cover + bamboo cover + shrub cover + distance to PA), p(time from sunrise + shrub cover) | 5.05 (3.65) | 1.93 (1.43) | 2.92 (2.96) | - | 2.26 (1.83) | -1.19 (1.41) |

Large high-canopy sallying insectivores

| **Model** | **Intercept** | **Canopy cover** | **Bamboo cover** | **Stand basal area** | **Shrub cover** | **Distance to PA** |
| --- | --- | --- | --- | --- | --- | --- |
| **psi(shrub cover), p(time from sunrise + canopy cover)*** | **2.46 (0.51)** | **-** | **-** | **-** | **-1.09 (0.37)** | **-** |
| **psi(shrub cover), p(canopy cover)** | **2.45 (0.52)** | **-** | **-** | **-** | **-1.07 (0.37)** | **-** |
| psi(stand basal area + shrub cover), p(time from sunrise + canopy cover) | 2.56 (0.59) | - | - | -0.31 (0.39) | -1.23 (0.43) | - |
| **psi(shrub cover), p(time from sunrise + canopy cover + shrub cover)** | **2.46 (0.51)** | **-** | **-** | **-** | **-1.11 (0.37)** | **-** |
| psi(bamboo cover + shrub cover), p(time from sunrise + canopy cover) | 2.45 (0.51) | - | -0.14 (0.6) | - | -1.12 (0.4) | - |
| psi(canopy cover + shrub cover), p(time from sunrise + canopy cover) | 2.46 (0.52) | -0.05 (0.5) | - | - | -1.12 (0.45) | - |
| psi(stand basal area + shrub cover), p(canopy cover) | 2.56 (0.61) | - | - | -0.31 (0.4) | -1.21 (0.44) | - |
| psi(bamboo cover + shrub cover), p(canopy cover) | 2.45 (0.53) | - | -0.32 (0.53) | - | -1.15 (0.41) | - |
| psi(stand basal area + shrub cover), p(time from sunrise + canopy cover + shrub cover) | 2.56 (0.58) | - | - | -0.32 (0.38) | -1.25 (0.43) | - |
| psi(canopy cover + shrub cover), p(canopy cover) | 2.45 (0.53) | -0.11 (0.5) | - | - | -1.13 (0.45) | - |
| psi(bamboo cover + shrub cover), p(time from sunrise + canopy cover + shrub cover) | 2.45 (0.51) | - | -0.11 (0.61) | - | -1.14 (0.39) | - |
| psi(canopy cover + shrub cover), p(time from sunrise + canopy cover + shrub cover) | 2.46 (0.51) | -0.05 (0.49) | - | - | -1.14 (0.44) | - |
| psi(canopy cover + bamboo cover + shrub cover), p(time from sunrise + canopy cover) | 2.45 (0.51) | 0 (0.55) | -0.13 (0.68) | - | -1.12 (0.45) | - |
| psi(canopy cover + bamboo cover + shrub cover), p(canopy cover) | 2.45 (0.53) | 0.02 (0.56) | -0.33 (0.6) | - | -1.15 (0.46) | - |
| psi(canopy cover + bamboo cover + shrub cover), p(time from sunrise + canopy cover + shrub cover) | 2.45 (0.51) | -0.02 (0.55) | -0.1 (0.69) | - | -1.14 (0.44) | - |
| psi(canopy cover + bamboo cover + shrub cover + distance to PA), p(time from sunrise + canopy cover) | 2.5 (0.58) | 0.02 (0.57) | -0.15 (0.7) | - | -1.25 (0.59) | 0.24 (0.6) |
| psi(canopy cover + bamboo cover + shrub cover + distance to PA), p(canopy cover) | 2.52 (0.61) | 0.06 (0.58) | -0.36 (0.62) | - | -1.27 (0.61) | 0.24 (0.61) |
| psi(canopy cover + bamboo cover + shrub cover + distance to PA), p(time from sunrise + canopy cover + shrub cover) | 2.52 (0.6) | 0.01 (0.58) | -0.11 (0.71) | - | -1.29 (0.61) | 0.27 (0.61) |
| psi(.), p(time from sunrise + canopy cover) | 2.22 (0.44) | - | - | - | - | - |
| psi(canopy cover), p(time from sunrise + canopy cover) | 2.21 (0.43) | 0.56 (0.4) | - | - | - | - |
| psi(distance to PA), p(time from sunrise + canopy cover) | 2.25 (0.46) | - | - | - | - | -0.46 (0.39) |
| psi(.), p(canopy cover) | 2.2 (0.44) | - | - | - | - | - |
| psi(canopy cover), p(canopy cover) | 2.17 (0.41) | 0.52 (0.39) | - | - | - | - |
| psi(.), p(time from sunrise + canopy cover + shrub cover) | 2.34 (0.51) | - | - | - | - | - |
| psi(distance to PA), p(canopy cover) | 2.23 (0.45) | - | - | - | - | -0.46 (0.38) |
| psi(bamboo cover), p(time from sunrise + canopy cover) | 2.22 (0.44) | - | 0.19 (0.52) | - | - | - |
| psi(stand basal area), p(time from sunrise + canopy cover) | 2.18 (0.44) | - | - | 0.13 (0.51) | - | - |
| psi(canopy cover + bamboo cover), p(time from sunrise + canopy cover) | 2.2 (0.42) | 0.59 (0.44) | -0.1 (0.57) | - | - | - |
| psi(canopy cover), p(time from sunrise + canopy cover + shrub cover) | 2.24 (0.46) | 0.55 (0.42) | - | - | - | - |
| psi(distance to PA), p(time from sunrise + canopy cover + shrub cover) | 2.33 (0.53) | - | - | - | - | -0.45 (0.42) |
| psi(stand basal area), p(canopy cover) | 2.16 (0.43) | - | - | 0.15 (0.48) | - | - |
| psi(bamboo cover), p(canopy cover) | 2.19 (0.44) | - | 0.05 (0.44) | - | - | - |
| psi(canopy cover + bamboo cover), p(canopy cover) | 2.17 (0.41) | 0.61 (0.44) | -0.24 (0.5) | - | - | - |
| psi(bamboo cover), p(time from sunrise + canopy cover + shrub cover) | 2.32 (0.51) | - | 0.1 (0.55) | - | - | - |
| psi(stand basal area), p(time from sunrise + canopy cover + shrub cover) | 2.35 (0.61) | - | - | -0.02 (0.56) | - | - |
| psi(canopy cover + bamboo cover), p(time from sunrise + canopy cover + shrub cover) | 2.23 (0.45) | 0.58 (0.45) | -0.12 (0.58) | - | - | - |

Small mid-canopy gleaning insectivores

| **Model** | **Intercept** | **Canopy cover** | **Bamboo cover** | **Stand basal area** | **Shrub cover** | **Distance to PA** |
| --- | --- | --- | --- | --- | --- | --- |
| **psi(distance to PA), p(time from sunrise + shrub cover)*** | **5.78 (5.2)** | **-** | **-** | **-** | **-** | **3.5 (5.59)** |
| **psi(.), p(time from sunrise)** | **3.64 (0.8)** | **-** | **-** | **-** | **-** | **-** |
| **psi(.), p(time from sunrise + shrub cover)** | **3.54 (0.74)** | **-** | **-** | **-** | **-** | **-** |
| psi(shrub cover), p(time from sunrise) | 3.75 (0.85) | - | - | - | 0.79 (0.89) | - |
| psi(canopy cover), p(time from sunrise) | 3.71 (0.92) | -0.32 (0.92) | - | - | - | - |
| psi(stand basal area), p(time from sunrise) | 3.66 (0.82) | - | - | -0.23 (0.63) | - | - |
| psi(bamboo cover), p(time from sunrise) | 3.64 (0.79) | - | 0.26 (0.81) | - | - | - |
| psi(shrub cover), p(time from sunrise + shrub cover) | 3.69 (0.81) | - | - | - | 0.81 (0.87) | - |
| psi(bamboo cover + shrub cover), p(time from sunrise) | 3.83 (0.9) | - | 0.51 (0.8) | - | 0.96 (0.92) | - |
| psi(canopy cover + bamboo cover + shrub cover + distance to PA), p(time from sunrise) | 6.67 (4.35) | 0.7 (0.88) | 0.49 (0.74) | - | 0.66 (1.17) | 4.64 (5.23) |
| psi(canopy cover + shrub cover), p(time from sunrise) | 3.86 (1) | 0.51 (0.91) | - | - | 1.21 (1.23) | - |
| psi(bamboo cover), p(time from sunrise + shrub cover) | 3.55 (0.74) | - | 0.29 (0.78) | - | - | - |
| psi(stand basal area), p(time from sunrise + shrub cover) | 3.57 (0.76) | - | - | -0.23 (0.59) | - | - |
| psi(canopy cover), p(time from sunrise + shrub cover) | 3.6 (0.82) | -0.26 (0.83) | - | - | - | - |
| psi(stand basal area + shrub cover), p(time from sunrise) | 3.75 (0.86) | - | - | -0.04 (0.74) | 0.78 (0.93) | - |
| psi(canopy cover + bamboo cover + shrub cover + distance to PA), p(time from sunrise + shrub cover) | 6.48 (3.77) | 0.72 (0.86) | 0.5 (0.73) | - | 0.7 (1.13) | 4.44 (4.56) |
| psi(bamboo cover + shrub cover), p(time from sunrise + shrub cover) | 3.78 (0.87) | - | 0.53 (0.78) | - | 0.98 (0.9) | - |
| psi(canopy cover + bamboo cover), p(time from sunrise) | 3.8 (1.02) | -0.64 (1.15) | 0.51 (0.86) | - | - | - |
| psi(canopy cover + shrub cover), p(time from sunrise + shrub cover) | 3.81 (0.95) | 0.51 (0.84) | - | - | 1.23 (1.17) | - |
| psi(stand basal area + shrub cover), p(time from sunrise + shrub cover) | 3.69 (0.81) | - | - | -0.04 (0.69) | 0.79 (0.89) | - |
| psi(canopy cover + bamboo cover), p(time from sunrise + shrub cover) | 3.67 (0.89) | -0.55 (1.02) | 0.51 (0.84) | - | - | - |
| psi(canopy cover + bamboo cover + shrub cover), p(time from sunrise) | 3.89 (1.02) | 0.31 (0.98) | 0.45 (0.85) | - | 1.19 (1.23) | - |
| psi(canopy cover + bamboo cover + shrub cover), p(time from sunrise + shrub cover) | 3.86 (0.98) | 0.32 (0.92) | 0.46 (0.83) | - | 1.22 (1.18) | - |

Small understory gleaning insectivores

| **Model** | **Intercept** | **Canopy cover** | **Bamboo cover** | **Stand basal area** | **Shrub cover** | **Distance to PA** |
| --- | --- | --- | --- | --- | --- | --- |
| **psi(canopy cover), p(time from sunrise + canopy cover + shrub cover)*** | **2.99 (0.83)** | **-1.51 (0.71)** | **-** | **-** | **-** | **-** |
| **psi(stand basal area), p(time from sunrise + canopy cover + shrub cover)*** | **3.42 (1.3)** | **-** | **-** | **-1.09 (0.6)** | **-** | **-** |
| **psi(.), p(time from sunrise + canopy cover + shrub cover)** | **3.84 (1.46)** | **-** | **-** | **-** | **-** | **-** |
| psi(canopy cover + bamboo cover), p(time from sunrise + canopy cover + shrub cover) | 3.08 (0.9) | -1.73 (0.86) | 0.28 (0.55) | - | - | - |
| psi(canopy cover + shrub cover), p(time from sunrise + canopy cover + shrub cover) | 2.99 (0.84) | -1.53 (0.86) | - | - | -0.03 (0.73) | - |
| psi(stand basal area + shrub cover), p(time from sunrise + canopy cover + shrub cover) | 3.27 (1.13) | - | - | -0.99 (0.59) | 0.53 (0.8) | - |
| psi(shrub cover), p(time from sunrise + canopy cover + shrub cover) | 3.38 (1.11) | - | - | - | 0.91 (1.06) | - |
| psi(bamboo cover), psi(time from sunrise + canopy cover + shrub cover) | 3.67 (1.44) | - | -0.41 (1.03) | - | - | - |
| psi(distance to PA), p(time from sunrise + canopy cover + shrub cover) | 3.98 (2.1) | - | - | - | - | -0.11 (1.8) |
| psi(canopy cover + bamboo cover + shrub cover), p(time from sunrise + canopy cover + shrub cover) | 3.08 (0.9) | -1.7 (0.96) | 0.28 (0.56) | - | 0.05 (0.75) | - |
| psi(bamboo cover + shrub cover), p(time from sunrise + canopy cover + shrub cover) | 3.49 (1.31) | - | 0.2 (1.1) | - | 1.04 (1.24) | - |
| psi(canopy cover + bamboo cover + shrub cover + distance to PA), p(time from sunrise + canopy cover + shrub cover) | 3.12 (0.99) | -1.71 (0.99) | 0.29 (0.54) | - | -0.07 (0.79) | 0.3 (0.77) |

Small mid-canopy sallying insectivores

| **Model** | **Intercept** | **Canopy cover** | **Bamboo cover** | **Stand basal area** | **Shrub cover** | **Distance to PA** |
| --- | --- | --- | --- | --- | --- | --- |
| **psi(bamboo cover + shrub cover), p(canopy cover + shrub cover)*** | **4.24 (2.71)** | **-** | **3.41 (3.33)** | **-** | **-0.63 (0.37)** | **-** |
| **psi(bamboo cover), p(canopy cover + shrub cover)** | **3.4 (1.33)** | **-** | **2.63 (1.73)** | **-** | **-** | **-** |
| psi(canopy cover + bamboo cover), p(canopy cover + shrub cover) | 3.12 (1.06) | 0.47 (0.49) | 2.14 (1.45) | - | - | - |
| psi(shrub cover), p(canopy cover + shrub cover) | 2.46 (0.6) | - | - | - | -0.69 (0.36) | - |
| psi(canopy cover), p(canopy cover + shrub cover) | 2.18 (0.47) | 0.77 (0.47) | - | - | - | - |
| psi(stand basal area + shrub cover), p(canopy cover + shrub cover) | 2.45 (0.59) | - | - | 0.24 (0.53) | -0.62 (0.38) | - |
| psi(canopy cover + shrub cover), p(canopy cover + shrub cover) | 2.38 (0.59) | 0.31 (0.67) | - | - | -0.55 (0.47) | - |
| psi(.), p(canopy cover + shrub cover) | 2.31 (0.51) | - | - | - | - | - |
| psi(stand basal area), p(canopy cover + shrub cover) | 2.3 (0.51) | - | - | 0.55 (0.54) | - | - |
| psi(distance to PA), p(canopy cover + shrub cover) | 2.36 (0.57) | - | - | - | - | 0.32 (0.52) |

Large woodpeckers

| **Model** | **Intercept** | **Canopy cover** | **Bamboo cover** | **Stand basal area** | **Shrub cover** | **Distance to PA** |
| --- | --- | --- | --- | --- | --- | --- |
| **psi(shrub cover), p(time from sunrise + canopy cover)*** | **5.62 (3.64)** | **-** | **-** | **-** | **3.53 (3.03)** | **-** |
| **psi(.), p(time from sunrise + canopy cover)** | **2.27 (0.69)** | **-** | **-** | **-** | **-** | **-** |
| psi(stand basal area + shrub cover), p(time from sunrise + canopy cover) | 8.32 (5.24) | - | - | -1.18 (1.44) | 4.88 (3.26) | - |
| psi(shrub cover), p(time from sunrise + canopy cover + shrub cover) | 5.55 (3.53) | - | - | - | 3.57 (2.93) | - |
| psi(distance to PA), p(time from sunrise + canopy cover) | 2.17 (0.66) | - | - | - | - | -0.54 (0.49) |
| psi(canopy cover + shrub cover), p(time from sunrise + canopy cover) | 3.91 (2.92) | 0.92 (1.14) | - | - | 2.89 (2.38) | - |
| psi(bamboo cover + shrub cover), p(time from sunrise + canopy cover) | 5.9 (3.81) | - | -0.18 (0.47) | - | 3.64 (3.08) | - |
| psi(canopy cover + shrub cover), p(time from sunrise + canopy cover + shrub cover) | 3.21 (1.68) | 1.28 (0.78) | - | - | 2.71 (1.61) | - |
| psi(stand basal area), p(time from sunrise + canopy cover) | 2.69 (1.25) | - | - | -0.36 (0.71) | - | - |
| psi(.), p(time from sunrise + canopy cover + shrub cover) | 2.24 (0.68) | - | - | - | - | - |
| psi(stand basal area + shrub cover), p(time from sunrise + canopy cover + shrub cover) | 7.32 (4.25) | - | - | -0.8 (1.31) | 4.51 (2.88) | - |
| psi(canopy cover), p(time from sunrise + canopy cover) | 2.63 (1.55) | -0.35 (1.2) | - | - | - | - |
| psi(bamboo cover), p(time from sunrise + canopy cover) | 2.3 (0.76) | - | -0.05 (0.49) | - | - | - |
| psi(canopy cover + bamboo cover + shrub cover), p(time from sunrise + canopy cover + shrub cover) | 3.8 (2.04) | 1.85 (1.09) | -0.56 (0.56) | - | 3.35 (2.02) | - |
| psi(canopy cover + bamboo cover + shrub cover), p(time from sunrise + canopy cover) | 4.21 (2.42) | 1.66 (1.31) | -0.54 (0.6) | - | 3.43 (2.38) | - |
| psi(bamboo cover + shrub cover), p(time from sunrise + canopy cover + shrub cover) | 5.71 (3.6) | - | -0.16 (0.45) | - | 3.6 (2.93) | - |
| psi(shrub cover), p(canopy cover + shrub cover) | 6.29 (4.4) | - | - | - | 4.17 (3.49) | - |
| psi(distance to PA), p(time from sunrise + canopy cover + shrub cover) | 2.15 (0.65) | - | - | - | - | -0.51 (0.5) |
| psi(canopy cover + shrub cover), p(canopy cover + shrub cover) | 3.41 (1.82) | 1.48 (0.8) | - | - | 3 (1.74) | - |
| psi(canopy cover + bamboo cover + shrub cover), p(canopy cover + shrub cover) | 5.13 (5.44) | 2.61 (2.68) | -0.82 (1.11) | - | 4.64 (5.1) | - |
| psi(stand basal area), p(time from sunrise + canopy cover + shrub cover) | 2.58 (1.26) | - | - | -0.3 (0.75) | - | - |
| psi(canopy cover), p(time from sunrise + canopy cover + shrub cover) | 2.53 (1.45) | -0.28 (1.12) | - | - | - | - |
| psi(bamboo cover), p(time from sunrise + canopy cover + shrub cover) | 2.28 (0.76) | - | -0.07 (0.48) | - | - | - |
| psi(canopy cover + bamboo cover), p(time from sunrise + canopy cover) | 2.67 (1.71) | -0.4 (1.49) | 0.05 (0.58) | - | - | - |
| psi(canopy cover + bamboo cover + shrub cover + distance to PA), p(time from sunrise + canopy cover) | 4.11 (2.33) | 1.56 (1.38) | -0.61 (0.63) | - | 3.36 (2.41) | -0.3 (0.79) |
| psi(canopy cover + bamboo cover + shrub cover + distance to PA), p(time from sunrise + canopy cover + shrub cover) | 3.83 (2.11) | 1.82 (1.13) | -0.61 (0.59) | - | 3.4 (2.12) | -0.2 (0.68) |
| psi(.), p(canopy cover + shrub cover) | 2.28 (0.8) | - | - | - | - | - |
| psi(bamboo cover + shrub cover), p(canopy cover, shrub cover) | 6.73 (5.08) | - | -0.2 (0.49) | - | 4.38 (3.86) | - |
| psi(stand basal area + shrub cover), p(canopy cover + shrub cover) | 6.53 (4.1) | - | - | -0.21 (1.1) | 4.3 (3.19) | - |
| psi(canopy cover + bamboo cover + shrub cover + distance to PA), p(canopy cover + shrub cover) | 5.48 (9.38) | 2.76 (4.54) | -0.93 (2.05) | - | 5 (8.98) | -0.15 (1.3) |
| psi(distance to PA), p(canopy cover + shrub cover) | 2.22 (0.82) | - | - | - | - | -0.6 (0.56) |
| psi(canopy cover + bamboo cover), p(time from sunrise + canopy cover + shrub cover) | 2.53 (1.49) | -0.28 (1.3) | 0 (0.57) | - | - | - |
| psi(bamboo cover), p(canopy cover + shrub cover) | 2.3 (0.86) | - | -0.05 (0.51) | - | - | - |
| psi(canopy cover), p(canopy cover + shrub cover) | 2.33 (1.25) | -0.05 (0.94) | - | - | - | - |
| psi(stand basal area), p(canopy cover + shrub cover) | 2.28 (1.24) | - | - | -0.01 (1.04) | - | - |
| psi(canopy cover + bamboo cover), p(canopy cover + shrub cover) | 2.3 (1.21) | 0 (1.02) | -0.05 (0.57) | - | - | - |
