# Supplementary material for: “In a tree by the brook, there’s a songbird who sings”: Woodlands in an agricultural matrix maintain functionality of a wintering bird community
Source: PLoS One. 2018 Aug 2;13(8):e0201657. doi: 10.1371/journal.pone.0201657 (PMC6072076; doi:10.1371/journal.pone.0201657)
Supplement: S4 File — Standard errors are shown in parentheses. Model selection was conducted using AIC, while removing all uninformative models. Chosen models for inference are highlighted in bold. Covariate coefficients for detection probability are reported from models marked ‘*’. (DOCX) [file pone.0201657.s004.docx]

**S4 File**. **Covariate coefficients of detection probability for all the models of each guild.** Standard errors are shown in parentheses. Model selection was conducted using AIC, while removing all uninformative models. Chosen models for inference are highlighted in bold. Covariate coefficients for detection probability are reported from models marked ‘*’.

Nectarivores

| **Model** | **Intercept** | **Time from sunrise** | **Canopy cover** | **Shrub cover** |
| --- | --- | --- | --- | --- |
| **psi(shrub cover), p(.)*** | **0.16 (0.09)** | **-** | **-** | **-** |
| psi(canopy cover + shrub cover), p(.) | 0.16 (0.09) | - | - | - |
| psi(stand basal area + shrub cover), p(.) | 0.16 (0.09) | - | - | - |
| psi(shrub cover), p(canopy cover) | 0.16 (0.09) | - | 0.05 (0.1) | - |
| psi(bamboo cover + shrub cover), p(.) | 0.16 (0.09) | - | - | - |
| psi(shrub cover), p(shrub cover) | 0.16 (0.09) | - | - | -0.03 (0.09) |
| psi(shrub cover), p(time from sunrise) | 0.16 (0.09) | 0.02 (0.1) | - | - |
| psi(canopy cover), p(.) | 0.15 (0.1) | - | - | - |
| psi(canopy cover + shrub cover),p (canopy cover) | 0.17 (0.09) | - | 0.05 (0.1) | - |
| psi(canopy cover + shrub cover), p(shrub cover) | 0.17 (0.09) | - | - | -0.04 (0.09) |
| psi(canopy cover + bamboo cover + shrub cover), p(.) | 0.16 (0.09) | - | - | - |
| psi(canopy cover + shrub cover), p(time from sunrise) | 0.16 (0.09) | 0.01 (0.1) | - | - |
| psi(stand basal area + shrub cover), p(canopy cover) | 0.16 (0.09) | - | 0.05 (0.1) | - |
| psi(stand basal area + shrub cover), p(shrub cover) | 0.16 (0.09) | - | - | -0.03 (0.1) |
| psi(.), p(.) | 0.14 (0.1) | - | - | - |
| psi(stand basal area + shrub cover), p(time from sunrise) | 0.16 (0.09) | 0.01 (0.1) | - | - |
| psi(distance to PA), p(.) | 0.15 (0.1) | - | - | - |
| psi(bamboo cover + shrub cover), p(canopy cover) | 0.17 (0.09) | - | 0.05 (0.1) | - |
| psi(bamboo cover), p(.) | 0.15 (0.1) | - | - | - |
| psi(bamboo cover + shrub cover), p(shrub cover) | 0.16 (0.09) | - | - | -0.04 (0.09) |
| psi(bamboo cover + shrub cover), p(time from sunrise) | 0.16 (0.09) | 0.02 (0.1) | - | - |
| psi(canopy cover), p(canopy cover) | 0.16 (0.1) | - | 0.04 (0.1) | - |
| psi(stand basal area), p(.) | 0.14 (0.1) | - | - | - |
| psi(canopy cover), p(shrub cover) | 0.15 (0.1) | - | - | -0.03 (0.1) |
| psi(canopy cover + bamboo cover), p(.) | 0.15 (0.09) | - | - | - |
| psi(canopy cover + bamboo cover + shrub cover), p(canopy cover) | 0.17 (0.09) | - | 0.05 (0.1) | - |
| psi(canopy cover), p(time from sunrise) | 0.15 (0.1) | 0.02 (0.1) | - | - |
| psi(canopy cover + bamboo cover + shrub cover + distance to PA), p(.) | 0.16 (0.09) | - | - | - |
| psi(canopy cover + bamboo cover + shrub cover), p(shrub cover) | 0.17 (0.09) | - | - | -0.04 (0.09) |
| psi(canopy cover + bamboo cover + shrub cover), p(time from sunrise) | 0.16 (0.09) | 0.01 (0.1) | - | - |
| psi(.), p(time from sunrise) | 0.14 (0.1) | 0.04 (0.1) | - | - |
| psi(.), p(canopy cover) | 0.14 (0.1) | - | 0 (0.1) | - |
| psi(.), p(shrub cover) | 0.14 (0.1) | - | - | 0 (0.1) |
| psi(distance to PA), p(time from sunrise) | 0.15 (0.1) | 0.04 (0.1) | - | - |
| psi(distance to PA), p(canopy cover) | 0.16 (0.1) | - | 0.01 (0.1) | - |
| psi(distance to PA), p(shrub cover) | 0.16 (0.1) | - | - | -0.01 (0.1) |
| psi(bamboo cover), p(time from sunrise) | 0.15 (0.1) | 0.04 (0.1) | - | - |
| psi(bamboo cover), p(canopy cover) | 0.15 (0.1) | - | 0.01 (0.1) | - |
| psi(bamboo cover), p(shrub cover) | 0.15 (0.1) | - | - | 0 (0.1) |
| psi(canopy cover + bamboo cover + shrub cover + distance to PA), p(canopy cover) | 0.17 (0.09) | - | 0.05 (0.1) | - |
| psi(canopy cover + bamboo cover), p(canopy cover) | 0.16 (0.1) | - | 0.04 (0.1) | - |
| psi(stand basal area), p(time from sunrise) | 0.14 (0.1) | 0.03 (0.1) | - | - |
| psi(stand basal area), p(canopy cover) | 0.15 (0.1) | - | 0.01 (0.1) | - |
| psi(stand basal area), p(shrub cover) | 0.14 (0.1) | - | - | 0 (0.1) |
| psi(canopy cover + bamboo cover), p(shrub cover) | 0.15 (0.1) | - | - | -0.03 (0.1) |
| psi(canopy cover + bamboo cover + shrub cover + distance to PA), p(shrub cover) | 0.17 (0.09) | - | - | -0.04 (0.09) |
| psi(canopy cover + bamboo cover), p(time from sunrise) | 0.15 (0.1) | 0.01 (0.1) | - | - |
| psi(canopy cover + bamboo cover + shrub cover + distance to PA), p(time from sunrise) | 0.16 (0.09) | 0.01 (0.1) | - | - |

Granivores

| **Model** | **Intercept** | **Time from sunrise** | **Canopy cover** | **Shrub cover** |
| --- | --- | --- | --- | --- |
| **psi(canopy cover + bamboo cover), p(time from sunrise)** | **-0.88 (0.18)** | **-0.42 (0.17)** | **-** | **-** |
| **psi(bamboo cover + shrub cover), p(time from sunrise + canopy cover)** | **-1.03 (0.21)** | **-0.43 (0.17)** | **-0.25 (0.17)** | **-** |
| **psi(bamboo cover + shrub cover), p(time from sunrise)** | **-0.88 (0.18)** | **-0.43 (0.17)** | **-** | **-** |
| **psi(canopy cover + bamboo cover), p(time from sunrise + canopy cover)** | **-0.99 (0.21)** | **-0.43 (0.17)** | **-0.19 (0.17)** | **-** |
| **psi(canopy cover + bamboo cover + shrub cover), p(time from sunrise)** | **-0.87 (0.17)** | **-0.42 (0.17)** | **-** | **-** |
| **psi(canopy cover + bamboo cover + shrub cover), p(time from sunrise + canopy cover)*** | **-0.99 (0.21)** | **-0.43 (0.17)** | **-0.2 (0.17)** | **-** |
| **psi(canopy cover + bamboo cover), p(time from sunrise + shrub cover)** | **-0.92 (0.19)** | **-0.44 (0.18)** | **-** | **0.09 (0.14)** |
| **psi(bamboo cover), p(time from sunrise + canopy cover)** | **-1.11 (0.22)** | **-0.44 (0.17)** | **-0.31 (0.17)** | **-** |
| psi(bamboo cover + shrub cover), p(time from sunrise + shrub cover) | -0.91 (0.19) | -0.45 (0.18) | - | 0.06 (0.14) |
| psi(canopy cover + bamboo cover + shrub cover), p(time from sunrise + shrub cover) | -0.9 (0.19) | -0.43 (0.18) | - | 0.06 (0.14) |
| psi(canopy cover + bamboo cover + shrub cover + distance to PA), p(time from sunrise) | -0.87 (0.18) | -0.42 (0.17) | - | - |
| psi(canopy cover), p(time from sunrise) | -0.87 (0.18) | -0.42 (0.17) | - | - |
| psi(canopy cover + bamboo cover + shrub cover + distance to PA), p(time from sunrise + canopy cover) | -0.99 (0.21) | -0.43 (0.17) | -0.2 (0.17) | - |
| psi(bamboo cover), p(time from sunrise) | -0.91 (0.18) | -0.45 (0.17) | - | - |
| psi(canopy cover), p(time from sunrise + canopy cover) | -1.01 (0.21) | -0.43 (0.17) | -0.22 (0.17) | - |
| psi(canopy cover + shrub cover), p(time from sunrise) | -0.86 (0.17) | -0.42 (0.17) | - | - |
| psi(canopy cover + shrub cover), p(time from sunrise + canopy cover) | -1 (0.21) | -0.43 (0.17) | -0.23 (0.17) | - |
| psi(bamboo cover), p(time from sunrise + shrub cover) | -0.99 (0.2) | -0.47 (0.17) | - | 0.14 (0.14) |
| psi(canopy cover + bamboo cover + shrub cover + distance to PA), p(time from sunrise + shrub cover) | -0.9 (0.19) | -0.44 (0.18) | - | 0.06 (0.14) |
| psi(canopy cover), p(time from sunrise + shrub cover) | -0.92 (0.19) | -0.45 (0.18) | - | 0.09 (0.14) |
| psi(shrub cover), p(time from sunrise + canopy cover) | -1.13 (0.21) | -0.45 (0.17) | -0.36 (0.17) | - |
| psi(canopy cover + shrub cover), p(time from sunrise + shrub cover) | -0.9 (0.19) | -0.44 (0.18) | - | 0.07 (0.14) |
| psi(stand basal area + shrub cover), p(time from sunrise + canopy cover) | -1.22 (0.26) | -0.45 (0.17) | -0.44 (0.21) | - |
| psi(shrub cover), p(time from sunrise) | -0.9 (0.18) | -0.46 (0.17) | - | - |
| psi(.), p(time from sunrise + canopy cover) | -1.31 (0.22) | -0.44 (0.17) | -0.49 (0.16) | - |
| psi(shrub cover), p(time from sunrise + shrub cover) | -0.94 (0.19) | -0.47 (0.18) | - | 0.08 (0.14) |
| psi(stand basal area + shrub cover), p(time from sunrise) | -0.91 (0.18) | -0.46 (0.17) | - | - |
| psi(distance to PA), p(time from sunrise + canopy cover) | -1.29 (0.23) | -0.44 (0.17) | -0.48 (0.17) | - |
| psi(stand basal area + shrub cover), p(time from sunrise + shrub cover) | -0.95 (0.19) | -0.48 (0.18) | - | 0.08 (0.14) |
| psi(stand basal area), p(time from sunrise + canopy cover) | -1.31 (0.24) | -0.44 (0.17) | -0.5 (0.18) | - |
| psi(.), p(time from sunrise) | -0.94 (0.18) | -0.48 (0.17) | - | - |
| psi(.), p(time from sunrise + shrub cover) | -1.06 (0.21) | -0.52 (0.17) | - | 0.19 (0.14) |
| psi(distance to PA), p(time from sunrise) | -0.93 (0.18) | -0.47 (0.17) | - | - |
| psi(stand basal area), p(time from sunrise) | -0.93 (0.18) | -0.47 (0.17) | - | - |
| psi(stand basal area), p(time from sunrise + shrub cover) | -1.04 (0.2) | -0.51 (0.17) | - | 0.18 (0.14) |
| psi(distance to PA), p(time from sunrise + shrub cover) | -1.04 (0.21) | -0.5 (0.17) | - | 0.17 (0.14) |

Omnivores

| **Model** | **Intercept** | **Time from sunrise** | **Canopy cover** | **Shrub cover** |
| --- | --- | --- | --- | --- |
| **psi(bamboo cover), p(shrub cover)** | **-1.34 (0.17)** | **-** | **-** | **-0.5 (0.16)** |
| **psi(bamboo cover), p(canopy cover + shrub cover)** | **-1.38 (0.18)** | **-** | **-0.2 (0.18)** | **-0.61 (0.19)** |
| **psi(bamboo cover), p(time from sunrise + shrub cover)** | **-1.33 (0.18)** | **-0.15 (0.14)** | **-** | **-0.5 (0.17)** |
| **psi(canopy cover), p(shrub cover)** | **-1.45 (0.15)** | **-** | **-** | **-0.56 (0.17)** |
| **psi(canopy cover), p(time from sunrise + shrub cover)** | **-1.47 (0.14)** | **-0.17 (0.14)** | **-** | **-0.56 (0.17)** |
| **psi(canopy cover + bamboo cover), p(shrub cover)*** | **-1.37 (0.17)** | **-** | **-** | **-0.55 (0.18)** |
| psi(bamboo cover + shrub cover), p(shrub cover) | -1.34 (0.18) | - | - | -0.51 (0.2) |
| psi(canopy cover + bamboo cover), p(canopy cover + shrub cover) | -1.41 (0.19) | - | -0.37 (0.23) | -0.61 (0.19) |
| psi(canopy cover + bamboo cover), p(time from sunrise + shrub cover) | -1.38 (0.18) | -0.15 (0.14) | - | -0.55 (0.18) |
| psi(canopy cover + shrub cover), p(shrub cover) | -1.46 (0.14) | - | - | -0.59 (0.17) |
| psi(canopy cover), p(canopy cover + shrub cover) | -1.48 (0.16) | - | -0.08 (0.18) | -0.6 (0.19) |
| psi(bamboo cover + shrub cover), p(canopy cover + shrub cover) | -1.38 (0.2) | - | -0.2 (0.18) | -0.61 (0.23) |
| psi(.), p(canopy cover + shrub cover) | -1.4 (0.19) | - | -0.31 (0.17) | -0.59 (0.19) |
| psi(canopy cover + shrub cover), p(time from sunrise + shrub cover) | -1.48 (0.14) | -0.18 (0.14) | - | -0.58 (0.17) |
| psi(bamboo cover + shrub cover), p(time from sunrise + shrub cover) | -1.35 (0.19) | -0.15 (0.14) | - | -0.52 (0.19) |
| psi(bamboo cover + shrub cover), p(.) | -1.18 (0.17) | - | - | - |
| psi(canopy cover + bamboo cover + shrub cover), p(shrub cover) | -1.38 (0.18) | - | - | -0.56 (0.19) |
| psi(.), p(shrub cover) | -1.33 (0.19) | - | - | -0.43 (0.17) |
| psi(.), p(time from sunrise + shrub cover) | -1.33 (0.18) | -0.2 (0.14) | - | -0.42 (0.17) |
| psi(canopy cover + bamboo cover + shrub cover), p(canopy cover + shrub cover) | -1.39 (0.21) | - | -0.38 (0.22) | -0.56 (0.26) |
| psi(bamboo cover + shrub cover), p(time from sunrise) | -1.18 (0.18) | -0.14 (0.14) | - | - |
| psi(canopy cover + bamboo cover + shrub cover), p(time from sunrise + shrub cover) | -1.39 (0.19) | -0.15 (0.14) | - | -0.57 (0.19) |
| psi(stand basal area), p(canopy cover + shrub cover) | -1.41 (0.19) | - | -0.36 (0.18) | -0.59 (0.19) |
| psi(canopy cover + bamboo cover + shrub cover + distance to PA), p(shrub cover) | -1.31 (0.25) | - | - | -0.47 (0.34) |
| psi(shrub cover), p(canopy cover + shrub cover) | -1.37 (0.2) | - | -0.33 (0.18) | -0.47 (0.32) |
| psi(canopy cover + bamboo cover + shrub cover + distance to PA), p(.) | -1.17 (0.17) | - | - | - |
| psi(canopy cover + bamboo cover + shrub cover), p(.) | -1.18 (0.17) | - | - | - |
| psi(canopy cover + bamboo cover + shrub cover + distance to PA), p(time from sunrise + shrub cover) | -1.33 (0.29) | -0.15 (0.14) | - | -0.49 (0.33) |
| psi(canopy cover + bamboo cover + shrub cover + distance to PA), p(canopy cover + shrub cover) | -1.3 (0.2) | - | -0.28 (0.27) | -0.48 (0.27) |
| psi(shrub cover), p(shrub cover) | -1.4 (0.2) | - | - | -0.53 (0.19) |
| psi(shrub cover), p(time from sunrise + shrub cover) | -1.39 (0.2) | -0.19 (0.14) | - | -0.53 (0.19) |
| psi(shrub cover), p(.) | -1.26 (0.2) | - | - | - |
| psi(stand basal area), p(shrub cover) | -1.33 (0.19) | - | - | -0.43 (0.17) |
| psi(stand basal area), p(time from sunrise + shrub cover) | -1.33 (0.18) | -0.2 (0.14) | - | -0.43 (0.17) |
| psi(canopy cover + bamboo cover + shrub cover + distance to PA), p(time from sunrise) | -1.17 (0.17) | -0.14 (0.14) | - | - |
| psi(stand basal area + shrub cover), p(canopy cover + shrub cover) | -1.47 (0.19) | - | -0.33 (0.18) | -0.68 (0.2) |
| psi(shrub cover), p(time from sunrise) | -1.25 (0.2) | -0.18 (0.14) | - | - |
| psi(canopy cover + bamboo cover + shrub cover), p(time from sunrise) | -1.18 (0.18) | -0.14 (0.14) | - | - |
| psi(canopy cover + shrub cover), p(.) | -1.22 (0.19) | - | - | - |
| psi(stand basal area + shrub cover), p(.) | -1.26 (0.2) | - | - | - |
| psi(canopy cover + shrub cover), p(time from sunrise) | -1.22 (0.19) | -0.17 (0.14) | - | - |
| psi(stand basal area + shrub cover), p(shrub cover) | -1.41 (0.23) | - | - | -0.54 (0.19) |
| psi(stand basal area + shrub cover), p(time from sunrise + shrub cover) | -1.4 (0.21) | -0.19 (0.14) | - | -0.53 (0.19) |
| psi(stand basala area + shrub cover), p(time from sunrise) | -1.25 (0.2) | -0.18 (0.14) | - | - |
| psi(bamboo cover), p(.) | -1.21 (0.19) | - | - | - |
| psi(bamboo cover), p(time from sunrise) | -1.21 (0.19) | -0.18 (0.14) | - | - |
| psi(.), p(time from sunrise) | -1.23 (0.19) | -0.21 (0.14) | - | - |
| psi(.), p(.) | -1.22 (0.19) | - | - | - |
| psi(canopy cover + bamboo cover), p(.) | -1.21 (0.18) | - | - | - |
| psi(canopy cover + bamboo cover), p(time from sunrise) | -1.21 (0.18) | -0.18 (0.14) | - | - |
| psi(stand basal area), p(time from sunrise) | -1.23 (0.19) | -0.2 (0.14) | - | - |
| psi(distance to PA), p(time from sunrise) | -1.23 (0.19) | -0.21 (0.14) | - | - |
| psi(canopy cover), p(time from sunrise) | -1.23 (0.19) | -0.21 (0.14) | - | - |
| psi(stand basal area), p(.) | -1.23 (0.19) | - | - | - |
| psi(distance to PA), p(.) | -1.22 (0.19) | - | - | - |
| psi(canopy cover), p(.) | -1.22 (0.19) | - | - | - |

Frugivores

| **Model** | **Intercept** | **Time from sunrise** | **Canopy cover** | **Shrub cover** |
| --- | --- | --- | --- | --- |
| **psi(.), p(shrub cover)*** | **1.08 (0.11)** | **-** | **-** | **-0.15 (0.11)** |
| **psi(.), p(.)** | **1.08 (0.11)** | **-** | **-** | **-** |
| psi(.), p(time from sunrise + shrub cover) | 1.08 (0.11) | 0.13 (0.11) | - | -0.17 (0.11) |
| psi(.), p(time from sunrise) | 1.08 (0.11) | 0.11 (0.11) | - | - |
| psi(shrub cover), p(.) | 1.08 (0.11) | - | - | - |
| psi(bamboo cover), p(.) | 1.08 (0.11) | - | - | - |
| psi(shrub cover), p(shrub cover) | 1.08 (0.11) | - | - | -0.14 (0.11) |
| psi(bamboo cover), p(shrub cover) | 1.08 (0.11) | - | - | -0.15 (0.11) |
| psi(distance to PA), p(shrub cover) | 1.08 (0.11) | - | - | -0.14 (0.11) |
| psi(.), p(canopy cover) | 1.08 (0.11) | - | 0.06 (0.11) | - |
| psi(distance to PA), p(.) | 1.04 (0.1) | - | - | - |
| psi(canopy cover), p(.) | 1.08 (0.11) | - | - | - |
| psi(stand basal area), p(.) | 1.08 (0.11) | - | - | - |
| psi(canopy cover), p(shrub cover) | 1.08 (0.11) | - | - | -0.15 (0.11) |
| psi(stand basal area), p(shrub cover) | 1.08 (0.11) | - | - | -0.15 (0.11) |
| psi(shrub cover), p(time from sunrise + shrub cover) | 1.08 (0.11) | 0.12 (0.11) | - | -0.15 (0.11) |
| psi(bamboo cover), p(time from sunrise + shrub cover) | 1.08 (0.11) | 0.12 (0.11) | - | -0.16 (0.11) |
| psi(shrub cover), p(time from sunrise) | 1.08 (0.11) | 0.11 (0.11) | - | - |
| psi(bamboo cover), p(time from sunrise) | 1.08 (0.11) | 0.11 (0.11) | - | - |
| psi(distance to PA), p(time from sunrise + shrub cover) | 1.08 (0.11) | 0.12 (0.11) | - | -0.15 (0.11) |
| psi(distance to PA), p(time from sunrise) | 1.07 (0.11) | 0.11 (0.11) | - | - |
| psi(canopy cover), p(time from sunrise + shrub cover) | 1.08 (0.11) | 0.13 (0.11) | - | -0.17 (0.11) |
| psi(stand basal area), p(time from sunrise + shrub cover) | 1.08 (0.11) | 0.13 (0.11) | - | -0.17 (0.11) |
| psi(bamboo cover + shrub cover), p(.) | 1.08 (0.11) | - | - | - |
| psi(bamboo cover + shrub cover), p(shrub cover) | 1.08 (0.11) | - | - | -0.14 (0.11) |
| psi(shrub cover), p(canopy cover) | 1.08 (0.11) | - | 0.05 (0.11) | - |
| psi(stand basal area), p(time from sunrise) | 1.08 (0.11) | 0.11 (0.11) | - | - |
| psi(canopy cover + shrub cover), p(.) | 1.08 (0.11) | - | - | - |
| psi(bamboo cover), p(canopy cover) | 1.08 (0.11) | - | 0.06 (0.11) | - |
| psi(stand basal area + shrub cover), p(.) | 1.08 (0.11) | - | - | - |
| psi(canopy cover + shrub cover), p(shrub cover) | 1.08 (0.11) | - | - | -0.15 (0.11) |
| psi(distance to PA), p(canopy cover) | 1.08 (0.11) | - | 0.05 (0.11) | - |
| psi(canopy cover + bamboo cover), p(.) | 1.08 (0.11) | - | - | - |
| psi(canopy cover + bamboo cover), p(shrub cover) | 1.08 (0.11) | - | - | -0.15 (0.11) |
| psi(bamboo cover + shrub cover), p(time from sunrise + shrub cover) | 1.08 (0.11) | 0.12 (0.11) | - | -0.15 (0.11) |
| psi(bamboo cover + shrub cover), p(time from sunrise) | 1.07 (0.11) | 0.11 (0.11) | - | - |
| psi(canopy cover), p(canopy cover) | 1.08 (0.11) | - | 0.06 (0.11) | - |
| psi(stand basal area), p(canopy cover) | 1.08 (0.11) | - | 0.06 (0.11) | - |
| psi(canopy cover + shrub cover), p(time from sunrise + shrub cover) | 1.08 (0.11) | 0.12 (0.11) | - | -0.16 (0.11) |
| psi(canopy cover + bamboo cover), p(time from sunrise + shrub cover) | 1.08 (0.11) | 0.12 (0.11) | - | -0.17 (0.12) |
| psi(stand basal area + shrub cover), p(time from sunrise + shrub cover) | 1.08 (0.11) | 0.12 (0.11) | - | -0.15 (0.11) |
| psi(canopy cover), p(time from sunrise) | 1.05 (0.1) | 0.16 (0.11) | - | - |
| psi(stand basal area + shrub cover), p(time from sunrise) | 1.08 (0.11) | 0.11 (0.11) | - | - |
| psi(canopy cover + bamboo cover + shrub cover), p(.) | 1.08 (0.11) | - | - | - |
| psi(canopy cover + bamboo cover), p(time from sunrise) | 1.08 (0.11) | 0.11 (0.11) | - | - |
| psi(canopy cover + bamboo cover + shrub cover), p(shrub cover) | 1.08 (0.11) | - | - | -0.15 (0.11) |
| psi(bamboo cover + shrub cover), p(canopy cover) | 1.08 (0.11) | - | 0.05 (0.11) | - |
| psi(canopy cover + shrub cover), p(canopy cover) | 1.08 (0.11) | - | 0.06 (0.11) | - |
| psi(stand basal area + shrub cover), p(canopy cover) | 1.08 (0.11) | - | 0.05 (0.11) | - |
| psi(canopy cover + shrub cover), p(time from sunrise) | 1.05 (0.1) | 0.16 (0.11) | - | - |
| psi(canopy cover + bamboo cover), p(canopy cover) | 1.08 (0.11) | - | 0.06 (0.11) | - |
| psi(canopy cover + bamboo cover + shrub cover), p(time from sunrise + shrub cover) | 1.08 (0.11) | 0.12 (0.11) | - | -0.16 (0.11) |
| psi(canopy cover + bamboo cover + shrub cover), p(time from sunrise) | 1.08 (0.11) | 0.11 (0.11) | - | - |
| psi(stand basal area + shrub cover), p(shrub cover) | 1.06 (0.1) | - | - | -0.21 (0.1) |
| psi(canopy cover + bamboo cover + shrub cover + distance to PA), p(.) | 1.08 (0.11) | - | - | - |
| psi(canopy cover + bamboo cover + shrub cover), p(canopy cover) | 1.08 (0.11) | - | 0.06 (0.11) | - |
| psi(canopy cover + bamboo cover + shrub cover + distance to PA), p(shrub cover) | 1.08 (0.11) | - | - | -0.14 (0.11) |
| psi(canopy cover + bamboo cover + shrub cover + distance to PA), p(time from sunrise + shrub cover) | 1.08 (0.11) | 0.12 (0.11) | - | -0.15 (0.11) |
| psi(canopy cover + bamboo cover + shrub cover + distance to PA), p(time from sunrise) | 1.08 (0.11) | 0.11 (0.11) | - | - |
| psi(canopy cover + bamboo cover + shrub cover + distance to PA), p(canopy cover) | 1.08 (0.11) | - | 0.06 (0.11) | - |

Large high-canopy gleaning insectivores

| **Model** | **Intercept** | **Time from sunrise** | **Canopy cover** | **Shrub cover** |
| --- | --- | --- | --- | --- |
| **psi(canopy cover), p(time from sunrise)*** | **-0.27 (0.11)** | **-0.16 (0.11)** | **-** | **-** |
| psi(canopy cover + bamboo cover), p(time from sunrise) | -0.29 (0.11) | -0.18 (0.11) | - | - |
| **psi(distance to PA), p(time from sunrise)** | **-0.29 (0.11)** | **-0.17 (0.11)** | **-** | **-** |
| **psi(.), p(time from sunrise)** | **-0.28 (0.11)** | **-0.16 (0.11)** | **-** | **-** |
| psi(canopy cover), p(time from sunrise + shrub cover) | -0.27 (0.11) | -0.16 (0.11) | - | -0.08 (0.1) |
| psi(canopy cover + bamboo cover), p(time from sunrise + shrub cover) | -0.28 (0.11) | -0.18 (0.11) | - | -0.08 (0.1) |
| psi(canopy cover + bamboo cover + shrub cover + distance to PA), p(time from sunrise) | -0.3 (0.11) | -0.18 (0.1) | - | - |
| psi(canopy cover), p(time from sunrise + canopy cover) | -0.28 (0.11) | -0.16 (0.11) | -0.03 (0.11) | - |
| psi(canopy cover + shrub cover), p(time from sunrise) | -0.27 (0.11) | -0.16 (0.11) | - | - |
| psi(canopy cover + bamboo cover), p(time from sunrise + canopy cover) | -0.29 (0.11) | -0.18 (0.11) | -0.02 (0.1) | - |
| psi(canopy cover + bamboo cover + shrub cover), p(time from sunrise) | -0.28 (0.11) | -0.18 (0.11) | - | - |
| psi(distance to PA), p(time from sunrise + shrub cover) | -0.29 (0.11) | -0.17 (0.11) | - | -0.07 (0.1) |
| psi(shrub cover), p(time from sunrise) | -0.28 (0.11) | -0.16 (0.11) | - | - |
| psi(stand basal area), p(time from sunrise) | -0.28 (0.11) | -0.16 (0.11) | - | - |
| psi(.), p(time from sunrise + shrub cover) | -0.28 (0.11) | -0.16 (0.11) | - | -0.07 (0.1) |
| psi(.), p(time from sunrise + canopy cover) | -0.3 (0.11) | -0.15 (0.11) | -0.07 (0.11) | - |
| psi(distance to PA), p(time from sunrise + canopy cover) | -0.3 (0.11) | -0.16 (0.11) | -0.04 (0.11) | - |
| psi(bamboo cover), p(time from sunrise) | -0.28 (0.11) | -0.16 (0.11) | - | - |
| psi(canopy cover + bamboo cover + shrub cover + distance to PA), p(time from sunrise + shrub cover) | -0.3 (0.11) | -0.18 (0.1) | - | -0.08 (0.1) |
| psi(canopy cover + shrub cover), p(time from sunrise + shrub cover) | -0.27 (0.11) | -0.16 (0.11) | - | -0.08 (0.1) |
| psi(canopy cover + bamboo cover + shrub cover), p(time from sunrise + shrub cover) | -0.28 (0.11) | -0.18 (0.11) | - | -0.08 (0.1) |
| psi(shrub cover), p(time from sunrise + shrub cover) | -0.27 (0.11) | -0.16 (0.11) | - | -0.07 (0.1) |
| psi(canopy cover + bamboo cover + shrub cover + distance to PA), p(time from sunrise + canopy cover) | -0.3 (0.11) | -0.18 (0.1) | -0.01 (0.1) | - |
| psi(shrub cover), p(time from sunrise + canopy cover) | -0.29 (0.11) | -0.15 (0.11) | -0.06 (0.11) | - |
| psi(stand basal area), p(time from sunrise + shrub cover) | -0.27 (0.11) | -0.16 (0.11) | - | -0.07 (0.1) |
| psi(stand basal + shrub cover), p(time from sunrise) | -0.28 (0.11) | -0.16 (0.11) | - | - |
| psi(bamboo cover + shrub cover), p(time from sunrise) | -0.28 (0.11) | -0.17 (0.11) | - | - |
| psi(canopy cover + shrub cover), p(time from sunrise + canopy cover) | -0.28 (0.11) | -0.16 (0.11) | -0.03 (0.11) | - |
| psi(stand basal area), p(time from sunrise + canopy cover) | -0.29 (0.11) | -0.15 (0.11) | -0.06 (0.11) | - |
| psi(canopy cover + bamboo cover + shrub cover), p(time from sunrise + canopy cover) | -0.29 (0.11) | -0.18 (0.11) | -0.02 (0.1) | - |
| psi(bamboo cover), p(time from sunrise + canopy cover) | -0.3 (0.11) | -0.15 (0.11) | -0.07 (0.11) | - |
| psi(bamboo cover), p(time from sunrise + shrub cover) | -0.28 (0.11) | -0.16 (0.11) | - | -0.07 (0.1) |
| psi(stand basal area + shrub cover), p(time from sunrise + shrub cover) | -0.27 (0.11) | -0.16 (0.11) | - | -0.07 (0.1) |
| psi(bamboo cover + shrub cover), p(time from sunrise + shrub cover) | -0.28 (0.11) | -0.17 (0.11) | - | -0.07 (0.1) |
| psi(bamboo cover + shrub cover), p(time from sunrise + canopy cover) | -0.3 (0.11) | -0.16 (0.11) | -0.06 (0.1) | - |
| psi(stand basal area + shrub cover), p(time from sunrise + canopy cover) | -0.29 (0.11) | -0.16 (0.11) | -0.05 (0.11) | - |

Large understory gleaning insectivores

| **Model** | **Intercept** | **Time from sunrise** | **Canopy cover** | **Shrub cover** |
| --- | --- | --- | --- | --- |
| **psi(.), p(time from sunrise)*** | **-1.16 (0.16)** | **-0.37 (0.14)** | **-** | **-** |
| psi(canopy cover), p(time from sunrise) | -1.16 (0.17) | -0.36 (0.14) | - | - |
| psi(bamboo cover), p(time from sunrise) | -1.15 (0.16) | -0.37 (0.14) | - | - |
| psi(canopy cover + shrub cover), p(time from sunrise) | -1.21 (0.17) | -0.35 (0.14) | - | - |
| psi(stand basal area), p(time from sunrise) | -1.15 (0.16) | -0.37 (0.14) | - | - |
| psi(.), p(time from sunrise + canopy cover) | -1.16 (0.16) | -0.37 (0.14) | 0.05 (0.13) | - |
| psi(distance to PA), p(time from sunrise) | -1.15 (0.16) | -0.37 (0.14) | - | - |
| psi(.), p(time from sunrise + shrub cover) | -1.16 (0.16) | -0.37 (0.14) | - | 0.01 (0.13) |
| psi(shrub cover), p(time from sunrise) | -1.15 (0.16) | -0.37 (0.14) | - | - |
| psi(canopy cover + bamboo cover), p(time from sunrise) | -1.16 (0.17) | -0.36 (0.14) | - | - |
| psi(canopy cover), p(time from sunrise + canopy cover) | -1.16 (0.17) | -0.35 (0.14) | -0.06 (0.17) | - |
| psi(canopy cover), p(time from sunrise + shrub cover) | -1.16 (0.17) | -0.36 (0.13) | - | 0.05 (0.14) |
| psi(bamboo cover), p(time from sunrise shrub cover) | -1.16 (0.16) | -0.37 (0.13) | - | 0.04 (0.14) |
| psi(canopy cover + shrub cover), p(time frm sunrise + canopy cover) | -1.22 (0.16) | -0.34 (0.13) | -0.08 (0.14) | - |
| psi(bamboo cover + shrub cover), p(time from sunrise) | -1.15 (0.16) | -0.37 (0.14) | - | - |
| psi(bamboo cover), p(time from sunrise + canopy cover) | -1.16 (0.16) | -0.37 (0.14) | 0.01 (0.15) | - |
| psi(stand basal area), p(time from sunrise + canopy cover) | -1.16 (0.16) | -0.38 (0.14) | 0.09 (0.14) | - |
| psi(canopy cover + bamboo cover + shrub cover), p(time from sunrise) | -1.22 (0.17) | -0.35 (0.14) | - | - |
| psi(canopy cover + shrub cover), p(time from sunrise + shrub cover) | -1.2 (0.17) | -0.36 (0.14) | - | -0.04 (0.14) |
| psi(stand basal area + shrub cover), p(time from sunrise) | -1.15 (0.16) | -0.37 (0.14) | - | - |
| psi(stand basal area), p(time from sunrise + shrub cover) | -1.15 (0.16) | -0.37 (0.14) | - | 0 (0.13) |
| psi(distance to PA), p(time from sunrise + canopy cover) | -1.16 (0.16) | -0.37 (0.14) | 0.06 (0.14) | - |
| psi(shrub cover), p(time from sunrise + canopy cover) | -1.16 (0.16) | -0.37 (0.14) | 0.06 (0.14) | - |
| psi(distance to PA), p(time from sunrise + shrub cover) | -1.15 (0.16) | -0.37 (0.14) | - | 0 (0.13) |
| psi(shrub cover), p(time from sunrise + shrub cover) | -1.15 (0.16) | -0.36 (0.14) | - | 0.02 (0.17) |
| psi(canopy cover + bamboo cover + shrub cover + distance to PA), p(time from sunrise) | -1.26 (0.13) | -0.32 (0.13) | - | - |
| psi(canopy cover + bamboo cover), p(time from sunrise + canopy cover) | -1.17 (0.16) | -0.35 (0.13) | -0.08 (0.16) | - |
| psi(canopy cover + bamboo cover), p(time from sunrise + shrub cover) | -1.17 (0.17) | -0.36 (0.13) | - | 0.06 (0.14) |
| psi(canopy cover + bamboo cover + shrub cover), p(time from sunrise + canopy cover) | -1.21 (0.16) | -0.34 (0.13) | -0.09 (0.14) | - |
| psi(bamboo cover + shrub cover), p(time from sunrise + shrub cover) | -1.16 (0.17) | -0.37 (0.14) | - | 0.05 (0.17) |
| psi(bamboo cover + shrub cover), p(time from sunrise + canopy cover) | -1.16 (0.16) | -0.37 (0.14) | 0.02 (0.15) | - |
| psi(stand basal area + shrub cover), p(time from sunrise + canopy cover) | -1.16 (0.16) | -0.37 (0.14) | 0.08 (0.14) | - |
| psi(canopy cover + bamboo cover + shrub cover), p(time from sunrise + shrub cover) | -1.21 (0.17) | -0.35 (0.14) | - | -0.03 (0.14) |
| psi(stand basal area + shrub cover), p(time from sunrise + shrub cover) | -1.15 (0.16) | -0.36 (0.14) | - | 0.04 (0.16) |
| psi(canopy cover + bamboo cover + shrub cover + distance to PA), p(time from sunrise + canopy cover) | -1.24 (0.14) | -0.33 (0.13) | -0.07 (0.14) | - |
| psi(canopy cover + bamboo cover + shrub cover + distance to PA), p(time from sunrise + shrub cover) | -1.26 (0.13) | -0.32 (0.13) | - | -0.03 (0.12) |

Large high-canopy sallying insectivores

| **Model** | **Intercept** | **Time from sunrise** | **Canopy cover** | **Shrub cover** |
| --- | --- | --- | --- | --- |
| **psi(shrub cover), p(time from sunrise + canopy cover)*** | **0.02 (0.11)** | **-0.17 (0.1)** | **0.35 (0.11)** | **-** |
| **psi(shrub cover), p(canopy cover)** | **0.02 (0.11)** | **-** | **0.34 (0.11)** | **-** |
| psi(stand basal area + shrub cover), p(time from sunrise + canopy cover) | 0.02 (0.11) | -0.17 (0.1) | 0.36 (0.11) | - |
| **psi(shrub cover), p(time from sunrise + canopy cover + shrub cover)** | **0.04 (0.11)** | **-0.18 (0.1)** | **0.4 (0.13)** | **0.11 (0.15)** |
| psi(bamboo cover + shrub cover), p(time from sunrise + canopy cover) | 0.02 (0.11) | -0.17 (0.1) | 0.36 (0.11) | - |
| psi(canopy cover + shrub cover), p(time from sunrise + canopy cover) | 0.02 (0.11) | -0.17 (0.1) | 0.36 (0.11) | - |
| psi(stand basal area + shrub cover), p(canopy cover) | 0.01 (0.11) | - | 0.35 (0.11) | - |
| psi(bamboo cover + shrub cover), p(canopy cover) | 0.02 (0.11) | - | 0.35 (0.11) | - |
| psi(stand basal area + shrub cover), p(time from sunrise + canopy cover + shrub cover) | 0.03 (0.11) | -0.18 (0.1) | 0.41 (0.13) | 0.12 (0.15) |
| psi(canopy cover + shrub cover), p(canopy cover) | 0.02 (0.11) | - | 0.35 (0.11) | - |
| psi(bamboo cover + shrub cover), p(time from sunrise + canopy cover + shrub cover) | 0.04 (0.11) | -0.18 (0.1) | 0.41 (0.13) | 0.11 (0.15) |
| psi(canopy cover + shrub cover), p(time from sunrise + canopy cover + shrub cover) | 0.04 (0.11) | -0.18 (0.1) | 0.41 (0.13) | 0.11 (0.15) |
| psi(canopy cover + bamboo cover + shrub cover), p(time from sunrise + canopy cover) | 0.02 (0.11) | -0.17 (0.1) | 0.36 (0.11) | - |
| psi(canopy cover + bamboo cover + shrub cover), p(canopy cover) | 0.02 (0.11) | - | 0.35 (0.11) | - |
| psi(canopy cover + bamboo cover + shrub cover), p(time from sunrise + canopy cover + shrub cover) | 0.04 (0.11) | -0.18 (0.1) | 0.41 (0.13) | 0.11 (0.15) |
| psi(canopy cover + bamboo cover + shrub cover + distance to PA), p(time from sunrise + canopy cover) | 0.02 (0.11) | -0.17 (0.1) | 0.36 (0.11) | - |
| psi(canopy cover + bamboo cover + shrub cover + distance to PA), p(canopy cover) | 0.02 (0.11) | - | 0.35 (0.11) | - |
| psi(canopy cover + bamboo cover + shrub cover + distance to PA), p(time from sunrise + canopy cover + shrub cover) | 0.03 (0.11) | -0.18 (0.1) | 0.41 (0.13) | 0.12 (0.15) |
| psi(.), p(time from sunrise + canopy cover) | -0.01 (0.11) | -0.17 (0.1) | 0.41 (0.11) | - |
| psi(canopy cover), p(time from sunrise + canopy cover) | 0.02 (0.11) | -0.18 (0.1) | 0.37 (0.11) | - |
| psi(distance to PA), p(time from sunrise + canopy cover) | 0 (0.11) | -0.17 (0.1) | 0.39 (0.11) | - |
| psi(.), p(canopy cover) | 0 (0.11) | - | 0.4 (0.11) | - |
| psi(canopy cover), p(canopy cover) | 0.02 (0.11) | - | 0.36 (0.11) | - |
| psi(.), p(time from sunrise + canopy cover + shrub cover) | -0.03 (0.11) | -0.17 (0.1) | 0.37 (0.13) | -0.11 (0.15) |
| psi(distance to PA), p(canopy cover) | 0 (0.11) | - | 0.38 (0.11) | - |
| psi(bamboo cover), p(time from sunrise + canopy cover) | 0 (0.11) | -0.18 (0.1) | 0.4 (0.11) | - |
| psi(stand basal area), p(time from sunrise + canopy cover) | 0 (0.11) | -0.17 (0.1) | 0.4 (0.11) | - |
| psi(canopy cover + bamboo cover), p(time from sunrise + canopy cover) | 0.02 (0.11) | -0.18 (0.1) | 0.37 (0.11) | - |
| psi(canopy cover), p(time from sunrise + canopy cover + shrub cover) | 0.01 (0.12) | -0.18 (0.1) | 0.36 (0.13) | -0.03 (0.19) |
| psi(distance to PA), p(time from sunrise + canopy cover + shrub cover) | -0.02 (0.12) | -0.17 (0.1) | 0.36 (0.13) | -0.08 (0.17) |
| psi(stand basal area), p(canopy cover) | 0 (0.11) | - | 0.39 (0.11) | - |
| psi(bamboo cover), p(canopy cover) | 0 (0.11) | - | 0.39 (0.11) | - |
| psi(canopy cover + bamboo cover), p(canopy cover) | 0.02 (0.11) | - | 0.36 (0.11) | - |
| psi(bamboo cover), p(time from sunrise + canopy cover + shrub cover) | -0.03 (0.12) | -0.17 (0.11) | 0.37 (0.13) | -0.1 (0.17) |
| psi(stand basal area), p(time from sunrise + canopy cover + shrub cover) | -0.03 (0.12) | -0.17 (0.1) | 0.37 (0.13) | -0.11 (0.17) |
| psi(canopy cover + bamboo cover), p(time from sunrise + canopy cover + shrub cover) | 0.01 (0.12) | -0.18 (0.1) | 0.36 (0.13) | -0.04 (0.19) |

Small mid-canopy gleaning insectivores

| **Model** | **Intercept** | **Time from sunrise** | **Canopy cover** | **Shrub cover** |
| --- | --- | --- | --- | --- |
| **psi(distance to PA), p(time from sunrise + shrub cover)*** | **0.45 (0.1)** | **-0.37 (0.1)** | **-** | **-0.1 (0.1)** |
| **psi(.), p(time from sunrise)** | **0.44 (0.1)** | **-0.38 (0.1)** | **-** | **-** |
| **psi(.), p(time from sunrise + shrub cover)** | **0.45 (0.1)** | **-0.37 (0.1)** | **-** | **-0.1 (0.1)** |
| psi(shrub cover), p(time from sunrise) | 0.44 (0.1) | -0.38 (0.1) | - | - |
| psi(canopy cover), p(time from sunrise) | 0.44 (0.1) | -0.39 (0.1) | - | - |
| psi(stand basal area), p(time from sunrise) | 0.44 (0.1) | -0.38 (0.1) | - | - |
| psi(bamboo cover), p(time from sunrise) | 0.44 (0.1) | -0.38 (0.1) | - | - |
| psi(shrub cover), p(time from sunrise + shrub cover) | 0.45 (0.1) | -0.37 (0.1) | - | -0.1 (0.1) |
| psi(bamboo cover + shrub cover), p(time from sunrise) | 0.45 (0.1) | -0.38 (0.1) | - | - |
| psi(canopy cover + bamboo cover + shrub cover + distance to PA), p(time from sunrise) | 0.46 (0.1) | -0.37 (0.1) | - | - |
| psi(canopy cover + shrub cover), p(time from sunrise) | 0.44 (0.1) | -0.37 (0.1) | - | - |
| psi(bamboo cover), p(time from sunrise + shrub cover) | 0.45 (0.1) | -0.37 (0.1) | - | -0.1 (0.1) |
| psi(stand basal area), p(time from sunrise + shrub cover) | 0.45 (0.1) | -0.37 (0.1) | - | -0.1 (0.1) |
| psi(canopy cover), p(time from sunrise + shrub cover) | 0.44 (0.1) | -0.38 (0.1) | - | -0.1 (0.1) |
| psi(stand basal area + shrub cover), p(time from sunrise) | 0.44 (0.1) | -0.38 (0.1) | - | - |
| psi(canopy cover + bamboo cover + shrub cover + distance to PA), p(time from sunrise + shrub cover) | 0.47 (0.1) | -0.36 (0.1) | - | -0.11 (0.1) |
| psi(bamboo cover + shrub cover), p(time from sunrise + shrub cover) | 0.45 (0.1) | -0.37 (0.1) | - | -0.11 (0.1) |
| psi(canopy cover + bamboo cover), p(time from sunrise) | 0.44 (0.1) | -0.39 (0.1) | - | - |
| psi(canopy cover + shrub cover), p(time from sunrise + shrub cover) | 0.45 (0.1) | -0.36 (0.1) | - | -0.1 (0.1) |
| psi(stand basal area + shrub cover), p(time from sunrise + shrub cover) | 0.45 (0.1) | -0.37 (0.1) | - | -0.1 (0.1) |
| psi(canopy cover + bamboo cover), p(time from sunrise + shrub cover) | 0.45 (0.1) | -0.37 (0.1) | - | -0.1 (0.1) |
| psi(canopy cover + bamboo cover + shrub cover), p(time from sunrise) | 0.45 (0.1) | -0.37 (0.1) | - | - |
| psi(canopy cover + bamboo cover + shrub cover), p(time from sunrise + shrub cover) | 0.45 (0.1) | -0.36 (0.1) | - | -0.11 (0.1) |

Small understory gleaning insectivores

| **Model** | **Intercept** | **Time from sunrise** | **Canopy cover** | **Shrub cover** |
| --- | --- | --- | --- | --- |
| **psi(canopy cover), p(time from sunrise + canopy cover + shrub cover)*** | **-0.22 (0.12)** | **-0.3 (0.12)** | **-0.36 (0.14)** | **0.31 (0.12)** |
| **psi(stand basal area), p(time from sunrise + canopy cover + shrub cover)** | **-0.28 (0.12)** | **-0.3 (0.11)** | **-0.42 (0.13)** | **0.31 (0.12)** |
| **psi(.), p(time from sunrise + canopy cover + shrub cover)** | **-0.33 (0.11)** | **-0.33 (0.11)** | **-0.48 (0.12)** | **0.31 (0.12)** |
| psi(canopy cover + bamboo cover), p(time from sunrise + canopy cover + shrub cover) | -0.22 (0.12) | -0.31 (0.12) | -0.37 (0.14) | 0.3 (0.12) |
| psi(canopy cover + shrub cover), p(time from sunrise + canopy cover + shrub cover) | -0.22 (0.12) | -0.3 (0.12) | -0.36 (0.14) | 0.31 (0.13) |
| psi(stand basal area + shrub cover), p(time from sunrise + canopy cover + shrub cover) | -0.26 (0.12) | -0.3 (0.11) | -0.41 (0.13) | 0.3 (0.12) |
| psi(shrub cover), p(time from sunrise + canopy cover + shrub cover) | -0.3 (0.12) | -0.32 (0.11) | -0.46 (0.13) | 0.29 (0.13) |
| psi(bamboo cover), psi(time from sunrise + canopy cover + shrub cover) | -0.32 (0.11) | -0.32 (0.11) | -0.47 (0.13) | 0.31 (0.12) |
| psi(distance to PA), p(time from sunrise + canopy cover + shrub cover) | -0.33 (0.11) | -0.33 (0.11) | -0.48 (0.13) | 0.31 (0.12) |
| psi(canopy cover + bamboo cover + shrub cover), p(time from sunrise + canopy cover + shrub cover) | -0.22 (0.12) | -0.31 (0.12) | -0.37 (0.14) | 0.3 (0.13) |
| psi(bamboo cover + shrub cover), p(time from sunrise + canopy cover + shrub cover) | -0.3 (0.12) | -0.33 (0.11) | -0.46 (0.13) | 0.29 (0.13) |
| psi(canopy cover + bamboo cover + shrub cover + distance to PA), p(time from sunrise + canopy cover + shrub cover) | -0.21 (0.12) | -0.29 (0.12) | -0.36 (0.14) | 0.3 (0.13) |

Small mid-canopy sallying insectivores

| **Model** | **Intercept** | **Time from sunrise** | **Canopy cover** | **Shrub cover** |
| --- | --- | --- | --- | --- |
| **psi(bamboo cover + shrub cover), p(canopy cover + shrub cover)*** | **-0.24 (0.12)** | **-** | **0.81 (0.14)** | **0.4 (0.14)** |
| **psi(bamboo cover), p(canopy cover + shrub cover)** | **-0.23 (0.11)** | **-** | **0.8 (0.14)** | **0.36 (0.14)** |
| psi(canopy cover + bamboo cover), p(canopy cover + shrub cover) | -0.21 (0.11) | - | 0.77 (0.15) | 0.36 (0.14) |
| psi(shrub cover), p(canopy cover + shrub cover) | -0.25 (0.12) | - | 0.84 (0.14) | 0.4 (0.14) |
| psi(canopy cover), p(canopy cover + shrub cover) | -0.21 (0.12) | - | 0.77 (0.15) | 0.36 (0.15) |
| psi(stand basal area + shrub cover), p(canopy cover + shrub cover) | -0.24 (0.12) | - | 0.84 (0.14) | 0.4 (0.14) |
| psi(canopy cover + shrub cover), p(canopy cover + shrub cover) | -0.23 (0.12) | - | 0.82 (0.15) | 0.4 (0.14) |
| psi(.), p(canopy cover + shrub cover) | -0.26 (0.12) | - | 0.84 (0.14) | 0.34 (0.15) |
| psi(stand basal area), p(canopy cover + shrub cover) | -0.24 (0.12) | - | 0.83 (0.14) | 0.36 (0.14) |
| psi(distance to PA), p(canopy cover + shrub cover) | -0.26 (0.12) | - | 0.84 (0.14) | 0.32 (0.15) |

Large woodpeckers

| **Model** | **Intercept** | **Time from sunrise** | **Canopy cover** | **Shrub cover** |
| --- | --- | --- | --- | --- |
| **psi(shrub cover), p(time from sunrise + canopy cover)*** | **-1.45 (0.14)** | **-0.3 (0.13)** | **1 (0.14)** | **-** |
| **psi(.), p(time from sunrise + canopy cover)** | **-1.38 (0.15)** | **-0.29 (0.13)** | **0.96 (0.15)** | **-** |
| psi(stand basal area + shrub cover), p(time from sunrise + canopy cover) | -1.47 (0.14) | -0.31 (0.13) | 1.01 (0.14) | - |
| psi(shrub cover), p(time from sunrise + canopy cover + shrub cover) | -1.44 (0.14) | -0.27 (0.13) | 0.91 (0.17) | -0.18 (0.19) |
| psi(distance to PA), p(time from sunrise + canopy cover) | -1.35 (0.15) | -0.28 (0.13) | 0.95 (0.15) | - |
| psi(canopy cover + shrub cover), p(time from sunrise + canopy cover) | -1.41 (0.16) | -0.29 (0.13) | 0.98 (0.16) | - |
| psi(bamboo cover + shrub cover), p(time from sunrise + canopy cover) | -1.45 (0.14) | -0.29 (0.13) | 1 (0.14) | - |
| psi(canopy cover + shrub cover), p(time from sunrise + canopy cover + shrub cover) | -1.35 (0.17) | -0.26 (0.13) | 0.79 (0.21) | -0.27 (0.21) |
| psi(stand basal area), p(time from sunrise + canopy cover) | -1.42 (0.16) | -0.29 (0.13) | 0.99 (0.15) | - |
| psi(.), p(time from sunrise + canopy cover + shrub cover) | -1.38 (0.15) | -0.28 (0.13) | 0.92 (0.18) | -0.09 (0.19) |
| psi(stand basal area + shrub cover), p(time from sunrise + canopy cover + shrub cover) | -1.46 (0.14) | -0.29 (0.13) | 0.94 (0.18) | -0.14 (0.2) |
| psi(canopy cover), p(time from sunrise + canopy cover) | -1.41 (0.17) | -0.29 (0.13) | 0.99 (0.16) | - |
| psi(bamboo cover), p(time from sunrise + canopy cover) | -1.39 (0.16) | -0.29 (0.13) | 0.97 (0.15) | - |
| psi(canopy cover + bamboo cover + shrub cover), p(time from sunrise + canopy cover + shrub cover) | -1.36 (0.15) | -0.25 (0.13) | 0.79 (0.2) | -0.29 (0.21) |
| psi(canopy cover + bamboo cover + shrub cover), p(time from sunrise + canopy cover) | -1.4 (0.15) | -0.28 (0.13) | 0.96 (0.15) | - |
| psi(bamboo cover + shrub cover), p(time from sunrise + canopy cover + shrub cover) | -1.44 (0.14) | -0.27 (0.13) | 0.92 (0.17) | -0.17 (0.19) |
| psi(shrub cover), p(canopy cover + shrub cover) | -1.44 (0.14) | - | 0.83 (0.16) | -0.25 (0.19) |
| psi(distance to PA), p(time from sunrise + canopy cover + shrub cover) | -1.35 (0.15) | -0.28 (0.13) | 0.93 (0.18) | -0.04 (0.19) |
| psi(canopy cover + shrub cover), p(canopy cover + shrub cover) | -1.34 (0.16) | - | 0.69 (0.2) | -0.36 (0.21) |
| psi(canopy cover + bamboo cover + shrub cover), p(canopy cover + shrub cover) | -1.37 (0.16) | - | 0.7 (0.18) | -0.34 (0.21) |
| psi(stand basal area), p(time from sunrise + canopy cover + shrub cover) | -1.41 (0.17) | -0.29 (0.13) | 0.95 (0.19) | -0.07 (0.19) |
| psi(canopy cover), p(time from sunrise + canopy cover + shrub cover) | -1.41 (0.17) | -0.29 (0.13) | 0.94 (0.19) | -0.08 (0.19) |
| psi(bamboo cover), p(time from sunrise + canopy cover + shrub cover) | -1.38 (0.16) | -0.28 (0.13) | 0.92 (0.18) | -0.09 (0.19) |
| psi(canopy cover + bamboo cover), p(time from sunrise + canopy cover) | -1.42 (0.17) | -0.29 (0.13) | 0.99 (0.16) | - |
| psi(canopy cover + bamboo cover + shrub cover + distance to PA), p(time from sunrise + canopy cover) | -1.4 (0.14) | -0.29 (0.13) | 0.96 (0.15) | - |
| psi(canopy cover + bamboo cover + shrub cover + distance to PA), p(time from sunrise + canopy cover + shrub cover) | -1.36 (0.15) | -0.25 (0.13) | 0.79 (0.2) | -0.28 (0.21) |
| psi(.), p(canopy cover + shrub cover) | -1.37 (0.16) | - | 0.83 (0.17) | -0.14 (0.19) |
| psi(bamboo cover + shrub cover), p(canopy cover, shrub cover) | -1.44 (0.14) | - | 0.83 (0.16) | -0.24 (0.19) |
| psi(stand basal area + shrub cover), p(canopy cover + shrub cover) | -1.44 (0.14) | - | 0.83 (0.17) | -0.24 (0.19) |
| psi(canopy cover + bamboo cover + shrub cover + distance to PA), p(canopy cover + shrub cover) | -1.38 (0.17) | - | 0.7 (0.18) | -0.34 (0.23) |
| psi(distance to PA), p(canopy cover + shrub cover) | -1.35 (0.16) | - | 0.85 (0.17) | -0.07 (0.2) |
| psi(canopy cover + bamboo cover), p(time from sunrise + canopy cover + shrub cover) | -1.41 (0.18) | -0.29 (0.13) | 0.94 (0.2) | -0.08 (0.19) |
| psi(bamboo cover), p(canopy cover + shrub cover) | -1.38 (0.16) | - | 0.83 (0.17) | -0.14 (0.19) |
| psi(canopy cover), p(canopy cover + shrub cover) | -1.38 (0.19) | - | 0.84 (0.19) | -0.14 (0.19) |
| psi(stand basal area), p(canopy cover + shrub cover) | -1.38 (0.2) | - | 0.83 (0.21) | -0.14 (0.2) |
| psi(canopy cover + bamboo cover), p(canopy cover + shrub cover) | -1.38 (0.19) | - | 0.83 (0.2) | -0.14 (0.19) |
