# Supplementary material for: “In a tree by the brook, there’s a songbird who sings”: Woodlands in an agricultural matrix maintain functionality of a wintering bird community
Source: PLoS One. 2018 Aug 2;13(8):e0201657. doi: 10.1371/journal.pone.0201657 (PMC6072076; doi:10.1371/journal.pone.0201657)
Supplement: S5 File — Nomenclature follows Grimmett et al. (2011). (DOCX) [file pone.0201657.s005.docx]

**S5 File**. **List of bird species recorded during the study period, along with their**

**assigned guilds.** Nomenclature follows Grimmett *et al.* (2011).

| *Sl. No.* | *Scientific name* | *English name* | *Guild* |
| --- | --- | --- | --- |
| 1 | *Aethopyga ignicauda* | Fire-tailed Sunbird | Nectarivore |
| 2 | *Aethopyga siparaja* | Crimson Sunbird | Nectarivore |
| 3 | *Arachnothera longistra* | Little Spiderhunter | Nectarivore |
| 4 | *Arachnothera magna* | Streaked Spiderhunter | Nectarivore |
| 5 | *Chalcoparia singalensis* | Ruby-cheeked Sunbird | Nectarivore |
| 6 | *Dicaeum cruentatum* | Scarlet-backed Flowerpecker | Nectarivore |
| 7 | *Dicaeum ignipectus* | Fire-breasted Flowerpecker | Nectarivore |
| 8 | *Dicaeum minullum* | Plain Flowerpecker | Nectarivore |
| 9 | *Lonchura striata* | White-rumped Munia | Granivore |
| 10 | *Streptopelia decaocto* | Eurasian Collared Dove | Granivore |
| 11 | *Streptopelia orientalis* | Oriental Turtle Dove | Granivore |
| 12 | *Centropus sinensis* | Greater coucal | Omnivore |
| 13 | *Corvus levaillantii* | Eastern Jungle Crow | Omnivore |
| 14 | *Dendrocitta formosae* | Grey Treepie | Omnivore |
| 15 | *Gracula religiosa* | Common Hill Myna | Omnivore |
| 16 | *Rhopodytes tristis* | Green-billed Malkoha | Omnivore |
| 17 | *Sturnia malabarica* | Chestnut-tailed Starling | Omnivore |
| 18 | *Alophoixus flaveolus* | White-throated Bulbul | Frugivore |
| 19 | *Chalcophaps indica* | Emerald Dove | Frugivore |
| 20 | *Hemixos flavala* | Ashy Bulbul | Frugivore |
| 21 | *Hypsipetes leucocephalus* | Black Bulbul | Frugivore |
| 22 | *Irena puella* | Asian Fairy Bluebird | Frugivore |
| 23 | *Megalaima asiatica* | Blue-throated Barbet | Frugivore |
| 24 | *Megalaima australis* | Blue-eared Barbet | Frugivore |
| 25 | *Megalaima lineata* | Lineated Barbet | Frugivore |
| 26 | *Megalaima virens* | Great Barbet | Frugivore |
| 27 | *Oriolus traillii* | Maroon Oriole | Frugivore |
| 28 | *Oriolus xanthornus* | Black-hooded Oriole | Frugivore |
| 29 | *Pycnonotus cafer* | Red-vented Bulbul | Frugivore |
| 30 | *Pycnonotus flaviventris* | Black-crested Bulbul | Frugivore |
| 31 | *Pycnonotus jocosus* | Red-whiskered Bulbul | Frugivore |
| 32 | *Chloropsis aurifrons* | Golden-fronted Leafbird | Large high-canopy gleaning insectivore |
| 33 | *Coracina macei* | Large Cuckooshrike | Large high-canopy gleaning insectivore |
| 34 | *Cuculus Micropterus* | Indian Cuckoo | Large high-canopy gleaning insectivore |
| 35 | *Hierococcyx spp.* | Hawk Cuckoo | Large high-canopy gleaning insectivore |
| 36 | *Melanochlora sultanea* | Sultan Tit | Large high-canopy gleaning insectivore |
| 37 | *Pericrocotus speciosus* | Scarlet Minivet | Large high-canopy gleaning insectivore |
| 38 | *Psarisomus dalhousiae* | Long-tailed Broadbill | Large high-canopy gleaning insectivore |
| 39 | *Chrysomma sinense* | Yellow-eyed Babbler | Large low-canopy gleaning insectivore |
| *Sl. No.* | *Scientific name* | *English name* | *Guild* |
| 40 | *Copsychus malabaricus* | White-rumped Shama | Large low-canopy gleaning insectivore |
| 41 | *Copsychus saularis* | Oriental Magpie Robin | Large low-canopy gleaning insectivore |
| 42 | *Gampsorhynchus rufulus* | White-hooded Babbler | Large low-canopy gleaning insectivore |
| 43 | *Garrulax leucolophus* | White-crested Laughingthrush | Large low-canopy gleaning insectivore |
| 44 | *Garrulax monileger* | Lesser Necklaced Laughingthrush | Large low-canopy gleaning insectivore |
| 45 | *Garrulax ruficollis* | Rufous-necked Laughingthrush | Large low-canopy gleaning insectivore |
| 46 | *Myophonus caeruleus* | Blue Whistling Thrush | Large low-canopy gleaning insectivore |
| 47 | *Pomatorhinus schisticeps* | White-browed Scimitar Babbler | Large low-canopy gleaning insectivore |
| 48 | *Turdoides striata* | Jungle Babbler | Large low-canopy gleaning insectivore |
| 49 | *Dicrurus aeneus* | Bronzed Drongo | Large high-canopy sallying insectivore |
| 50 | *Dicrurus hottentottus* | Spangled Drongo | Large high-canopy sallying insectivore |
| 51 | *Dicrurus leucophaeus* | Ashy Drongo | Large high-canopy sallying insectivore |
| 52 | *Dicrurus macrocercus* | Black Drongo | Large high-canopy sallying insectivore |
| 53 | *Dicrurus paradiseus* | Greater Racket-tailed Drongo | Large high-canopy sallying insectivore |
| 54 | *Dicrurus remifer* | Lesser Racket-tailed Drongo | Large high-canopy sallying insectivore |
| 55 | *Eurystomus orientalis* | Dollarbird | Large high-canopy sallying insectivore |
| 56 | *Nyctyornis athertoni* | Blue-bearded Bee-eater | Large high-canopy sallying insectivore |
| 57 | *Tephrodornis virgatus* | Large Woodshrike | Large high-canopy sallying insectivore |
| 58 | *Abroscopus superciliaris* | Yellow-bellied Warbler | Small mid-canopy gleaning insectivore |
| 59 | *Aegithina tiphia* | Common Iora | Small mid-canopy gleaning insectivore |
| 60 | *Erpornis zantholeuca* | White-bellied Erpornis | Small mid-canopy gleaning insectivore |
| 61 | *Phylloscopus inornatus* | Yellow-browed Warbler | Small mid-canopy gleaning insectivore |
| 62 | *Phylloscopus trochiloides* | Greenish Warbler | Small mid-canopy gleaning insectivore |
| 63 | *Seicercus whistleri* | Whistler's Warbler | Small mid-canopy gleaning insectivore |
| 64 | *Sitta frontalis* | Velvet-fronted Nuthatch | Small mid-canopy gleaning insectivore |
| 65 | *Zosterops palpebrosus* | Oriental White-eye | Small mid-canopy gleaning insectivore |
| 66 | *Alcippe nipalensis* | Nepal Fulvetta | Small low-canopy gleaning insectivore |
| 67 | *Luscinia calliope* | Siberian Rubythroat | Small low-canopy gleaning insectivore |
| 68 | *Orthotomus sutorius* | Common Tailorbird | Small low-canopy gleaning insectivore |
| 69 | *Pellorneum ruficeps* | Puff-throated Babbler | Small low-canopy gleaning insectivore |
| 70 | *Prinia hodgsonii* | Grey-breasted Prinia | Small low-canopy gleaning insectivore |
| 71 | *Prinia rufescens* | Rufescent Prinia | Small low-canopy gleaning insectivore |
| 72 | *Stachyridopsis ruficeps* | Rufous-capped Babbler | Small low-canopy gleaning insectivore |
| 73 | *Culicicapa ceylonensis* | Grey-headed Canary Flycatcher | Small mid-canopy sallying insectivore |
| 74 | *Cyornis poliogenys* | Pale-chinned Flycatcher | Small mid-canopy sallying insectivore |
| 75 | *Cyornis rubeculoides* | Blue-throated Blue Flycatcher | Small mid-canopy sallying insectivore |
| 76 | *Cyornis unicolor* | Pale-blue Flycatcher | Small mid-canopy sallying insectivore |
| 77 | *Ficedula albicilla* | Taiga Flycatcher | Small mid-canopy sallying insectivore |
| 78 | *Ficedula westermanni* | Little Pied Flycatcher | Small mid-canopy sallying insectivore |
| 79 | *Hemipus picatus* | Bar-winged Flycatcher-Shrike | Small mid-canopy sallying insectivore |
| 80 | *Hypothymis azurea* | Black-naped Monarch | Small mid-canopy sallying insectivore |
| 81 | *Niltava sundara* | Rufous-bellied Niltava | Small mid-canopy sallying insectivore |
| *Sl. No.* | *Scientific name* | *English name* | *Guild* |
| 82 | *Rhipidura albicollis* | White-throated Fantail | Small mid-canopy sallying insectivore |
| 83 | *Blythipicus pyrrhotis* | Bay Woodpecker | Large woodpecker |
| 84 | *Chrysocolaptes lucidus* | Greater Goldenback | Large woodpecker |
| 85 | *Micropternus brachyurus* | Rufous Woodpecker | Large woodpecker |
| 86 | *Mulleripicus pulverulentus* | Great Slaty Woodpecker | Large woodpecker |
| 87 | *Picus canus* | Grey-headed Woodpecker | Large woodpecker |
| 88 | *Picus chlorolophus* | Lesser Yellownape | Large woodpecker |
| 89 | *Picus flavinucha* | Greater Yellownape | Large woodpecker |
| 90 | *Dendrocopus nanus* | Grey-capped Pygmy Woodpecker | Other |
| 91 | *Picumnus innominatus* | Speckled Piculet | Other |
| 92 | *Sasia ochracea* | White-browed Piculet | Other |
| 93 | *Glaucidium cuculoides* | Asian Barred Owlet | - |
| 94 | *Lanius tephronotus* | Grey-backed Shrike | Other |
| 95 | *Spilornis cheela* | Crested Serpent Eagle | - |
| 96 | *Anthracoceros albirostris* | Oriental Pied Hornbill | Other |
| 97 | *Buceros bicornis* | Great Hornbill | Other |
